# Design, synthesis and evaluation of pyrrolobenzodiazepine (PBD)-based PROTAC conjugates for the selective degradation of the NF-κB RelA/p65 subunit†

**DOI:** 10.1039/d5md00316d

**Published:** 2025-05-08

**Authors:** Peiqin Jin, Md. Mahbub Hasan, Andrea G. S. Pepper, Simon Mitchell, Khondaker Miraz Rahman, Chris Pepper

**Affiliations:** a Institute of Pharmaceutical Science, School of Cancer and Pharmaceutical Sciences, King's College London London SE1 9NH UK k.miraz.rahman@kcl.ac.uk; b Brighton and Sussex Medical School, University of Brighton and University of Sussex Brighton BN1 9PX UK c.pepper@bsms.ac.uk

## Abstract

NF-κB signalling is frequently dysregulated in human cancers making it an attractive therapeutic target. Despite concerted efforts to generate NF-κB inhibitors, direct pharmacological inhibition of the kinases mediating canonical NF-κB has failed due to on-target toxicities in normal tissues. So, alternative strategies, designed to target specific components of the NF-κB signalling machinery, have the potential to selectively inhibit tumour cells whilst reducing the toxicities associated with broad inhibition of NF-κB in non-malignant cells. Here we present evidence that a C8-linked pyrrolobenzodiazepine (PBD) containing proteolysis-targeting chimera (PROTAC) selectively degrades the NF-κB subunit, RelA/p65, in a proteasome-dependent manner. Our lead PROTAC (JP-163-16, 15d) showed cytotoxicity with mean LC_50_ values of 2.9 μM in MDA-MB-231 cells, 0.14 μM in MEC-1 cells and 0.23 μM in primary chronic lymphocytic leukaemia cells. In contrast, 15d was two-logs less toxic in primary B- and T-lymphocytes (mean LD_50_ 19.1 μM and 36.4 μM, respectively). Importantly, the development of 15d, by conjugating the C8-linked PBD with a cereblon-targeting ligand using a five-carbon linker, abolished the ability of the C8-linked PBD to bind to DNA, whilst demonstrating cytotoxicity in cancer cells associated with the degradation of RelA/p65. Mechanistically, 15d displayed PROTAC credentials through the selective degradation of NF-κB RelA/p65 in a proteasome-dependent manner and showed a five-fold reduction in potency in the cereblon deficient, lenalidomide resistant, myeloma cell line, RPMI-8226. To our knowledge, this work describes the first PROTAC capable of selective degradation of a single NF-κB subunit and highlights the therapeutic potential of our strategy for the treatment of RelA/p65-dependent tumours.

## Abstract 2

Here we describe the design, synthesis and validation of the first NF-κB PROTAC degrader with demonstrable subunit selectivity for RelA/p65.

## Graphical abstract

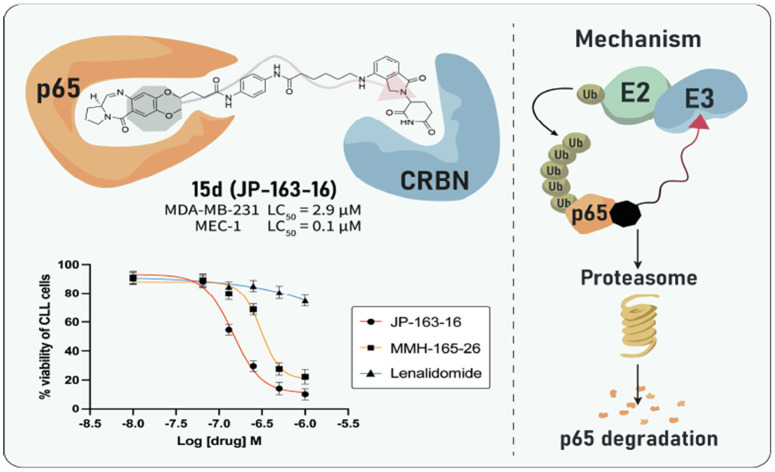

## Introduction

The nuclear factor kappa-light chain-enhancer of activated B-cells (NF-κB) is a transcription factor that plays a pivotal role in inflammatory and immune responses, and its abnormal activation is associated with various pathogenic effects.^1,2^ For example, an over-expressed level of NF-κB signalling contributes to metastasis in triple-negative breast cancer (TNBC).^3^ In haematological malignancies, elevated nuclear expression of the NF-κB subunit RelA/p65 is commonly associated with more aggressive tumour cell growth, increased tumour burden and the emergence of drug resistance.^4^ Given the pivotal role that NF-κB plays in the development and progression of a range of human cancers, it seems logical to develop strategies to target abnormal NF-κB signalling for the treatment of these diseases.^5^

**Fig. 1:**
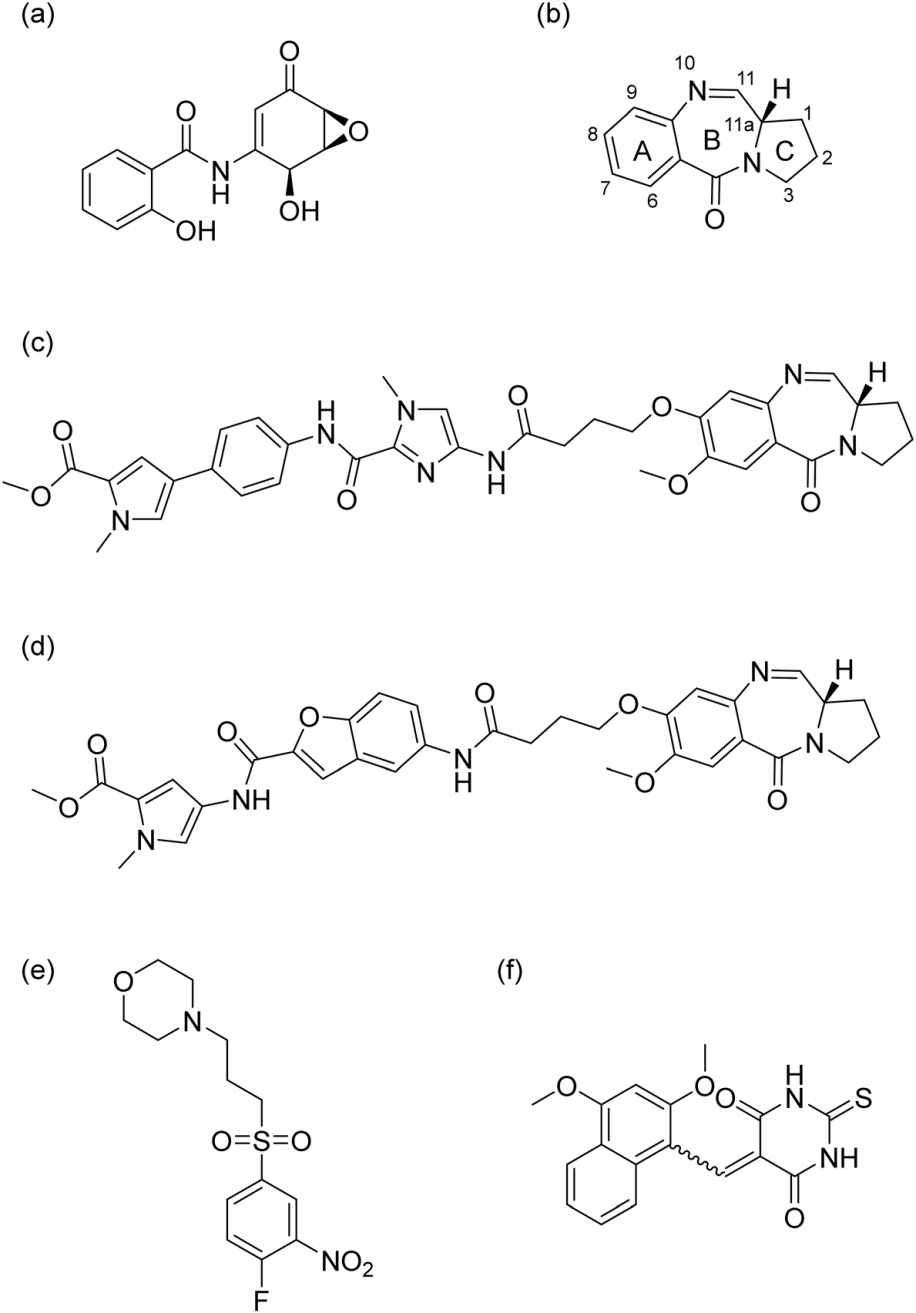
Chemical structure of reported NF-κB targeting compounds (a) (−)-DHMEQ, (b) PBD core, (c) KMR-28-39 (TSG-1301), (d) Cpd 13, (e) CRL1101, and (f) IT-901.

However, direct targeting of transcription factors (TFs) has long been considered intractable due to the lack of well-defined enzymatic binding pockets and limited H-bond donors and acceptors, rendering them ‘undruggable’.^6,7^ To date, only a small number of molecules have been shown to be capable of interacting with NF-κB.^8^ One such molecule, (−)-DHMEQ, a synthetic compound derived from epoxyquinomicin C, can inhibit the nuclear transport of the transcriptional subunit RelA/p65 (Fig. 1a).^9^ Unfortunately, further development of this compound series was halted due to pharmacokinetic issues.^10^ Pyrrolobenzodiazepines (PBDs) (Fig. 1b) are highly cytotoxic agents derived from *Streptomyces*.^11^ They exert their primary anti-tumour effect by selectively binding into the minor groove of DNA in a sequence-selective manner. Subsequent studies showed that these compounds were also able to target DNA motifs related to TF signalling.^11,12^ Hu *et al.* synthesized a C8-conjugated PBD hybrid named IN6CPBD, and cellular studies revealed that this compound induced cellular apoptosis in A375 cells by repressing activation of NF-κB.^13^ Similarly, KMR-28-39 (also named ‘TSG-1301’) was shown to interfere with the binding of NF-κB protein to its cognate DNA motifs^11,14^ (Fig. 1c). This competitive inhibition resulted in nanomolar cytotoxicity in leukaemia cells.^14^ Further SAR studies showed that compound 13 (Fig. 1d) caused a high level of inhibition of RelA/p65-DNA binding in chronic lymphocytic leukaemia (CLL) cells.^12,14^ Other molecules, like CRL1101 and IT-901 (Fig. 1e and f), were also reported to block NF-κB subunits RelA/p65 and c-Rel^8,15^ but none of these compounds have been approved for use as anticancer agents.

Recently, proteolysis targeting chimeras (PROTACs) have emerged as a promising alternative strategy for selectively interfering with the activity of TFs like NF-κB. Unlike traditional small molecule inhibitors or antibodies, PROTACs recruit the ubiquitin-protease system to break down a specific protein of interest (POI), leading to target protein degradation (TPD). The PROTAC recognises the protein target *via* its POI ligand, while the E3 ligand located at the other end of the molecule will bring an E3 ligase proximate to the target substrate for consequent POI ubiquitination.^16,17^ This ubiquitinated protein is subsequently degraded in the proteasome.^16,17^ Due to their distinct mechanism of action, PROTACs can target non-druggable proteins that lack enzymatic activities, such as TFs, and they do not need to be maintained at high concentrations to induce their therapeutic effect,^18–20^ which can diminish on-target toxicity concerns.^18–20^ PROTACs also have the potential to re-sensitise cancer cells to chemotherapy due to their ability to degrade proteins associated with drug resistance.^21^ Considering these unique therapeutic characteristics, PROTAC technology has the potential to expand the library of drug candidates for clinical purposes. Indeed, PROTACs have already shown remarkable ability to promote anti-cancer activity.^18^

**Fig. 2:**
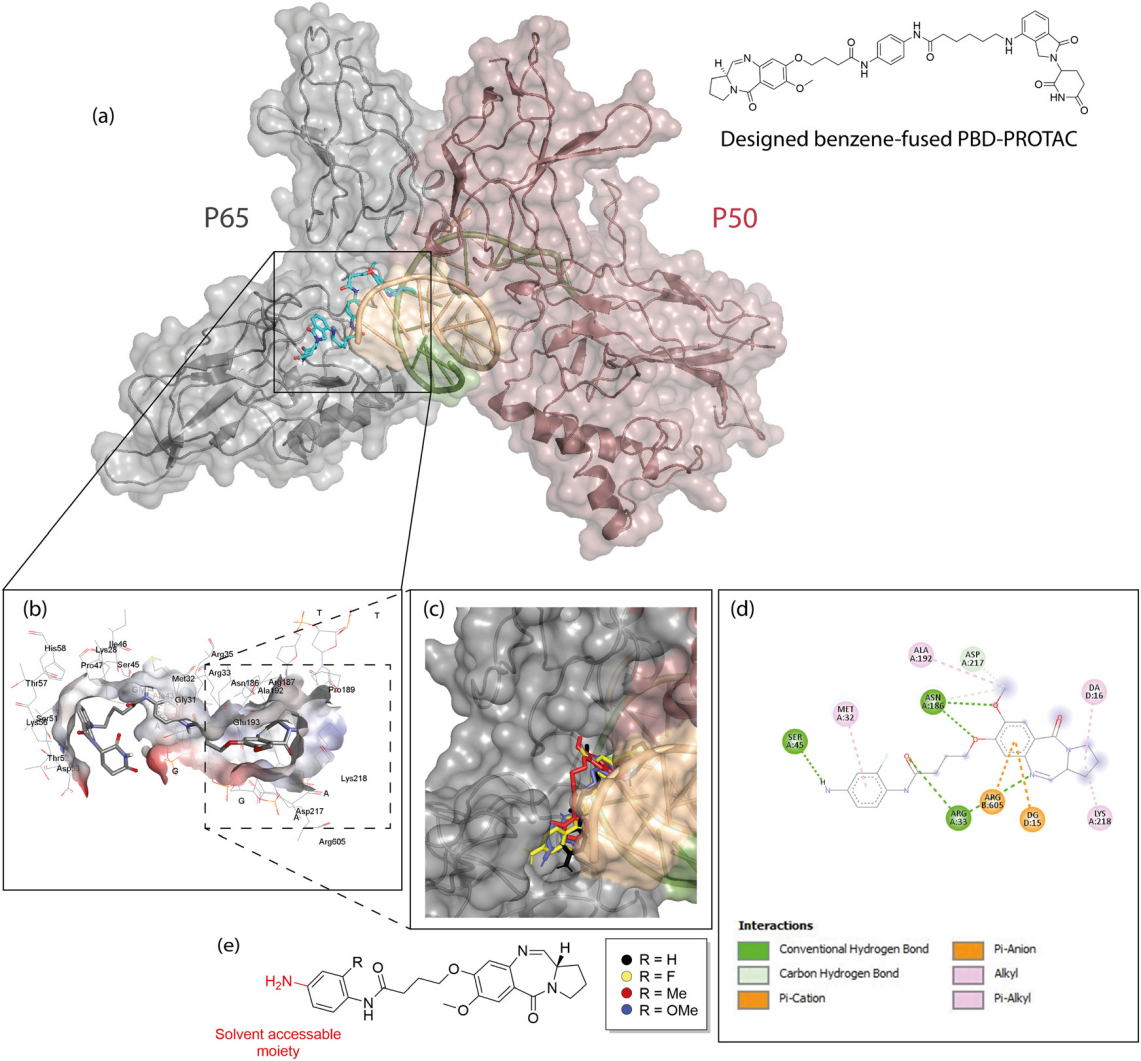
Docking of PBD-PROTAC with the RelA/p65-p50-DNA complex (PDB:1VKX). (a) Molecular docking of the designed PBD-PROTAC (15d); (b) zoomed in figure showing the 15d binding pose in the RelA/p65-DNA binding interface; (c) overlapped docking results of PBD controls within the RelA/p65-DNA interface; (d) the typical 2D ligand-interface binding projection of PBD ligands. Here fluorine-substituted PBD (JP-193-12) was selected as an example, while the dashed lines highlight the protein–ligand interactions. (e) Chemical structure of the docked PBD building blocks.

This project set out to develop PROTACs that can selectively degrade the NF-κB protein RelA/p65. The objective was to deplete this single NF-κB subunit, which is over-expressed in some cancer cells,^4,5^ whilst preserving the other components of the NF-κB signalling machinery. In so doing, we hoped to selectively target the tumour cells and diminish on-target toxicities in normal cells. Although other researchers have developed NF-κB targeting PROTACs,^22^ to date there are no reports of PROTACs that are able to selectively degrade a single NF-κB subunit. Although PBDs are well-characterised DNA interacting agents, more recently, C8-conjugated PBDs have been shown to also interact with proteins.^23^ We used PBD derivatives that were shown by *in silico* modelling to be able to bind to RelA/p65 (Fig. 2). The PBDs were then conjugated with cereblon (CRBN)-recruiting PROTAC building blocks *via* multi-step synthetic routes. These synthesised molecules were then biologically screened for their toxicity in cancer cells and non-malignant B- and T-lymphocytes. Subsequently, preliminary investigations were carried out to establish their mechanism of action.

## Results and discussion

### Design and synthesis of RelA/p65-targeting PROTACs

To design NF-κB targeting PROTACs, C8-conjugated PBD molecules were selected as RelA/p65-targeting ligands as these C8-phenyl linked short PBDs had been previously shown to inhibit the activity of the canonical RelA/p65 NF-κB subunit, implying their potential as NF-κB-targeting POI ligands.^12^ The initial concept was to produce RelA/p65-targeting PROTACs that were linked to CRBN-based building blocks. For a PROTAC to work, it's two functional components must be connected by a linker. One part of the molecule selectively binds the target protein, whilst the second part of the molecule recruits a cellular enzyme, an E3 ligase. This enables the PROTAC to recruit an E3 ligase enzyme to come into close contact with the target protein, enabling the protein to be marked for degradation in the cellular proteasome. Previous research on early-stage PROTAC design used a linear chain of alkyl units as a linker, which allowed the length of the linker to be modified (typically 5–11 carbon chain).^17,22,24,25^ Here, we selected a 5-carbon aliphatic linker for our preliminary study as molecular modelling suggested that this would allow the CRBN-ligand (lenalidomide) to be bioavailable once the POI had been captured. The designed structure and the docking simulation are shown in Fig. 2 (PBD: 1VKX). It was noted that the PBD moiety was stabilized at the interface between the DNA segment and RelA/p65 NF-κB subunit, while the CRBN ligand domain protruded outside the structure, suggesting that it would remain accessible for CRBN binding. Given the substantial interactions with RelA/p65, we hypothesized that this PBD molecule may have sufficient RelA/p65 binding to serve as a POI ligand.

**Scheme 1:**
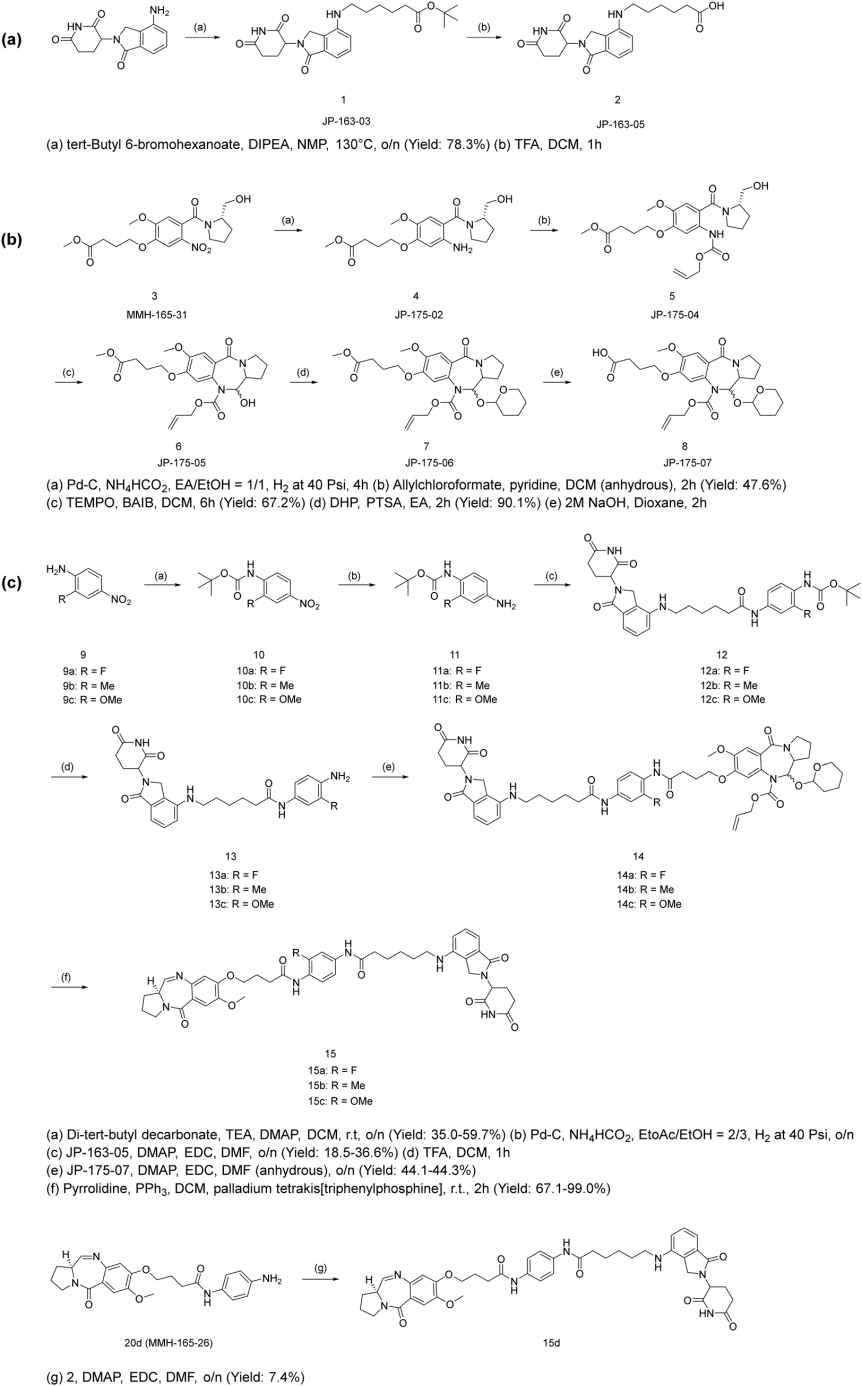
Synthesis of (a) lenalidomide-based CRBN building block JP-163-05; (b) PBD core; (c) PBD-based PROTACs.^12,14^

**Scheme 2:**
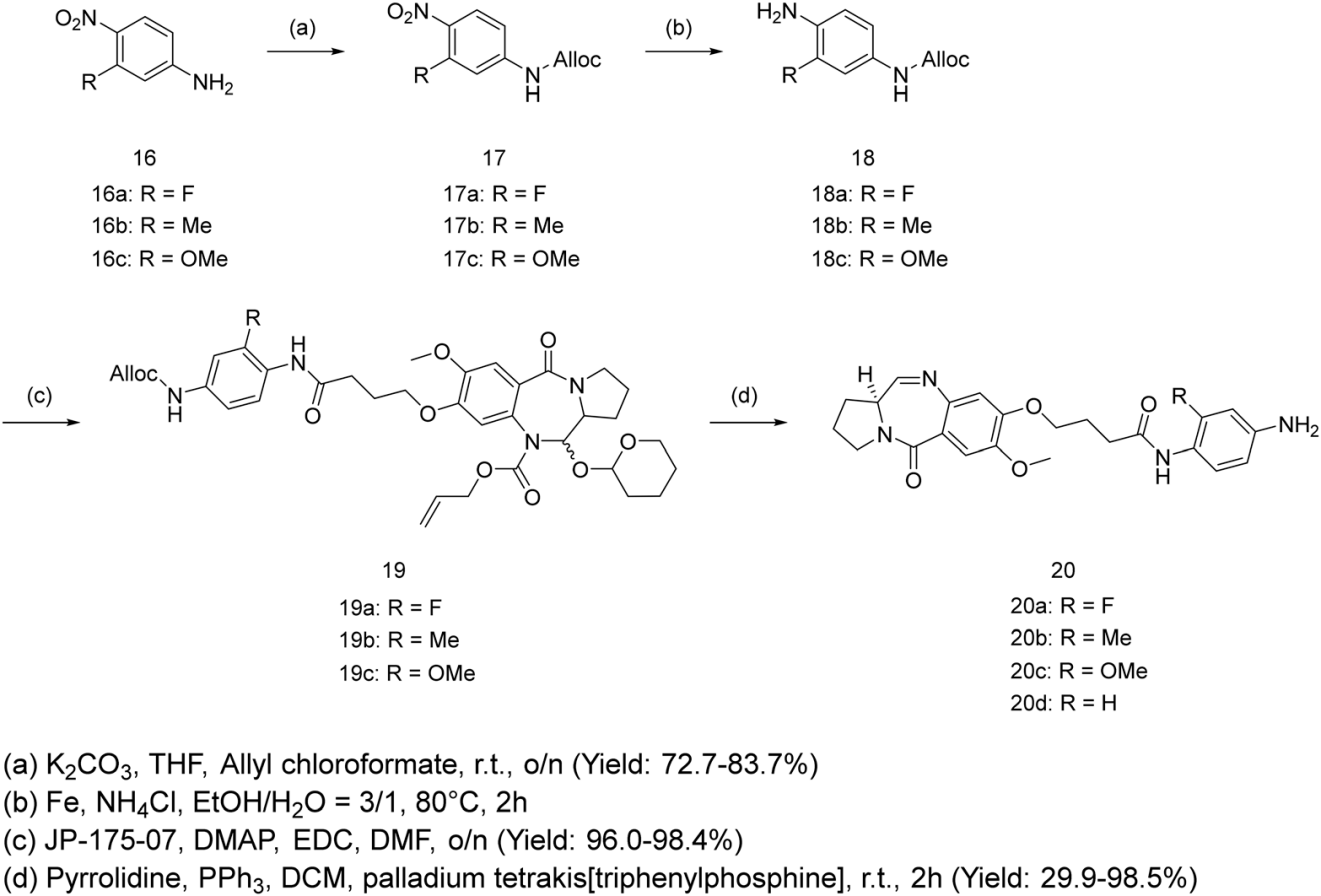
Synthesis of PBD controls.

The expanded figure shows that the POI ligand binds in the RelA/p65 domain between Glu193 and Lys218 (Fig. 2b). Further investigation of the short C8-phenyl linked short PBD molecules suggested a similar binding pattern where the PBD core resides inside, and the aniline moiety is relatively solvent-exposed (Fig. 2c and d). To synthesize the molecules, the synthetic scheme of the PROTAC building block was based on the conditions reported by Qiu *et al.* (Scheme 1a).^26^ The PBD core was then synthesized as RelA/p65-targeting ligands as shown in Scheme 1b.^12,14^ PBD-based PROTACs were generated as shown in Scheme 1c. Di-*tert*-butyl decarbonate was used to install a Boc-protecting group towards the exposed amine of the starting material 9. The product 10 underwent nitro reduction catalysed by Pd–C, while the reduced amine was then attached to the PROTAC building block 2 to generate 12. In the next step, TFA was used to deprotect the Boc group and the product was thus coupled with the PBD core *via* the EDC/DMAP mediated amide coupling reaction. Finally, the THP and alloc protecting groups were simultaneously removed *via* pyrrolidine and tetrakis(triphenylphosphine)palladium(0), to obtain the final series 15a–15c. 15d was directly synthesized *via* the EDC/DMAP mediated amide coupling reaction. For the synthesis of PBD controls 20a–20d, the aromatic-substituted amine was initially conjugated to the PBD core *via* an amide coupling reaction, and the products were obtained by removing THP and alloc protecting groups (Scheme 2). The cytotoxicity of the synthesised PROTACs was compared with the individual constituent PBD controls.

### 15d exhibits cytotoxicity in MEC-1 cells

To evaluate the therapeutic potential of the PBD-PROTACs and their constituent PBD building blocks, all the samples were tested in the chronic lymphocytic leukaemia (CLL) cell line, MEC-1. Cells were exposed to PROTACs or their PBD building blocks for 48 h, and drug-induced apoptosis was quantified using annexin V and 7-AAD labelling using flow cytometry. Given the known DNA-binding characteristics of PBDs, we used a FRET-melting assay to compare the ability of the PROTAC molecules and their constituent PBD molecules to interact with DNA. All the compounds were serially diluted (10 μM) and then added to annealed FAM-TAMRA labelled AT-rich single stranded hairpin DNA. DNA Engine Opticom was used to determine the DNA melting characteristics. The sample was initially incubated at 30 °C for 3 h and then gradually increased to 100 °C. The FAM-TAMRA fluorescent signal was detected at 0.5 °C intervals and the mean melting point was determined using GraphPad Prism software. The melting point difference between each compound and naked ssDNA (Δ*T*_m_) was calculated for comparison.

**Fig. 3:**
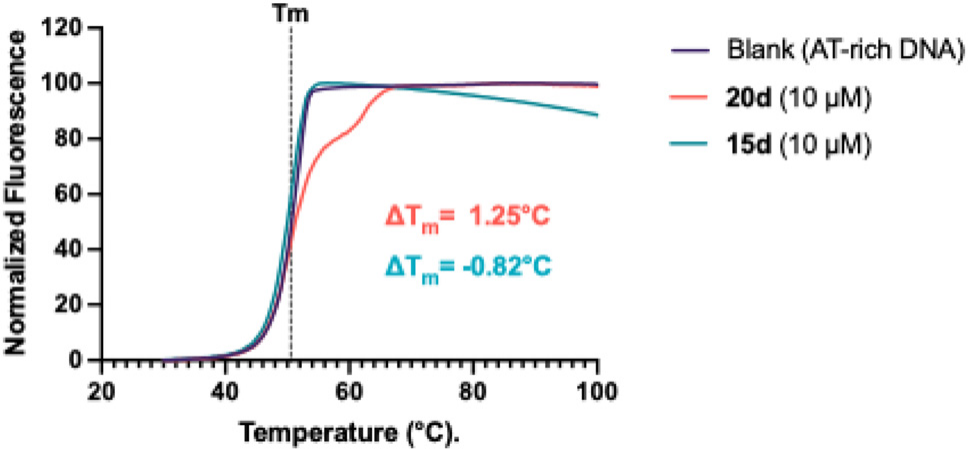
FRET melting assay results reveal that the PROTAC (15d) does not significantly interact with DNA. Each compound was mixed with an AT-rich DNA sequence at 10 μM. 15d did not increase the melting temperature suggesting that it does not interact with DNA. In contrast, the PBD building block (20d) caused a marked increase in DNA melting temperature, confirming its ability to bind to and stabilise DNA.

The screening results are presented in Table 1, and the dose–response curves are attached in Fig. S12.† Compound 15d showed the highest anti-tumour effects in MEC-1 cells. The result of the FRET-melting assay is shown in Fig. 3. 50 equivalents of PBD (10 μM) induced the shift of melting point of the DNA by Δ*T*_m_ of 1.25 °C, whereas the same equivalence of PROTAC compound 15d showed almost no change in Δ*T*_m_, suggesting that these compounds do not interact with DNA. Consequently, the high toxicity of 15d in cancer cell lines and primary CLL cells is unlikely to be attributable to the ability of the PROTAC to bind to DNA. These data suggest a distinct mechanism of action of the PROTAC when compared to its PBD building block.

**Table 1:** Cytotoxic activities in the MEC-1 cell line and DNA-binding characteristics of the PBD-based PROTACs and their PBD building blocks

PROTAC	MW	LC_50_ (μM)	Δ*T*_m_ (°C)	PBD building blocks	MW	LC_50_ (μM)	Δ*T*_m_ (°C)
15d (JP-163-16)	777.88	0.14 ± 0.02	−0.82	20d (MMH-165-26)	422.49	0.31 ± 0.14	1.25
15a	795.87	0.81 ± 1.47	1.05	20a	440.48	0.03 ± 0.29	2.17
15b	791.91	0.84 ± 0.48	0.73	20b	436.51	1.18	−0.06
15c	807.91	>1000	1.27	20c	452.51	1.31 ± 0.74	0.70

### 15d causes RelA/p65 degradation in a proteasome-dependent manner

**Fig. 4:**
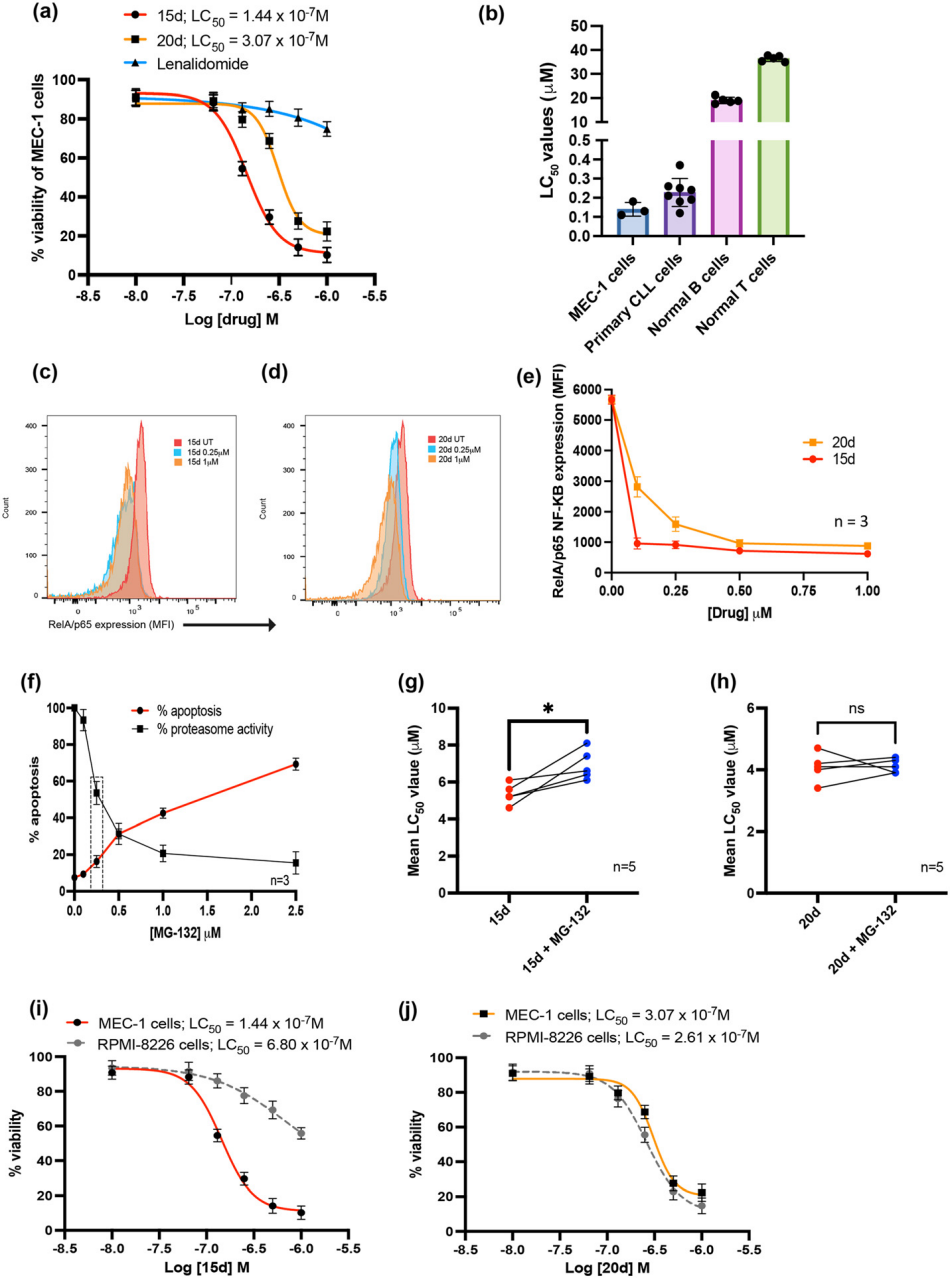
(a) Overlaid dose–response curves for 15d, 20d, and lenalidomide in the MEC-1 cell line. Dose–response curves were generated using annexin V/7-AAD data following 48 h of exposure to each compound. The LD_50_ values were interpolated from each individual dose–response curve using GraphPad Prism 10, with all experiments performed in triplicate. (b) Shows the relative cytotoxic effect of 15d in MEC-1 cells, primary CLL cells and normal B- and T-lymphocytes. Normal lymphocytes were more than two logs less sensitive to the effects of 15d when compared with malignant B cells. RelA/p65 expression was significantly reduced in MEC-1 cells treated for 24 h with (c) 15d and (d) 20d. (e) In contrast to 20d, 15d did not show a dose-dependent reduction in RelA/p65, which suggests an event-driven mechanism of action consistent with other PROTACs. Furthermore, co-treatment with the proteasome inhibitor, MG-132, demonstrated a proteasome-dependent mechanism of action. (f) The cytotoxic and proteasome inhibitory effects of MG-132 were evaluated in MEC-1 cells and a concentration of 0.18 μM was chosen for the subsequent combination studies. (g) 15d shows a proteasome dependent mechanism of action. (h) In contrast, the PBD building block, 20d, showed a proteasome-independent mechanism of action. (i) The cytotoxic effects of 15d were shown to be dependent on CRBN expression by the five-fold reduction in potency in the CRBN deficient myeloma cell line, RPMI-8226. (j) In contrast, the PBD building block, 20d, showed similar potency in RPMI-8226 cells. All LC_50_ values were interpolated from individual dose–response curves using GraphPad Prism 10. Results are shown as the mean of five independent experiments carried out in duplicate. Statistical significance was determined using paired *t*-tests * *p* < 0.05.

To further explore the relative potency and mechanism of action of 15d, the lead PROTAC molecule was tested in MEC-1 cells, primary CLL cells derived from patients (*n* = 8) and normal B-and T-lymphocytes (*n* = 5). Cells were exposed to 15d, its PBD building block 20d, or lenalidomide for 48 h and drug-induced apoptosis was quantified using the same assay as described above. 15d and 20d showed potent anti-tumour effects in MEC-1 cells, while lenalidomide had a negligible impact on MEC-1 cell viability (Fig. 4a). Primary CLL cells were also sensitive to the apoptotic effects of 15d. In contrast, normal B- and T-lymphocytes were more than two logs less sensitive to the effects of the PROTAC (Fig. 4b). In parallel experiments, RelA/p65 expression was measured in MEC-1 cells treated with 15d or 20d. Fig. 4c–e show that both agents induced a marked reduction in RelA/p65 expression. However, the dose–response patterns of the two molecules were different. 20d induced a dose-dependent reduction in RelA/p65 expression. In contrast, 15d showed a similar reduction in RelA/p65 with all the concentrations tested. This supports the concept that 15d may have catalytic properties, which enable the recycling of PROTAC molecules after the target protein is degraded. In contrast, the PBD, 20d, demonstrated an occupancy-driven mechanism of action; a more obvious concentration-dependent reduction in RelA/p65 was observed in MEC-1 cells treated with 20d. Given its known DNA-binding activity, it seems likely that dose-dependent minor groove binding contributes to the cytotoxicity of 20d, which could, in turn, result in higher levels of competitive blockade of RelA/p65 DNA binding sites.^12,27^ In contrast, the PROTAC molecule, 15d, appeared to induce an event-driven pharmacology consistent with POI degradation. Interestingly, although the PROTAC induced similar levels of RelA/p65 reduction at both 0.25 μM and 1 μM, the higher concentration of 15d induced a stronger anti-tumour effect. This unique pattern could be caused by the ‘hook effect’ frequently observed with PROTAC compounds.^20,27,28^

To confirm the proteasome dependency on the cytotoxic effects of 15d in MEC-1 cells, cells were co-treated with the proteasome inhibitor MG-132. We initially established the cytotoxic and proteasome inhibitory effects of MG-132 in MEC-1 cells (Fig. 4f). MEC-1 cells were treated with increasing concentrations of the proteasome inhibitor, MG-132 (0.1–2.5 μM) for 48 h. Aliquots of cells were first assessed for their apoptotic response to MG-132 using annexin V and 7-AAD labelling. In parallel, proteasome activity was evaluated using a proteasome activity assay kit (Abcam). The kit uses an AMC-tagged peptide substrate (proteasome substrate (Succ-LLVY-AMC in DMSO), which releases free, highly fluorescent AMC (Ex/Em 350/440 nm) in the presence of proteolytic activity. Due to the high sensitivity of MEC-1 cells to the effects of MG-132, a concentration of 0.18 μM MG-132 was used in combination with 15d or 20d.

This concentration of MG-132 reduced cellular proteasome activity by approximately 40–50% without inducing significant apoptosis. Each experiment was repeated five times in duplicate, and the mean LC_50_ values were calculated for each experiment. Subsequently the matched LC_50_ values (± the addition of MG-132) were plotted and the difference between the LC_50_ values was determined using the paired *t*-test (Fig. 4g and h). The results indicate that blocking the proteasome significantly repressed the cytotoxicity of 15d (*p* < 0.05), while there was no significant difference in LC_50_ values when MG-132 was co-administered with the PBD 20d. This provided further evidence that the anti-tumour effect of 15d is caused, at least in part, by proteasome-dependent degradation of RelA/p65. However, it should be noted that 15d was not able to entirely deplete the cellular expression of this molecule. Although molecular modelling showed excellent binding characteristics of the PBD PROTAC with RelA/p65, it may not result in the optimal degradation of RelA/p65.^12^ The scale of target protein degradation is reliant on the stability and positive cooperativity of the ternary complex, so a ligand bearing an inferior binding affinity could still induce effective protein degradation with considerable selectivity.^19,20^ Issues like charged repulsion between the E3 ligase and POI or steric clashes induced by unfavourable PROTAC conformation are more likely to be the cause of the incomplete RelA/p65 degradation.^7,18,19^ Although we did not directly explore ternary complex formation *i.e.*, the binding of the PROTAC to both the protein of interest (RelA/p65) and the target E3 ligase (CRBN), we did examine the cytotoxic effects of 15d in the multiple myeloma cell line, RPMI-8226. These cells have very low CRBN protein expression and are consequently resistant to lenalidomide.^29^ Using these cells we were able to confirm that the mechanism of action of 15d is, at least in part, dependent on CRBN; the PROTAC showed a five-fold reduction in efficacy in RPMI-8226 cells (Fig. 4i). In contrast, the PBD building block 20d showed similar cytotoxicity in RPMI-8226 cells and MEC-1 cells (Fig. 4j) confirming that its cytotoxic effects were independent of CRBN.

Previous research indicated that PBD molecules fused with benzofuran and pyrrole terminal were selective for RelA/p65 inhibition. In contrast, benzene-fused short PBDs caused very limited RelA/p65 perturbance, but with significant effects on other NF-κB subunits.^12^ Consequently, the effect of 15d was evaluated on other NF-κB subunits (RelB and cRel) as described below.

### 15d selectively degrades the NF-κB RelA/p65 subunit

**Fig. 5:**
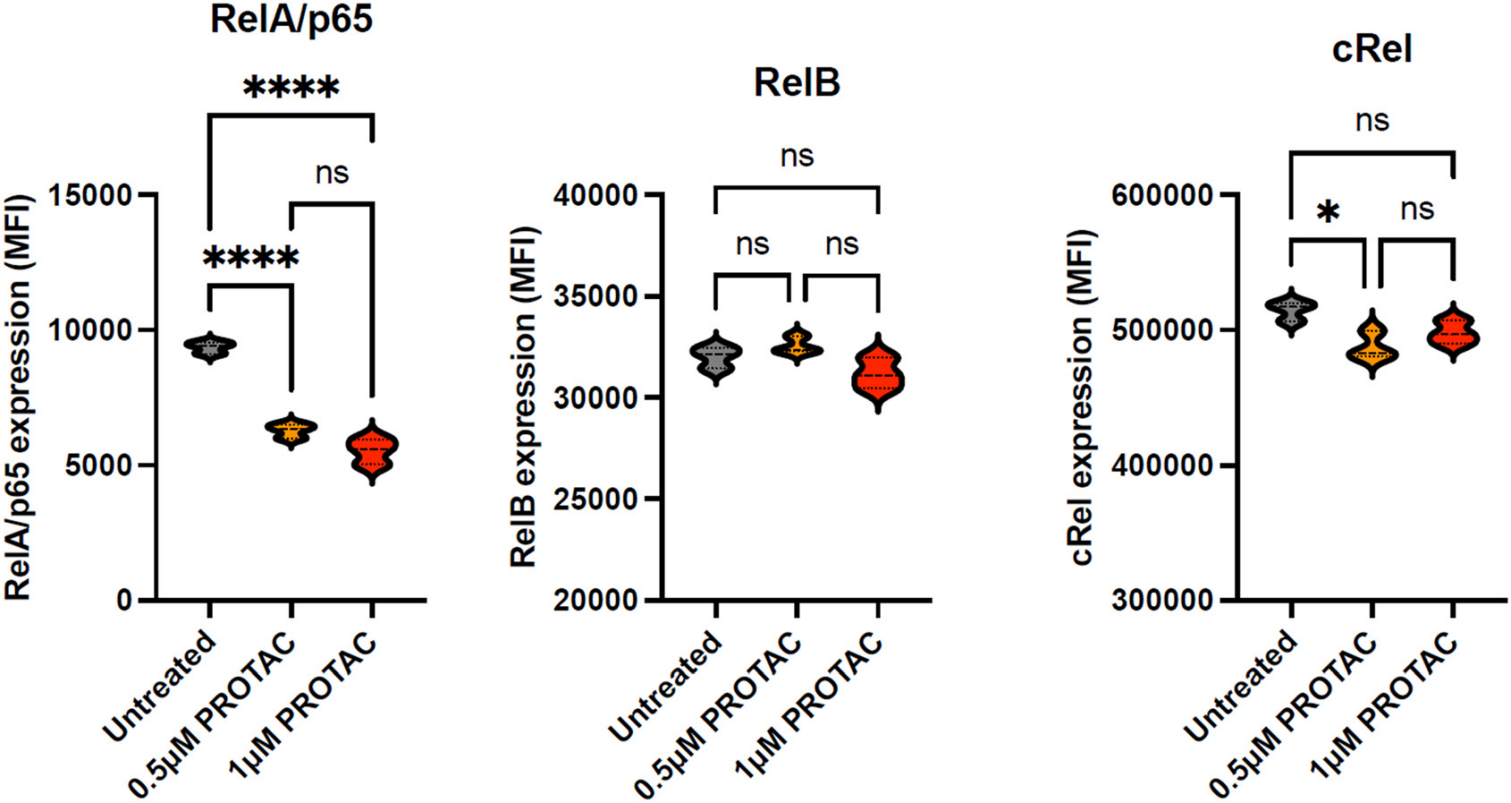
Comparison the effect of 15d (PROTAC) on the expression of three NF-κB subunits RelA/p65, RelB and cRel. Each subunit was quantified using fluorescence-labelled antibodies; all experiments were performed three times in duplicate and data are presented as violin plots. Statistical significance was determined using the Kruskal–Wallis test with Dunn's multiple comparison *post hoc* correction. * *p* < 0.05, ** *p* < 0.01, *** *p* < 0.001, **** *p* < 0.0001.

To ascertain the selectivity of 15d for the degradation of the RelA/p65 NF-κB subunit, MEC-1 cells were treated for 24 hours with a range of concentrations of 15d (0–1 μM). Cells were then harvested, fixed and permeabilised and labelled with fluorescence-labelled antibodies against the NF-κB subunits p65 (APC), RelB (Corallite 488) and cRel (PE). Protein expression was quantified using a CytoFLEX LX flow cytometer. Fig. 5 shows that 15d induced a marked reduction in RelA/p65 expression at 0.5 μM and 1 μM. In contrast, no significant change in RelB was observed at the same concentrations. Although a small but significant reduction in cRel was noted at 0.5 μM, this was not replicated at 1 μM. This suggests that 15d selectivity depletes RelA/p65, which adds to its promising characteristics as a lead PROTAC compound. To our knowledge, 15d represents the first example of a RelA/p65 selective PROTAC that does not substantially impact RelB or cRel.

### Effects of PROTAC 15d on MDA-MB-231 cell viability

**Fig. 6:**
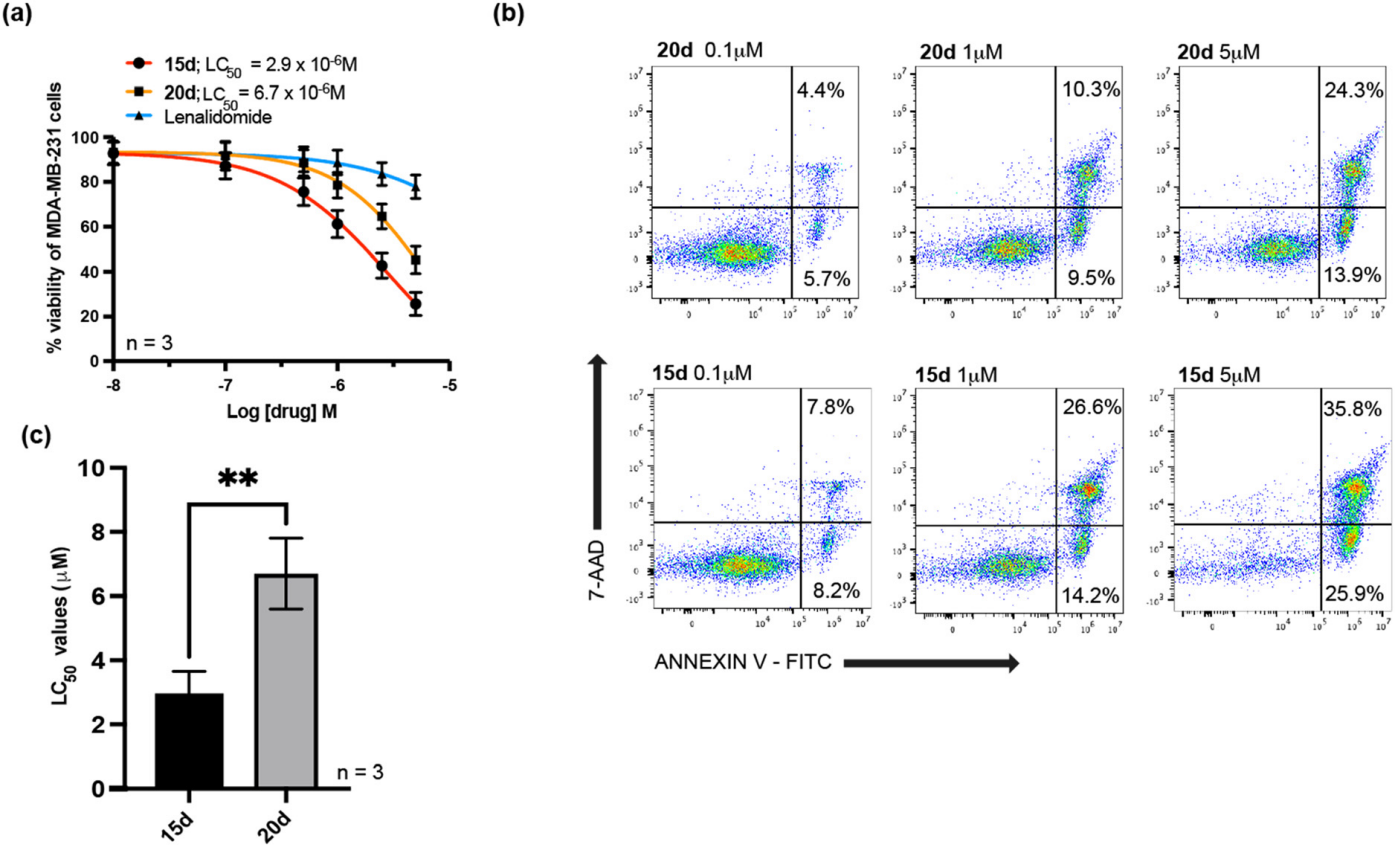
Evaluation of 15d in MDA-MB-231 cells. (a) Dose–response curves for 15d, 20d, and lenalidomide in MDA-MB-231 cells. Cells were treated with a range of concentrations of the PROTAC (15d) and the individual constituent molecules (20d and lenalidomide). Dose–response curves were generated using annexin V/7-AAD data following 48 h of exposure to each compound. The LC_50_ values were interpolated from each individual dose–response curve using GraphPad Prism 10. All experiments were performed in triplicate. (b) Shows an example of the gating strategy used to identify viable and apoptotic MDA-MB-231 cells. The percentage of viable cells was defined by cells being annexin-V and 7AAD negative. (c) The mean LC_50_ values (+SD) for 15d and 20d are shown for three independent experiments carried out in triplicate. 15d was significantly more cytotoxic than 20d, ** *p* < 0.001.

We next investigated the potency of 15d in the triple-negative breast cancer cell line (MDA-MB-231). This cell line was selected as it over expresses RelA/p65 and represents a cancer type with significant clinical unmet need.^30,31^ As shown in Fig. 6a, 15d was more potent than its constituent molecules, the POI targeting PBD, 20d and the CRBN E3 ligase ligand, lenalidomide. The cells were treated with serial dilutions of each compound and then incubated for 48 hours. FITC annexin V and 7-AAD labelling was used to evaluate cell viability using flow cytometry. In keeping with our findings in MEC-1 and primary CLL cells, 15d and MMH-165-26 both demonstrated high tumour suppressive effects in cancer cells with LC_50_ values in the low micromolar range, and 15d was significantly more potent than its PBD building block, 20d (Fig. 6c). The CRBN ligand, lenalidomide, showed low cytotoxicity even at the highest concentration tested.

### 15d promotes the degradation of RelA/p65 in MDA-MB-231 cells in a proteasome-dependent manner

**Fig. 7:**
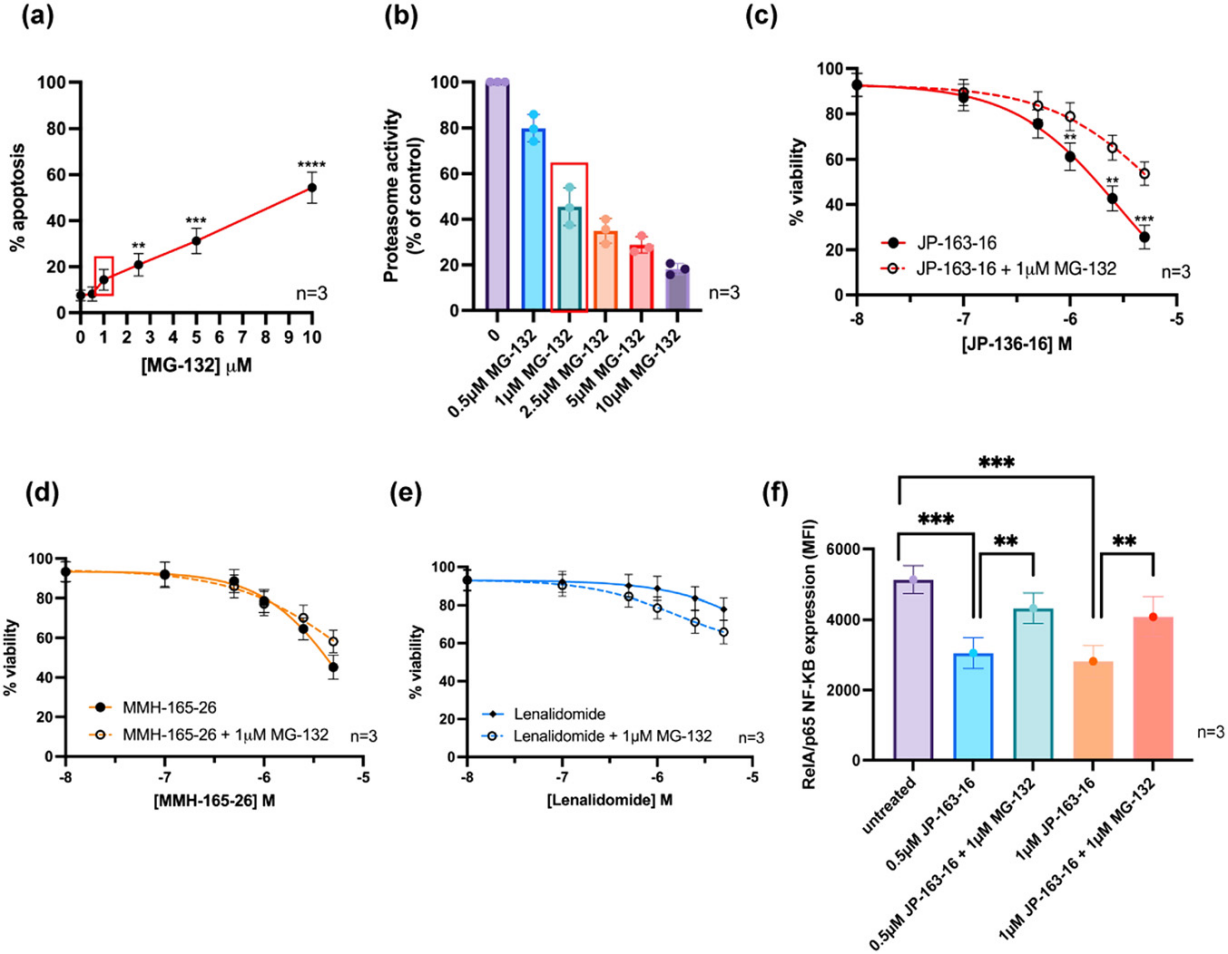
The effects of the proteasome inhibitor, MG-132, in the MDA-MB-231 cell line. (a) MG-132 induced dose-dependent cytotoxicity which was (b) associated with a dose-inhibition of proteasomal activity. A dose of 1 μM MG-132 did cause a significant increase in cytotoxicity but induced a >50% reduction in proteasome activity. All experiments were performed in triplicate and statistical significance was determined using the Kruskal–Wallis test with Dunn's multiple comparison *post hoc*. ** *p* < 0.01, *** *p* < 0.001, **** *p* < 0.0001. (c) Overlaid dose–response curves of MDA-MB-231 cells treated with 15d with and without the addition of the proteasome inhibitor, MG-132. (d) 20d and (e) lenalidomide. The cytotoxic effect of 15d was significantly reduced by co-treatment with 1 μM MG-132. This was not the case for 20d or lenalidomide. All experiments were performed in triplicate and statistical significance was determined using the Kruskal–Wallis test with Dunn's multiple comparison *post hoc* correction. *** *p* < 0.001. (f) 15d mediated depletion of RelA/p65 expression in MDA-MB-231 cells was dependent on proteasome activity. Experiments were performed in triplicate and statistical significance was determined using the Kruskal–Wallis test with Dunn's multiple comparison *post hoc* correction. ** *p* < 0.01, *** *p* < 0.001.

Next, to confirm the PROTAC mechanism of action of 15d, MDA-MB-231 cells were co-treated with the proteasome inhibitor, MG-132, to evaluate whether this altered the tumour suppressive effects.^32^ MG-132 was much less cytotoxic in MDA-MB-231 cells when compared with MEC-1 cells, and again a concentration of MG-132 was selected that did not have significant cytotoxicity but inhibited proteasome activity by approximately 50% (Fig. 7a).^33^ Analysis of the cytotoxic dose–response curves and the impact of MG-132 on proteasomal activity in MDA-MB-231 cells revealed that 1 μM MG-132 caused >50% reduction in proteasome activity in MDA-MB-231 cells without inducing a significant reduction in cell viability when compared with the untreated controls (Fig. 7a and b). Based on the evaluation of the effects of MG-132 in MDA-MB-231 cells, cells were then treated with 15d, 20d and lenalidomide, with and without the addition of 1 μM MG-132.

Co-treatment with MG-132 significantly reduced the cytotoxic effect of 15d, which indicated a proteasome-dependent mechanism of action (Fig. 7c). In contrast, MG-132 did not significantly alter the cytotoxicity of 20d, implying again that the mechanism of action of this PBD compound was independent of proteasomal function (Fig. 7d). In the case of lenalidomide, blocking proteasome activity increased its cytotoxicity, but this was not statistically significant (Fig. 7e).^33^ This result indicated that the mechanism of the PBD was distinct from that of 15d as it was not dependent on proteasomal activity. As a C8-linked short PBD, 20d can bind into the minor groove of DNA and form a covalent bond with guanine molecules. This may contribute to its effect on NF-κB as it facilitates sequence-selective binding at promoter regions containing the guanine-rich NF-κB binding motifs, thereby disrupting NF-κB signal transduction.^11,12,34^ In this case, 20d may retain its anti-tumour activity after the blockade of proteasome function.

As for the E3 ligase ligand, lenalidomide, used to develop 15d, it did not significantly induce cell death as a single agent, but co-administration of MG-132 mildly improved tumour suppression. This could be caused by the additive impact of combination treatment as lenalidomide promotes immune cell activation that might slightly improve tumour sensitivity to other agents bearing distinct mechanisms, like MG-132.^35–37^ Subsequently, the ability of 15d to reduce RelA/p65 expression in MDA-MB-231 cells was evaluated using flow cytometry. Cells were treated with increased concentration of 15d for 24 h, and then they were harvested and permeabilised followed by labelling with an APC-labelled RelA/p65 antibody. A significant reduction in RelA/p65 expression was observed after treating with 0.5 μM and 1 μM 15d, which when co-administrated with 1 μM MG-132 reversed this effect, consistent with a proteasome-dependent mechanism of action (Fig. 4f). It was noted that 0.5 μM administration of 15d caused a similar depletion of RelA/p65 to that achieved with 1 μM. Due to the bifunctional characteristic of PROTAC molecules, high intracellular levels of PROTAC may lead to saturated binding of its relative binary complexes, which competitively restricts the formation of the effective ternary complex required for target protein degradation.^20,27,28,38^ It is possible that 15d saturated the binding sites of RelA/p65 and/or CRBN in MDA-MB-231 cells at 1 μM, which may restrict the formation of the POI–PROTAC–E3 complex thereby limiting the capacity for RelA/p65 degradation.^20,28^ In summary, 15d induced cytotoxicity in MDA-MB-231 cells and promoted the degradation of RelA/p65 in a proteasome-dependent manner.

## Conclusions

Targeting the NF-κB signalling pathway has long been an appealing therapeutic strategy due to its role in regulating a range of cellular processes. The aberrant protein expression of one or more NF-κB subunits often results in increased NF-κB signalling, which is associated with pathogenic effects such as tumour proliferation, angiogenesis, and drug resistance. Here, we report the first prototype PROTAC capable of selectively degrading the NF-κB subunit RelA/p65. The POI targeting ligand was a C8-linked short PBD; simulated docking experiments showed strong binding to RelA/p65 protein in a region where the protein was predicted to interact with NF-κB DNA motifs. This supported its use as a selective ligand for targeting RelA/p65 protein if incorporated into a bifunctional PROTAC molecule. Consequently, PBD molecules were conjugated with a lenalidomide-based building block *via* an amide coupling reaction. A series of PBD PROTACs were synthesized, and all final products were purified either by flash column chromatography or preparative chromatography. Biological screening indicated that the lead compound, 15d, showed potency in the TNBC breast cancer cell line, MDA-MB-231 (LC_50_ = 2.9 μM), CLL cell line MEC-1 (LC_50_ = 0.14 μM) and primary CLL B cells derived from eight patients (LC_50_ = 0.23 μM). In all cases, this was associated with the selective depletion of RelA/p65 in a proteasome-dependent manner. It is noteworthy that the cytotoxicity of 15d was two logs lower in non-malignant B- and T-lymphocytes derived from healthy volunteers and was five-fold lower in RPMI-8226 cells, which possess very low levels of CRBN protein expression.

The proteasome-recruitment mechanism of 15d was confirmed by the reversal of RelA/p65 depletion when cells were co-treated with the proteasome inhibitor, MG-132. Furthermore, a FRET-melting assay confirmed that this compound did not interact with DNA, which was in contrast with the strong DNA interaction shown by its constituent PBD, 20d. Hence, the cytotoxicity of 15d appeared to be predominately driven by proteasome-dependent RelA/p65 degradation. Further screening of the small library and comparison with simulated modelling results implied that PROTAC potency may be partially related to the binding affinity of the POI ligand, while it was also reliant on ternary complex formation as demonstrated by the reduced toxicity and RelA/p65 degradation in the presence of MG-132 and reduced cytotoxicity in RPMI-8226 cells which have low cereblon expression. It is worth noting that 15d was not able to abolish RelA/p65 expression, which suggests that further PROTAC optimisation may be possible. Despite the incomplete target degradation observed in our studies, the work presented here demonstrates, for the first time, that it is possible to produce a PROTAC with the ability to preferentially degrade a single NF-κB subunit, RelA/p65. As such, this may represent an important step towards unlocking the potential of NF-κB as a therapeutic target. In particular, the generation of a PROTAC with the ability to selectively degrade RelA/p65 may open the door to more effective and better tolerated treatments for human pathologies that are associated with RelA/p65 overexpression, including a range of cancers and autoimmune disorders.

## Experimental section

### Molecular docking

The protein data file was accessed from the PBD data bank (p65 protein data: 1VKX); all compound ligands were prepared and generated *via* either Chem 3D or Avogadro. All the compounds were calculated and performed with energy minimization *via* MMFF94. For p65-targeting ligands, molecular docking was performed by the Vina molecular docking programme using VEGA-ZZ modelling software. The parameters were set as *X*: −11.3, *Y*: 40.4, *Z*: 76.7; box: 30, 30, 30; exhaustiveness: 20; binding mode: 16. All docking results were visualised *via* Discovery Studio. The result was visualised *via* PyMOL.

### Chemistry materials and methods

All synthetic chemicals, building blocks, and solvents were purchased from Fluorochem, Sigma-Aldrich, and Thermo-Fisher Scientific. All reactions monitored *via* thin-layer chromatography (TLC) were performed by using Supelco TLC silica gel 60 F_254_ aluminium plates. The TLC plates were visualised using a UV lamp at 254 nm. Purification through flash column chromatography was performed in a glass column with silica gel as the stationary phase (230–400 mesh, 60 Å). Preparative HPLC was also used for purifying some products. An Agilent 1260 Preparative LC system was applied using H_2_O (solvent A) and acetonitrile (solvent B) as the mobile phase with a Monolithic C_18_ 50 × 4.6 mm LC column (Phenomenex) as the stationary phase. Methods A, B, C, and D were used for purification (flow rate: 20 ml min^−1^). Formic acid was added (0.1%) into both solvent A and B to maintain an acidic mobile phase condition.

### Method A

The gradient was initially kept at 60% solvent A and 40% solvent B, while it was ramped up to 60% B over 2 min. Solvent B was then increased to 70% over 2 min, which was further ramped up to 80% over 2.5 min. The solvent reached 90% B over 0.5 min and kept for 2.5 min, which was then returned to 40% B over 1.5 min.

### Method B

The gradient was initially started from 90% A and 10% B and kept for 1 min, and then ramped up to 20% B over 1.5 min. B was then increased to 30% over 2 min, followed by ramping up to 40% B over 2 min. Subsequently, B was raised to 70% within 0.5 min, and then B was increased to 85% over 1 min, and then ramped up to 90% over 1 min. The solvent was finally returned to 10% B over 1 min.

### Method C

The gradient was initially started from 90% A with 10% B that is kept for 1 min, and solvent B was increased to 30% over 1.5 min. B was subsequently increased to 50% over 3.5 min, and then raised to 90% over 2 min and kept for 1 min. The gradient was finally reduced to 10% B over 1 min.

### Method D

The gradient was initially started from 90% A with 10% B that is kept for 1 min, and solvent B was increased to 50% over 5 min. The gradient was subsequently raised to 90% B over 2 min, and it was kept for 1 min. Solvent B was finally returned to 10% over 1 min.

High-performance liquid chromatography-tandem mass spectrometry (LCMS) was applied for monitoring reaction and characterizing products. The product analysis was carried out using an Agilent 1260 separating system using H_2_O (solvent A) and acetonitrile (solvent B) as the mobile phase, while a monolithic C_18_ 50 × 4.6 mm LC column (Phenomenex) worked as the stationary phase. Method E (10 min) and method F (5 min) were used for analysis (flow rate: 0.5 mL min^−1^; inject volume: 200 μL), while samples were split and passed through an Agilent 6120 quadrupole mass spectrometer. Formic acid was added (0.1%) into both solvent A and B to maintain an acid mobile phase condition.

### Method E (10 min run)

Solvent A (95%) with solvent B (5%) was maintained for 2 min, and then ramped up to 50% solvent B in 3 min. The gradient was retained for 1 min and then solvent B was increased to 95% in 1.5 min. Solvent B was finally returned to 5% in 1.5 min and maintained for 1 min.

### Method F (5 min run)

Solvent A (95%) with solvent B (5%) was ramped up to 90% in 3 min, while solvent B was then ramped up to 95% within 0.5 min. The solvent gradient was kept for 1 min, and then solvent B was reduced to 5% within 0.5 min.

#### *tert*-Butyl 6-((2-(2,6-dioxopiperidin-3-yl)-1-oxoisoindolin-4-yl)amino)hexanoate (1, **JP-163-03**)

Lenalidomide (0.800 g, 3.09 mmol, 1 eq.) was dissolved in NMP (3 mL), added with *tert*-butyl 6-bromo hexanoate (1.010 g, 4.02 mmol, 1.3 eq.) and DIPEA (1.62 mL, 9.27 mmol, 3 eq.). The mixture was stirred at 130 °C. After the reaction was finished, the mixture was quenched in 50× volume of water, and it was extracted with 3 × 30 mL EA. The organic phase was collected and washed with 3 × 30 mL water, brine, and dried over MgSO_4_. The crude was purified in C_18_ preparative column chromatography (method A), and 1.04 g product was obtained after evaporation (yield: 78.3%).^25^^1^H NMR (400 MHz, DMSO-d_6_): *δ* 1.38 (s, 11H), *δ* 1.50–1.61 (m, 4H), *δ* 2.02–2.08 (m, 1H), *δ* 2.19 (t, 2H, *J* = 7.24 Hz), *δ* 2.24–2.38 (m, 1H), *δ* 2.60–2.64 (m, 1H), *δ* 2.88–2.97 (m, 1H), *δ* 3.09–3.14 (q, 2H, *J* = 6.37 Hz), *δ* 4.10–4.25 (q, 2H, *J* = 19.01 Hz), *δ* 5.09–5.13 (dd, 1H, *J* = 5.03, 13.22 Hz), *δ* 5.52–5.55 (t, 1H, *J* = 5.24 Hz), *δ* 6.73–6.75 (d, 1H, *J* = 8.03 Hz), *δ* 6.92–6.93 (d, 1H, *J* = 7.38 Hz), *δ* 7.26–7.30 (t, 1H, *J* = 7.70 Hz), *δ* 10.99 (s, 1H). ^13^C NMR (100 MHz, DMSO-d_6_): *δ* 23.32, 24.94, 26.48, 28.24, 28.66, 31.72, 35.22, 43.01, 46.18, 51.96, 79.84, 110.40, 112.19, 126.94, 129.67, 132.52, 144.22, 169.36, 171.69, 172.72, 173.34. LCMS-ESI (*m*/*z*): C_23_H_31_N_3_O_5_ (429.52) [M − H^+^] 428.2; retention time 7.70 min (method E).

#### 6-((2-(2,6-Dioxopiperidin-3-yl)-1-oxoisoindolin-4-yl)amino)hexanoic acid (2, **JP-164-05**)

1.0 g 1 was suspended in 4 ml DCM, while 2 ml TFA was added dropwise into the suspension. The suspension was immediately dissolved and was left stirring for another 2 h. After the checking of completion of the reaction, the mixture was evaporated using a rotary evaporator and dissolved in water, transferred to a vial and freeze dried to obtain the product without further purification.^25^^1^H NMR (400 MHz, DMSO-d_6_): *δ* 1.36–1.42 (m, 2H), *δ* 1.51–1.61 (m, 4H), *δ* 2.03–2.05 (m, 1H), *δ* 2.20–2.24 (t, 2H, *J* = 7.28 Hz), *δ* 2.29–2.33 (m, 1H), *δ* 2.60–2.65 (m, 1H), *δ* 2.90–2.93 (m, 1H), *δ* 3.11–3.14 (t, 2H, *J* = 6.98 Hz), *δ* 4.13–4.27 (q, 2H, *J* = 19.40 Hz), *δ* 5.10–5.14 (dd, 1H, *J* = 5.04, 13.27 Hz), *δ* 6.77–6.79 (d, 1H, *J* = 8.05 Hz), *δ* 6.95–6.97 (d, 1H, *J* = 7.42 Hz), *δ* 7.28–7.32 (t, 1H, *J* = 7.71 Hz), *δ* 11.01 (s, 1H). ^13^C NMR (100 MHz, DMSO-d_6_): *δ* 23.28, 24.81, 26.64, 28.62, 31.71, 34.12, 43.32, 46.18, 51.97, 110.89, 112.72, 127.25, 129.71, 132.58, 143.79, 169.29, 171.70, 173.36, 174.92. LCMS-ESI (*m*/*z*): C_19_H_23_N_3_O_5_ (373.41) [M + H^+^] 374.1; retention time 5.65 min (method E); purity 98.47%.

#### Methyl (*S*)-4-(5-amino-4-(2-(hydroxymethyl)pyrrolidine-1-carbonyl)-2-methoxyphenoxy)butanoate (4, **JP-179-02**)

Methyl (*S*)-4-(4-(2-(hydroxymethyl)pyrrolidine-1-carbonyl)-2-methoxy-5-nitrophenoxy)butanoate (3, **MMH-165-31**, 1.440 g, 3.63 mmol, 1 eq.) was dissolved in 10 mL EA/EtOH solvent mixture (EA/EtOH = 5/5). Ammonium formate (0.458 g, 7.26 mmol, 2 eq.) and 10% Pd/C were added to the mixture and stirred in a Parr hydrogenator at 40 Psi for 2 h. After checking the completion of the reaction in LCMS, the mixture was filtered in Celite and washed with EtOH. The collected EtOH solution was evaporated and dried to gain 1.58 g product, which was then directly used for the next step.^12^^1^H NMR (400 MHz, CDCl_3_): *δ* 1.72–2.01 (m, 3H), *δ* 2.14–2.23 (m, 3H), *δ* 2.49–2.61 (t, 5H, *J* = 7.13 Hz), *δ* 3.11–3.18 (t, 2H, *J* = 6.52 Hz), *δ* 3.70 (s, 1H), *δ* 3.97 (s, 3H), *δ* 4.13–4.16 (t, 2H, *J* = 6.23 Hz), *δ* 4.40 (m, 1H), *δ* 6.80 (s, 1H), *δ* 7.70 (s, 1H). ^13^C NMR (100 MHz, CDCl_3_): *δ* 24.20–24.43, 28.48, 30.30, 49.55, 50.86, 51.77, 56.64–56.73, 61.59, 68.40, 108.39, 109.18, 127.82, 137.10, 148.47, 154.86, 173.25. LCMS-ESI (method E) (*m*/*z*): C_18_H_24_N_2_O_8_ (396.4) [M + H^+^] 397.1; retention time 6.31 min.

#### Methyl (*S*)-4-(5-(((allyloxy)carbonyl)amino)-4-(2-(hydroxymethyl)pyrrolidine-1-carbonyl)-2-methoxyphenoxy)butanoate (5, **JP-179-04**)

Allyl chloroformate (0.210 g, 1.74 mmol, 1.1 eq.) in 14 mL anhydrous DCM was added dropwise to a solution of 4 (0.637 g, 2.18 mmol, 1 eq.) dissolved in 19 mL anhydrous DCM and 0.32 mL pyrimidine at −10 °C. The mixture was then allowed to stir at room temperature for another 2 h. Once the reaction was finished, the mixture was washed with CuSO_4_ (32 mL), H_2_O (32 mL), sat. NaHCO_3_ (32 mL), and brine (32 mL), then dried over MgSO_4_. The crude was evaporated and purified *via* flash column chromatography (0–80% EA in hexane), gaining 0.373 g product (yield%: 47.62%).^12^*R*_f_ = 0.32 (TLC: Hex/EA = 2/8). ^1^H NMR (400 MHz, CDCl_3_): *δ* 1.64–1.66 (m, 2H), *δ* 1.79–1.91 (m, 1H), *δ* 2.05–2.18 (m, 3H), *δ* 2.41–2.56 (m, 2H), *δ* 2.97 (s, 2H), *δ* 3.42–3.57 (m, 2H), *δ* 3.62 (s, 3H), *δ* 3.76 (s, 3H), *δ* 3.99–4.12 (m, 3H), *δ* 4.20–4.44 (m, 1H), *δ* 4.56–4.57 (d, 2H, *J* = 5.60 Hz), *δ* 5.17–5.19 (d, 1H, *J* = 10.41 Hz), *δ* 5.26–5.31 (d, 1H, *J* = 17.22 Hz), *δ* 5.86–5.90 (m, 1H), *δ* 6.77 (s, 1H), *δ* 7.20 (s, 1H), *δ* 7.66 (s, 1H). LCMS-ESI (method E) (*m*/*z*): C_22_H_30_N_2_O_8_ (450.49) [M + H^+^] 451.2; retention time 6.66 min.

#### Allyl 11-hydroxy-7-methoxy-8-(4-methoxy-4-oxobutoxy)-5-oxo-2,3,11,11*a*-tetrahydro-1*H*-benzo[*e*]pyrrolo[1,2-*a*][1,4]diazepine-10(5*H*)-carboxylate (6, **JP-179-05**)

TEMPO (0.099 g, 0.63 mmol, 1 eq.) was added to the solution of 5 (2.841 g, 6.31 mmol, 10 eq.) and BAIB (3.557 g, 11.04 mmol, 12 eq.) dissolved in 145.1 mL DCM, and stirred for 6 h. Once the reaction was finished, the mixture was quenched and washed with 65 mL sat. sodium metabisulfite, 65 mL sat. NaHCO_3_, 65 mL water, and 65 mL brine. The crude was dried over MgSO_4_ and purified with flash column chromatography (0–80% EA in hexane). *R*_f_ = 0.16 (TLC: EA/hexane = 7/3). 1.90 g product was obtained (yield: 67.2%).^12^^1^H NMR (400 MHz, CDCl_3_): *δ* 1.88–2.00 (m, 2H), *δ* 2.02–2.12 (m, 4H), *δ* 2.47 (t, 2H, *J* = 7.18 Hz), *δ* 3.36–3.46 (m, 1H), *δ* 3.46–3.55 (m, 1H), *δ* 3.59–3.67 (m, 4H), *δ* 3.83 (s, 3H), *δ* 3.93–4.03 (m, 2H), *δ* 4.37–4.40 (m, 1H), *δ* 4.59–4.63 (dd, 1H, *J* = 5.45, 13.28 Hz), *δ* 5.03–5.15 (d, 2H, *J* = 12.84 Hz), *δ* 5.54–5.56 (d, 1H, *J* = 9.88 Hz), *δ* 5.72–5.74 (m, 1H), *δ* 6.61 (s, 1H), *δ* 7.16 (s, 1H), *δ* 7.20 (s, 1H). LCMS-ESI (method E) (*m*/*z*): C_22_H_28_N_2_O_8_ (448.47) [M + H^+^] 449.2; retention time 5.97 min.

#### Allyl 7-methoxy-8-(4-methoxy-4-oxobutoxy)-5-oxo-11-((tetrahydro-2*H*-pyran-2-yl)oxy)-2,3,11,11*a*-tetrahydro-1*H*-benzo[*e*]pyrrolo[1,2-*a*][1,4]diazepine-10(5*H*)-carboxylate (7, **JP-179-06**)

6 (0.394 g, 0.88 mmol) was dissolved in 6 mL EA added with 0.8 mL DHP (8.78 mmol) and 0.004 g PTSA (0.021 mmol), stirring for 2 h. After checking that the reaction was completed, the crude was diluted in 6 mL EA and washed with 6 mL NaHCO_3_, 6 mL brine. The organic phase was dried over MgSO_4_ and evaporated. The received oil was then purified *via* flash column chromatography (0–80% EA in hexane). *R*_f_ = 0.22 (TLC: EA/hexane = 5/5). The product was obtained after evaporation in a rotary evaporator and drying under high vac (yield: 90.1%).^12^^1^H NMR (400 MHz, CDCl_3_): *δ* 1.32–1.83 (m, 12H), *δ* 1.86–2.15 (m, 13H), *δ* 2.45–2.49 (t, 4H, *J* = 7.10 Hz), *δ* 3.37–3.55 (m, 5H), *δ* 3.61 (s, 7H), *δ* 3.81 (s, 7H), *δ* 3.92–4.03 (m, 4H), *δ* 4.25–4.43 (m, 2H), *δ* 4.49–4.63 (m, 2H), *δ* 4.95–5.12 (m, 4H), *δ* 5.65–5.73 (m, 1H), *δ* 5.78–5.86 (m, 1H), *δ* 6.53 (s, 1H), *δ* 6.80 (s, 1H), *δ* 7.13–7.20 (m, 2H). LCMS-ESI (method E) (*m*/*z*): C_27_H_36_N_2_O_9_ (532.59) [M + H^+^] 533.2; retention time 7.62 min.

#### 4-((10-((Allyloxy)carbonyl)-7-methoxy-5-oxo-11-((tetrahydro-2*H*-pyran-2-yl)oxy)-2,3,5,10,11,11*a*-hexahydro-1*H*-benzo[*e*]pyrrolo[1,2-*a*][1,4]diazepin-8-yl)oxy)butanoic acid (8, **JP-179-07**

7 (0.586 g) was dissolved in 6 mL dioxane, while 3.3 mL 2 M NaOH was added to the mixture, leaving the system at room temperature to stir for 2 h. After checking the completion of the reaction, the mixture was evaporated and dissolved in 30 mL water. 1 M citric acid was titrated till pH = 3–4, and then the water layer was extracted with EA (2 × 20 mL). The extracted EA layer was then collected and washed with brine (20 mL), and dried over MgSO_4_. 0.528 g product was achieved after evaporation and drying (92.5%).^12^^1^H NMR (400 MHz, CDCl_3_): *δ* 1.35–1.61 (m, 8H), *δ* 1.64–1.88 (m, 4H), *δ* 1.88–1.97 (m, 4H), *δ* 2.01–2.19 (m, 8H), *δ* 2.43–2.58 (m, 4H), *δ* 3.34–3.75 (m, 8H), *δ* 3.84 (s, 7H), *δ* 3.98–4.08 (m, 4H), *δ* 4.26–4.43 (m, 2H), *δ* 4.48–4.66 (m, 2H), *δ* 4.86–5.11 (m, 4H), *δ* 5.54–5,75 (m, 2H), *δ* 6.51 (s, 1H), *δ* 6.67 (s, 1H), *δ* 7.15–7.20 (m, 2H). ^13^C NMR (100 MHz, CDCl_3_): *δ* 14.19, 19.72, 21.04, 23.06, 23.94, 24.05, 25.26, 25.44, 28.70, 30.10–30.19, 30.68, 30.93, 46.44, 56.13, 60.09, 60.42, 62.91, 66.88, 67.82, 86.01, 94.65, 110.82, 114.11–114.55, 125.89, 167.13, 177.33. LCMS-ESI (method E) (*m*/*z*): C_26_H_34_N_2_O_9_ (518.56) [M + H^+^] 519.2; retention time 6.96 min.

#### *tert*-Butyl(2-fluoro-4-nitrophenyl)carbamate (10a, **JP-175-P1**)

2-Fluoro-4-nitroaniline (9a, 0.100 g, 0.641 mmol, 1 eq.) was suspended in 3 mL DCM with TEA (0.13 mL, 0.962 mmol, 1 eq.) and DMAP (0.009 g, 0.077 mmol, 0.03 eq.). Di-*tert*-butyl decarbonate (0.147 g, 0.673 mmol, 1.1 eq.) was added to the system, stirring it at r.t for 16 h. After checking the completion of the reaction, the mixture was diluted in 4 mL DCM and washed with ice-cooled 5% citric acid (4 × 3 mL) and dried over Na_2_SO_4_. The dried crude was then purified using flash column chromatography (0–5% EA in hexane). *R*_f_ = 0.41 (TLC: hexane/EA = 9/1). 0.092 g product was achieved (yield: 56.2%). ^1^H NMR (400 MHz, CDCl_3_): *δ* 1.54 (s, 9H), *δ* 6.99 (s, 1H), *δ* 7.95–7.99 (dd, 1H, *J* = 2.49, 10.91 Hz), *δ* 8.04–8.06 (m, 1H), *δ* 8.34–8.38 (t, 1H, *J* = 8.52 Hz). LCMS-ESI (method E) (*m*/*z*): C_11_H_13_FN_2_O_4_ (256.23) [M − H^+^] 255.1; retention time 8.28 min.

#### *tert*-Butyl(4-amino-2-fluorophenyl)carbamate (11a, **JP-175-P2**)

10a (0.300 g, 1.17 mmol, 1 eq.) was dissolved in EA/EtOH solvent mixture (EA/EtOH = 2/3), while ammonium formate (0.148 g, 2.34 mmol, 2 eq.) and 10% Pd/C (0.030 g) were added to the system. The mixture was reacted in a hydrogenator at 40 Psi overnight. The product was filtered in Celite and washed with EtOH. The product was evaporated without further purification, gaining 0.118 g product (yield: 44.5%). *R*_f_ = 0.86 (TLC: 100% EA). ^1^H NMR (400 MHz, DMSO-d_6_): *δ* 1.41 (s, 9H), *δ* 5.21 (s, 2H), *δ* 6.30 (m, 2H), *δ* 6.95 (s, 1H), *δ* 8.27 (s, 1H). ^13^C NMR (100 MHz, DMSO-d_6_): *δ* 28.59, 39.36–40.61, 78.81, 100.73–100.96, 109.68–109.70, 114.00–114.12, 128.36, 148.27–148.37, 154.45, 156.16, 158.57. LCMS-ESI (method E) (*m*/*z*): C_11_H_15_FN_2_O_2_ (226.25) [M + H^+^] 227.1; retention time 5.77 min.

#### *tert*-Butyl(4-(6-((2-(2,6-dioxopiperidin-3-yl)-1-oxoisoindolin-4-yl)amino)hexanamido)-2-fluorophenyl)carbamate (12a, **JP-175-P3**)

2 (0.080 g, 0.214 mmol, 1 eq.) was dissolved in 5 mL DMF added with DMAP (0.052 g, 0.428 mmol, 2 eq.) and EDC (0.103 g, 0.535 mmol, 2.5 eq.). The mixture was stirred for 30 min and then added 11a (0.073 g, 0.321 mmol, 1.2 eq.). The mixture was quenched with 50 mL water, and it was then worked up with 3 × 20 mL EA. The organic layer was collected and washed with 3 × 50 mL water, 50 mL brine, and dried over MgSO_4_. The dried crude was purified using flash column chromatography (0–70% EA in hexane). *R*_f_ = 0.06 (TLC: EA/hexane = 8/2). 0.034 g product was achieved (yield: 27.6%). ^1^H NMR (400 MHz, MeOD-d_4_): *δ* 1.30 (s, 2H), *δ* 1.53 (s, 9H), *δ* 1.69–1.80 (m, 4H), *δ* 2.15–2.21 (m, 1H), *δ* 2.38–2.41 (t, 2H, *J* = 7.44 Hz), *δ* 2.44–2.52 (m, 1H), *δ* 2.77–2.82 (m, 1H), *δ* 2.88–2.98 (m, 1H), *δ* 3.25–3.28 (t, 2H, *J* = 7.09 Hz), *δ* 4.28 (s, 2H), *δ* 5.13–5.17 (dd, 1H, *J* = 5.80, 13.26 Hz), *δ* 6.84–6.86 (d, 1H, *J* = 7.94 Hz), *δ* 7.07–7.09 (d, 1H, *J* = 7.94 Hz), *δ* 7.14–7.16 (d, 1H, *J* = 8.83 Hz), *δ* 7.31–7.35 (t, 1H, *J* = 7.50 Hz), *δ* 7.57–7.64 (m, 2H). ^13^C NMR (100 MHz, MeOD-d_4_): *δ* 22.85, 25.06, 26.31, 27.22, 28.43, 29.34, 29.48, 30.99, 36.34, 42.73, 46.96–48.23, 52.16, 107.16, 110.50, 112.46, 129.19, 143.70, 170.89, 173.30. LCMS-ESI (method E) (*m*/*z*): C_30_H_36_FN_5_O_6_ (581.65) [M + H^+^] 582.2; retention time 7.47 min.

#### *N*-(4-Amino-3-fluorophenyl)-6-((2-(2,6-dioxopiperidin-3-yl)-1-oxoisoindolin-4-yl)amino)hexanamide (13a, **JP-175-P4**)

12a (0.060 g, 0.103 mmol, 1 eq.) was dissolved in 4 mL DCM, added with 2 mL TFA. The solvent was stirred for 1 h. After checking that the reaction was completed, the product was evaporated and directly used for the next step without further purification. ^1^H NMR (400 MHz, MeOD-d_4_): *δ* 1.34–1.44 (m, 2H), *δ* 1.55–1.69 (m, 4H), *δ* 2.03–2.11 (m, 1H), *δ* 2.23–2.41 (m, 4H), *δ* 2.66–2.70 (m, 1H), *δ* 2.77–2.86 (m, 1H), *δ* 3.14–3.18 (t, 2H, *J* = 7.20 Hz), *δ* 4.17–4.18 (d, 2H, *J* = 4.59 Hz), *δ* 5.02–5.06 (dd, 1H, *J* = 5.32, 12.92 Hz), *δ* 6.75–6.77 (d, 1H, *J* = 8.12 Hz), *δ* 6.97–6.99 (d, 1H, *J* = 7.17 Hz), *δ* 7.16–7.23 (m, 3H), *δ* 7.61–7.64 (d, 1H, *J* = 13.38 Hz). ^13^C NMR (100 MHz, MeOD-d_4_): *δ* 22.84, 24.90, 26.22, 28.32, 29.33, 30.97, 36.30, 42.98, 45.92, 46.96–48.23, 52.17, 107.65, 110.99, 112.96, 115.76, 126.92, 129.21, 131.62, 143.23, 170.90–170.01, 173.27. LCMS-ESI (method E) (*m*/*z*): C_25_H_28_FN_5_O_4_ (481.53) [M + H^+^] 482.2; retention time 5.87 min.

#### Allyl 8-(4-((4-(6-((2-(2,6-dioxopiperidin-3-yl)-1-oxoisoindolin-4-yl)amino)hexanamido)-2-fluorophenyl)amino)-4-oxobutoxy)-7-methoxy-5-oxo-11-((tetrahydro-2*H*-pyran-2-yl)oxy)-2,3,11,11*a*-tetrahydro-1*H*-benzo[*e*]pyrrolo[1,2-*a*][1,4]diazepine-10(5*H*)-carboxylate (14a, **JP-175-P5**)

5 (0.040 g, 0.077 mmol, 1 eq.) was dissolved in 3 mL DMF, added with DMAP (0.019 g, 0.154 mmol, 2 eq.) and EDC (0.037 g, 0.193 mmol, 2.5 eq), and the mixture was stirred for 30 min then added 13a (0.044 g, 0.092 mmol, 1.2 eq.), stirring overnight. After confirming the reaction was finished *via* TLC, the product was quenched with 10× volume of water, and it was worked up with 3 × 30 mL EA. The organic phase was combined and washed with 50 mL water, 50 mL brine, and subsequently dried over MgSO_4_. The crude was purified using flash column chromatography (0–5% MeOH in EA). *R*_f_ = 0.42 (TLC: EA/MeOH = 9/1) 0.034 g product was gained (yield: 44.3%). ^1^H NMR (400 MHz, MeOD-d_4_): *δ* 1.46–1.58 (m, 6H), *δ* 1.67–1,79 (m, 6H), *δ* 1.98–2.07 (m, 3H), *δ* 2.09–2.26 (m, 5H), *δ* 2.35–2.47 (m, 3H), *δ* 2.59–2.68 (m, 2H), *δ* 2.73–2.82 (m, 1H), *δ* 2.84–2.99 (m, 1H), *δ* 3.23–3.26 (t, 2H, *J* = 6.80 Hz), *δ* 3.44–3.66 (m, 5H), *δ* 3.87–3.93 (s, 3H), *δ* 4.07–4.15 (m, 2H), *δ* 4.26 (d, 2H, *J*= 2.38 Hz), *δ* 4.40–4.63 (m, 2H), *δ* 5.00–5.16 (m, 3H), *δ* 5.73–5.82 (m, 1H), *δ* 5.87–5.89 (d, 1H, *J* = 9.48 Hz), *δ* 6.82–6.84 (d,1H, *J* = 8.18 Hz), *δ* 6.96–6.97 (d, 1H, *J* = 4.54 Hz), *δ* 7.05–7.07 (d, 1H, *J* = 7.50 Hz), *δ* 7.14–7.20 (m, 2H), *δ* 7.29–7.33 (t, 1H, *J* = 7.75 Hz), *δ* 7.60–7.63 (d, 1H, *J* = 12.86 Hz), *δ* 7.72–7.77 (t, 1H, *J* = 8.57 Hz). LCMS-ESI (method E) (*m*/*z*): C_51_H_60_FN_7_O_12_ (982.08) [M^+^] 982.5; retention time 7.41 min.

#### 6-((2-(2,6-Dioxopiperidin-3-yl)-1-oxoisoindolin-4-yl)amino)-*N*-(3-fluoro-4-(4-(((*S*)-7-methoxy-5-oxo-2,3,5,11*a*-tetrahydro-1*H*-benzo[*e*]pyrrolo[1,2-*a*][1,4]diazepin-8-yl)oxy)butanamido)phenyl)hexanamide (15a, **JP-175-P6**)

14a (0.097 g, 0.099 mmol, 1 eq.) was dissolved in 5 mL DCM, added with palladium tetrakis[triphenylphosphine] (0.0057 g, 0.005 mmol, 0.05 eq.), triphenylphosphine (0.0065 g, 0.025 mmol, 0.25 eq.), and pyrrolidine (0.01 mL, 0.119 mmol, 0.25 eq.). The mixture was stirred at room temperature for 2 h. The mixture was evaporated and dried, which was purified using flash column chromatography (0–20% MeOH in EA). *R*_f_ = 0.08 (TLC: EA/MeOH = 8/2). 0.093 g product was retrieved (yield > 99.9%). ^1^H NMR (400 MHz, DMSO-d_6_): *δ* 1.59–1.70 (m, 2H), *δ* 1.60–1.70 (m, 4H), *δ* 1.87–1.94 (m, 2H), *δ* 2.02–2.09 (m, 5H), *δ* 2.18–2.21 (m, 2H), *δ* 2.32–2.37 (t, 2H, *J* = 7.18 Hz), *δ* 3.06–3.15 (m, 5H), *δ* 3.46–3.52 (m, 2H), *δ* 3.55–3.59 (m, 1H), *δ* 3.63–3.66 (m, 3H), *δ* 3.91–4.04 (m, 2H), *δ* 4.18–4.28 (m, 1H), *δ* 4.42–4.51 (m, 1H), *δ* 4.65–4.83 (m, 1H), *δ* 5.69 (s, 1H), *δ* 6.41 (s, 1H), *δ* 6.69–6.71 (d, 1H, *J* = 8.05 Hz), *δ* 6.87–6.89 (d, 1H, *J* = 7.38 Hz), *δ* 7.15 (s, 1H), *δ* 7.23–7.27 (m, 2H), *δ* 7.34 (s, 1H), *δ* 7.56 (s, 1H), *δ* 7.63–7.67 (m, 1H), *δ* 7.78–7.79 (d, 1H, *J* = 4.41 Hz), *δ* 9.70–9.71 (d, 1H, *J* = 4.09 Hz), *δ* 10.29 (s, 1H). ^13^C NMR (100 MHz, DMSO-d_6_): *δ* 22.87, 23.16–23.29, 24.13–24.32, 25.09, 25.42–25.64, 25.98, 26.84, 28.78, 29.29, 30.70, 31.17–31.49, 32.46, 36.82, 43.09, 44.78, 45.69, 46.11–46.26, 46.84, 49.04, 52.66, 53.88–54.22, 56.09–56.35, 58.57, 67.72, 106.54–106.78, 110.10–110.63, 111.64, 114.84–115.40, 120.26–121.06, 125.58, 127.40, 129.37, 132.74, 139.83–141.06, 144.13, 147.40, 152.00, 155.49, 163.84, 164.71, 165.31, 169.39–169.80, 171.31–171.95, 172.92. LCMS-ESI (method E) (*m*/*z*): retention time at 6.2 min, purity 97.07%. LCMS-ESI (method F) (*m*/*z*): retention time at 2.78 min, purity >98.5%. HRMS-ESI-ESI: C_42_H_46_FN_7_O_8_ (795.35) [M + H^+^] calculated for 796.3465; found 796.3438; error −3.35 ppm.

#### 6-((2-(2,6-Dioxopiperidin-3-yl)-1-oxoisoindolin-4-yl)amino)-*N*-(4-(4-(((S)-7-methoxy-5-oxo-2,3,5,11*a*-tetrahydro-1*H*-benzo[*e*]pyrrolo[1,2-*a*][1,4]diazepin-8-yl)oxy)butanamido)phenyl)hexanamide (15d, **JP-163-16**)

2 (0.053 g, 0.143 mmol, 1.5 eq.) was dissolved in 3 mL DMF, added with DMAP (0.023 g, 0.190 mmol, 2 eq.) and EDC (0.046 g, 0.238 mmol, 2.5 eq.). The mixture was stirred for 30 min and then added **MMH-165-26** (20d, 0.040 g, 0.095 mmol, 1 eq.) and it was stirred overnight. Once the reaction was finished, the reaction was quenched with 10× volume of water, and extracted with 3 × 30 mL EA. The organic layer was collected and washed with 3 × 30 mL water and 30 mL brine, dried over MgSO_4_. The crude was purified *via* C_18_ preparative column chromatography (method C), gaining 0.004 g product (yield: 7.4%). ^1^H NMR (400 MHz, DMSO-d_6_): *δ* 1.39–1.45 (m, 2H), *δ* 1.60–1.65 (m, 4H), *δ* 1.80–1.99 (m, 4H), *δ* 2.00–2.12 (m, 4H), *δ* 2.16–2.25 (m, 1H), *δ* 2.28–2.35 (m, 3H), *δ* 2.60–2.64 (m, 1H), *δ* 2.88–2.97 (m, 1H), *δ* 3.11–3.16 (m, 2H), *δ* 3.43–3.52 (m, 2H), *δ* 3.56–3.62 (m, 1H), *δ* 3.66 (s, 3H), *δ* 3.93–4.03 (m, 2H), *δ* 4.15–4.25 (q, 2H, *J* = 19.16 Hz), *δ* 5.09–5.14 (dd, 1H, *J* = 5.18, 13.30 Hz), *δ* 5.59 (t, 1H, *J* = 5.48 Hz), *δ* 6.37 (s, 1H), *δ* 6.74–6.76 (d, 1H, *J* = 8.06 Hz), *δ* 6.92–6.94 (d, 1H, *J* = 7.41 Hz), *δ* 7.05 (s, 1H), *δ* 7.21–7.34 (m, 2H), *δ* 7.50 (s, 4H), *δ* 9.79 (s, 1H), *δ* 9.89 (s, 1H), *δ* 11.00 (s, 1H). ^13^C NMR (100 MHz, DMSO-d_6_): *δ* 23.31, 24.13, 24.97, 25.49, 26.83, 28.85, 29.31, 29.44, 30.87, 31.72, 33.06, 36.80, 43.11, 46.23, 46.84, 49.07, 51.99, 53.88, 56.11, 68.32, 110.40, 110.72, 111.81, 112.25, 119.86–119.92, 120.31, 126.94, 129.68, 132.53, 134.99, 135.12, 141.08, 144.25, 147.45, 150.70, 163.82, 164.69, 169.36, 170.68, 171.32, 171.70, 173.33. LCMS-ESI (method E) (*m*/*z*): C_42_H_47_N_7_O_8_ (777.88) [M + H^+^] 778.3; retention time 6.19 min, purity >98.5%. LCMS-ESI (method F) (*m*/*z*), retention time at 2.78 min, purity 95.8%. HRMS-ESI (*m*/*z*): C_42_H_47_N_7_O_8_ calculated [M + H^+^] 778.35589; found 778.3549; error −1.32 ppm.

#### *tert*-Butyl(2-methyl-4-nitrophenyl)carbamate (10b, **JP-179-P1**)

2-Methyl-4-nitroaniline (9b, 0.800 g, 5.26 mmol, 1 eq.) was suspended in 5 mL DCM with TEA (1.1 mL, 7.89 mmol, 1.5 eq.) and DMAP (0.077 g, 0.63 mmol, 0.12 eq.). Di-*tert*-butyl decarbonate (1.205 g, 5.52 mmol, 1.05 eq.) was added to the system, leaving it stirring at r.t for 16 h. After checking the completion of the reaction, the mixture was diluted in 30 mL DCM, and washed with ice-cooled 5% citric acid (4 × 20 mL) and dried over MgSO_4_. The dried crude was then purified using flash column chromatography (0–5% EA in hexane). *R*_f_ = 0.16 (TLC: hexane/EA = 9/1). 0.465 g product was achieved (yield: 35.0%). ^1^H NMR (400 MHz, CDCl_3_): *δ* 1.55 (s, 9H), *δ* 2.33 (s, 3H), *δ* 6.58 (s, 1H), *δ* 8.04–8.09 (m, 2H), *δ* 8.21–8.24 (d, 1H, *J* = 9.07 Hz). ^13^C NMR (100 MHz, CDCl_3_): *δ* 17.67, 28.23, 81.97, 118.12, 123.10, 125.44–125.65, 142.37–142.76, 151.90. LCMS-ESI (method E) (*m*/*z*): C_12_H_16_N_2_O_4_ (252.27) [M − H^+^] 251.1; retention time 8.27 min.

#### *tert*-Butyl(4-amino-2-methylphenyl)carbamate (11b, **JP-179-P2**)

10b (0.200 g, 0.79 mmol, 1 eq.) was dissolved in EA/EtOH solvent mixture (EA/EtOH = 2/3), while ammonium formate (0.100 g, 1.58 mmol, 2 eq.) and 10% Pd/C (0.020 g) were added to the system. The mixture was reacted in a hydrogenator at 40 psi overnight. The product was filtered in Celite and washed with EtOH. The product was evaporated without further purification, yielding 0.192 g product. ^1^H NMR (400 MHz, DMSO-d_6_): *δ* 1.41 (s, 9H), *δ* 2.02 (s, 3H), *δ* 4.83 (s, 2H), *δ* 6.30–6.33 (dd, 1H, *J* = 2.47, 8.31 Hz), *δ* 6.36–6.37 (d, 1H, *J* = 2.26 Hz), *δ* 6.77–6.79 (d, 1H, *J* = 8.27 Hz), *δ* 8.06 (s, 1H). ^13^C NMR (100 MHz, DMSO-d_4_): *δ* 18.36, 28.69, 78.28, 111.91, 115.70, 125.73, 127.65, 134.35, 146.71, 154.77. LCMS-ESI (method E) (*m*/*z*): C_12_H_18_N_2_O_2_ (222.28) [M + H^+^] 223.1; retention time 4.74 min.

#### *tert*-Butyl(4-(6-((2-(2,6-dioxopiperidin-3-yl)-1-oxoisoindolin-4-yl)amino)hexanamido)-2-methylphenyl)carbamate (12b, **JP-179-P3**)

2 (0.258 g, 0.690 mmol, 1 eq.) was dissolved in 3 mL DMF added with DMAP (0.169 g, 1.38 mmol, 2 eq.) and EDC (0.331 g, 1.725 mmol, 2.5 eq.). The mixture was stirred for 30 min and then added 11b (0.184 g, 0.828 mmol, 1.2 eq.). The mixture was quenched with 10× volume water, and it was then worked up with 3 × 20 mL EA. The organic layer was collected and washed with 3 × 30 mL water, 30 mL brine, and dried over MgSO_4_. The dried crude was purified using flash column chromatography (0–90% EA in hexane). *R*_f_ = 0.08 (TLC: EA/hexane = 9/1). 0.074 g product was achieved (yield: 18.5%). ^1^H NMR (400 MHz, DMSO-d_6_): *δ* 1.44 (m, 11H), *δ* 1.58–1.66 (m, 4H), *δ* 1.98–2.00 (m, 1H), *δ* 2.14 (s, 3H), *δ* 2.27–2.33 (m, 3H), *δ* 2.60–2.67 (m, 1H), *δ* 2.88–2.97 (m, 1H), *δ* 3.11–3.18 (m, 2H), *δ* 4.10–4.25 (q, 2H, *J* = 19.23 Hz), *δ* 5.09–5.13 (dd, 1H, *J* = 5.05, 13.24 Hz), *δ* 5.56–5.59 (t, 1H, *J* = 5.27 Hz), *δ* 6.74–6.76 (d, 1H, *J* = 8.05 Hz), *δ* 6.92–6.93 (d, 1H, *J* = 7.44 Hz), *δ* 7.14–7.17 (d, 1H, *J* = 8.88 Hz), *δ* 7.26–7.34 (m, 2H), *δ* 7.40 (s, 1H), *δ* 8.40 (s, 1H), *δ* 9.75 (s, 1H), *δ* 11.00 (s, 1H). LCMS-ESI (method E) (*m*/*z*): C_31_H_39_N_5_O_6_ (577.68) [M + H^+^] 578.3; retention time 7.34 min.

#### *N*-(4-Amino-3-methylphenyl)-6-((2-(2,6-dioxopiperidin-3-yl)-1-oxoisoindolin-4-yl)amino)hexanamide (13b, **JP-179-P4**)

12b (0.103 g, 0.178 mmol, 1 eq.) was dissolved in 4 mL DCM, added with 2 mL TFA. The solvent was stirred for 1 h. After checking that the reaction was completed, the product was evaporated and directly used for the next step without further purification. ^1^H NMR (400 MHz, MeOD-d_4_): *δ* 1.48–1.54 (m, 2H), *δ* 1.60–1.84 (m, 4H), *δ* 2.14–2.19 (m, 1H), *δ* 2.35–2.43 (m, 5H), *δ* 2.45–2.60 (m, 1H), *δ* 2.74–2.94 (m, 2H), *δ* 3.22–3.26 (t, 2H, *J* = 7.00 Hz) *δ* 4.25–4.27 (d, 2H, *J* = 4.81 Hz), *δ* 5.11–5.15 (dd, 1H, *J* = 5.14, 13.29 Hz), *δ* 6.82–6.84 (d, 1H, *J* = 8.01 Hz), *δ* 7.04–7.06 (d, 1H, *J* = 7.48 Hz), *δ* 7.23–7.31 (m, 2H), *δ* 7.50–7.52 (m, 2H). ^13^C NMR (100 MHz, MeOD-d_4_): *δ* 15.82, 22.84, 25.03, 26.28, 28.42, 29.33–29.49, 30.97, 36.36, 42.87, 45.95, 52.17, 110.69, 112.68, 118.34, 122.37, 123.06, 124.84, 126.75, 129.18, 131.57, 132.18, 143.51, 170.91, 173.27–173.32. LCMS-ESI (method E) (*m*/*z*): C_26_H_31_N_5_O_4_ (477.57) [M + H^+^] 478.2; retention time 4.97 min.

#### Allyl 8-(4-((4-(6-((2-(2,6-dioxopiperidin-3-yl)-1-oxoisoindolin-4-yl)amino)hexanamido)-2-methylphenyl)amino)-4-oxobutoxy)-7-methoxy-5-oxo-11-((tetrahydro-2*H*-pyran-2-yl)oxy)-2,3,11,11*a*-tetrahydro-1*H*-benzo[*e*]pyrrolo[1,2-*a*][1,4]diazepine-10(5*H*)-carboxylate (14b, **JP-179-P5**)

8 (0.090 g, 0.174 mmol, 1 eq.) was dissolved in 3 mL DMF, added with DMAP (0.043 g, 0.348 mmol, 2 eq.) and EDC (0.083 g, 0.435 mmol, 2.5 eq.), and the mixture was stirred for 30 min then added 13b (0.100 g, 0.209 mmol, 1.2 eq.), stirring overnight. After confirming that the reaction was finished *via* TLC, the product was quenched with 10× volume of water, and it was worked up with 3 × 30 mL EA. The organic phase was combined and washed with 50 mL water, 50 mL brine, and subsequently dried over MgSO_4_. The crude was purified using flash column chromatography (0–5% MeOH in EA). *R*_f_ = 0.28 (TLC: EA/MeOH = 9/1). 0.075 g product was gained (yield: 44.1%). ^1^H NMR (400 MHz, MeOD-d_4_): *δ* 1.45–1.56 (m, 6H), *δ* 1.68–1.79 (m, 6H), *δ* 1.97–2.07 (m, 3H), *δ* 2.09–2.26 (m, 5H), *δ* 2.33–2.48 (m, 3H), *δ* 2.57–2.70 (m, 2H), *δ* 2.73–3.00 (m, 2H), *δ* 3.22–3.26 (t, 2H, *J* = 6.85 Hz), *δ* 3.41–3.72 (m, 5H), *δ* 3.87–3.93 (m, 3H), *δ* 4.06–4.19 (m, 2H), *δ* 4.26 (s, 2H), *δ* 4.38–4.62 (m, 2H), *δ* 5.00–5.16 (m, 3H), *δ* 5.73–5.82 (m, 1H), *δ* 5.87–5.89 (d, 1H, *J* = 9.48 Hz), *δ* 6.82–6.84 (d, 2H, *J* = 8.18 Hz), *δ* 6.96–6.97 (d, 1H, *J* = 4.54 Hz), *δ* 7.05–7.07 (d, 1H, *J* = 7.50 Hz), *δ* 7.11–7.23 (m, 2H), *δ* 7.29–7.33 (t, 1H, *J* = 7.77 Hz), *δ* 7.60–7.63 (d, 1H, *J* = 12.86 Hz), *δ* 7.72–7.77 (t, 1H, *J* = 8.73 Hz). LCMS-ESI (method E) (*m*/*z*): C_52_H_63_N_7_O_12_ (978.12) [M^+^/2] 489.7; retention time 7.32 min.

#### 6-((2-(2,6-Dioxopiperidin-3-yl)-1-oxoisoindolin-4-yl)amino)-*N*-(4-(4-(((*S*)-7-methoxy-5-oxo-2,3,5,11*a*-tetrahydro-1*H*-benzo[*e*]pyrrolo[1,2-*a*][1,4]diazepin-8-yl)oxy)butanamido)-3-methylphenyl)hexanamide (15b, **JP-179-P6**)

14b (0.075 g, 0.077 mmol, 1 eq.) was dissolved in 5 mL DCM, added with palladium tetrakis[triphenylphosphine] (0.0045 g, 0.004 mmol, 0.05 eq.), triphenylphosphine (0.0050 g, 0.019 mmol, 0.25 eq.), and pyrrolidine (0.008 mL, 0.092 mmol, 1.2 eq.). The mixture was stirred at room temperature for 2 h. The mixture was then evaporated and dried, which was purified using flash column chromatography (0–20% MeOH in EA). *R*_f_ = 0.38 (TLC: EA/MeOH = 8/2). 0.053 g product was retrieved (yield: 86.9%). ^1^H NMR (400 MHz, DMSO-d_6_): *δ* 1.36–1.48 (m, 2H), *δ* 1.57–1.70 (m, 4H), *δ* 1.84–1.98 (m, 2H), *δ* 1.99–2.07 (m, 3H), *δ* 2.12 (s, 3H), *δ* 2.19–2.39 (m, 5H), *δ* 2.49–2.52 (m, 2H), *δ* 2.58–2.68 (d, 1H, *J* = 16.87 Hz), *δ* 2.83–3.00 (m, 1H), *δ* 3.07–3.19 (m, 2H), *δ* 3.33–3.45 (m, 2H), *δ* 3.54–3.62 (m, 1H), *δ* 3.57–3.76 (m, 3H), *δ* 3.97–4.10 (m, 2H), *δ* 4.13–4.27 (m, 2H), *δ* 5.00–5.21 (dd, 1H, *J* = 12.80, 4.68 Hz), *δ* 5.57 (s, 1H), *δ* 6.74–6.76 (d, 1H, *J* = 7.98 Hz), *δ* 6.92–6.94 (d, 1H, *J* = 7.37 Hz), *δ* 7.23–7.30 (m, 2H), *δ* 7.35–7.39 (d, 2H, *J* = 8.11 Hz), *δ* 7.45 (s, 1H), *δ* 7.56 (s, 1H), *δ* 7.78–7.79 (d, 1H, *J* = 4.13 Hz), *δ* 9.28 (s, 1H), *δ* 9,81 (s, 1H), *δ* 11.00 (s, 1H). ^13^C NMR (100 MHz, DMSO-d_6_): *δ* 14.55, 18.56, 21.22, 23.31, 24.14, 25.23, 25.50, 26.81, 28.85, 29.31, 31.72, 36.83, 43.11, 46.25, 46.84, 51.99, 53.88, 54.21, 56.10, 60.22, 68.36, 110.39, 110.70, 111.80, 112.22, 117.23, 121.18, 126.20, 126.95, 129.68, 131.92, 132.53, 132.85, 141.08, 144.25, 147.45, 150.71, 164.70, 169.37, 171.00, 171.48, 171.71, 173.35. LCMS-ESI (method E) (*m*/*z*): C_43_H_49_N_7_O_8_ (791.91) 792.3; retention time 6.16 min; purity 95.66%. LCMS-ESI (method F) (*m*/*z*): retention time at 2.73 min, purity 98.5%. HRMS-ESI (*m*/*z*): C_43_H_49_N_7_O_8_ (791.27) [M + H^+^] calculated 792.3715; found 792.3703; error −1.56 ppm.

#### *tert*-Butyl(2-methoxy-4-nitrophenyl)carbamate (10c, **JP-179-P7**)

2-Methoxy-4-nitroaniline (9c, 0.800 g, 4.76 mmol, 1 eq.) was suspended in 5 mL DCM with TEA (1.0 mL, 7.14 mmol, 1.5 eq.) and DMAP (0.069 g, 0.57 mmol, 0.12 eq.). Di-*tert*-butyl decarbonate (1.091 g, 5.00 mmol, 1.05 eq.) was then added, leaving it stirring at r.t for 16 h. Once the reaction was finished, the mixture was diluted in 30 mL DCM and washed with ice-cooled 5% citric acid (4 × 20 mL) and dried over MgSO_4_. The dried crude was then purified using flash column chromatography (0–5% EA in hexane). *R*_f_ = 0.22 (TLC: hexane/EA = 9/1). 0.764 g product was achieved (yield: 59.7%). ^1^H NMR (400 MHz, CDCl_3_): *δ* 1.54 (s, 9H), *δ* 3.98 (s, 3H), *δ* 7.35 (s, 1H), *δ* 7.72 (s, 1H), *δ* 7.90–7.92 (dd, 1H, *J* = 2.09, 9.04 Hz), *δ* 8.25–8.28 (d, 1H, *J* = 9.02 Hz). ^13^C NMR (100 MHz, CDCl_3_): *δ* 28.24, 56.26, 81.71, 105.18, 116.36, 117.89, 134.66, 142.15, 146.81, 152.00. LCMS-ESI (method E) (*m*/*z*): C_12_H_16_N_2_O_5_ (268.27) [M − H^+^] 267.1; retention time 8.55 min.

#### *tert*-Butyl(4-amino-2-methoxyphenyl)carbamate (11c, **JP-179-P8**)

10c (0.300 g, 1.118 mmol, 1 eq.) was dissolved in EA/EtOH solvent mixture (EA/EtOH = 2/3), while ammonium formate (0.141 g, 2.236 mmol, 2 eq.) and 10% Pd/C were added to the system. The mixture was reacted in a hydrogenator at 40 psi overnight. The product was filtered in Celite and washed with EtOH. The product was evaporated without further purification, yielding 0.267 g product. ^1^H NMR (400 MHz, DMSO-d_6_): *δ* 1.41 (s, 9H), *δ* 3.68 (s, 3H), *δ* 4.92 (s, 2H), *δ* 6.07–6.09 (d, 1H, *J* = 8.37 Hz), *δ* 6.23 (s, 1H), *δ* 7.04 (s, 1H), *δ* 7.52 (s, 1H). ^13^C NMR (100 MHz, DMSO-d_6_): *δ* 28.64, 55.57, 78.55, 98.25, 105.67, 116.27, 147.03, 154.18. LCMS-ESI (method E) (*m*/*z*): C_12_H_18_N_2_O_3_ (238.29) [M + H^+^] 239.1; retention time 5.04 min.

#### *tert*-Butyl(4-(6-((2-(2,6-dioxopiperidin-3-yl)-1-oxoisoindolin-4-yl)amino)hexanamido)-2-methoxyphenyl)carbamate (12c, **JP-179-P9**)

2 (0.261 g, 0.699 mmol, 1 eq.) was dissolved in 3 mL DMF added with DMAP (0.171 g, 1.398 mmol, 2 eq.) and EDC (0.335 g, 1.748 mmol, 2.5 eq.). The mixture was stirred for 30 min and then added 11c (0.200 g, 0.839 mmol, 1.2 eq.). The mixture was quenched with 10× volume water, and it was then worked up with 3 × 20 mL EA. The organic layer was collected and washed with 3 × 30 mL water, 30 mL brine, and dried over MgSO_4_. The dried crude was purified using flash column chromatography (0–90% EA in hexane). *R*_f_ = 0.08 (TLC: EA/hexane = 9/1). 0.152 g product was achieved (yield: 36.6%). ^1^H NMR (400 MHz, MeOD-d_4_): *δ* 1.51 (m, 11H), *δ* 1.70–1.78 (m, 4H), *δ* 2.12–2.17 (m, 1H), *δ* 2.37–2.40 (m, 3H), *δ* 2.86–2.99 (m, 3H), *δ* 3.24–3.28 (t, 2H, *J* = 6.92 Hz), *δ* 3.84 (s, 3H), *δ* 4.26 (s, 2H), *δ* 5.10–5.14 (dd, 1H, *J* = 5.06, 13.38 Hz), *δ* 6.83–6.85 (d, 1H, *J* = 8.08 Hz), *δ* 6.94–6.96 (m, 1H), *δ* 7.05–7.07 (d, 1H, *J* = 7.49 Hz), *δ* 7.29–7.33 (t, 1H, *J* = 7.93 Hz), *δ* 7.39 (s, 1H), *δ* 7.68–7.70 (d, 1H, *J* = 7.90 Hz). LCMS-ESI (method E) (*m*/*z*): C_31_H_39_N_5_O_7_ (593.68) [M + H^+^] found 594.3; retention time 7.59 min.

#### *N*-(4-Amino-3-methoxyphenyl)-6-((2-(2,6-dioxopiperidin-3-yl)-1-oxoisoindolin-4-yl)amino)hexanamide (13c, **JP-179-P10**)

12c (0.144 g, 0.242 mmol, 1 eq.) was dissolved in 4 mL DCM, added with 2 mL TFA. The solvent was stirred for 1 h. After checking the reaction was completed, 0.163 g product was obtained and directly used for the next step without further purification. ^1^H NMR (400 MHz, MeOD-d_4_): *δ* 1.48–1.55 (m, 2H), *δ* 1.70–1.77 (m, 4H), *δ* 2.15–2.18 (m, 1H), *δ* 2.40–2.50 (m, 3H), *δ* 2.75–2.99 (m, 2H), *δ* 3.24–3.27 (t, 2H, *J* = 7.00 Hz), *δ* 3.94 (s, 3H), *δ* 4.27–4.28 (d, 2H, *J* = 4.53 Hz), *δ* 5.12–5.17 (dd, 1H, *J* = 5.15, 13.35 Hz), *δ* 6.83–6.85 (d, 1H, *J* = 7.99 Hz), *δ* 7.05–7.07 (d, 1H, *J* = 7.48 Hz), *δ* 7.13–7.14 (d, 1H, *J* = 10.05 Hz), *δ* 7.24–7.32 (m, 2H), *δ* 7.60 (s, 1H). ^13^C NMR (100 MHz, MeOD-d_4_): *δ* 22.86, 24.99, 26.29, 28.44, 30.97, 36.43, 42.87, 45.91, 48.44, 52.15, 53.73, 55.29, 103.59, 110.72, 111.44, 112.69, 123.42, 126.76, 129.19, 131.57, 143.47, 152.82, 170.90–171.07, 173.27–173.39. LCMS-ESI (method E) (*m*/*z*): C_26_H_31_N_5_O_5_ (493.56) [M + H^+^] 494.2; retention time 4.89 min.

#### Allyl 8-(4-((4-(6-((2-(2,6-dioxopiperidin-3-yl)-1-oxoisoindolin-4-yl)amino)hexanamido)-2-methoxyphenyl)amino)-4-oxobutoxy)-7-methoxy-5-oxo-11-((tetrahydro-2*H*-pyran-2-yl)oxy)-2,3,11,11*a*-tetrahydro-1*H*-benzo[*e*]pyrrolo[1,2-*a*][1,4]diazepine-10(5*H*)-carboxylate (14c, **JP-179-P11**)

8 (0.090 g, 0.174 mmol, 1 eq.) was dissolved in 3 mL DMF, added with DMAP (0.043 g, 0.348 mmol, 2 eq.) and EDC (0.083 g, 0.435 mmol, 2.5 eq.); the mixture was stirred for 30 min then added 13c (0.100 g, 0.209 mmol, 1.2 eq.), stirring overnight. After confirming that the reaction was finished *via* TLC, the product was quenched with 10× volume of water, and it was worked up with 3 × 30 mL EA. The organic phase was combined and washed with 50 mL water, 50 mL brine, and subsequently dried over MgSO_4_. The crude was purified using flash column chromatography (0–5% MeOH in EA). *R*_f_ = 0.26 (TLC: EA/MeOH = 9/1). 0.116 g product was gained (yield: 67.1%). ^1^H NMR (400 MHz, MeOD-d_4_): *δ* 1.21–1.46 (m, 8H), *δ* 1.54–1.81 (m, 6H), *δ* 1.82–2.10 (m, 7H), *δ* 2.10–2.50 (m, 6H), *δ* 2.50–2.55 (m, 2H), *δ* 2.62–2.89 (m, 2H), *δ* 3.33–3.49 (m, 3H), *δ* 3.55–3.64 (m, 1H), *δ* 3.70–3.84 (m, 6H), *δ* 3.98–4.07 (m, 2H), *δ* 4.26–4.40 (m, 1H), *δ* 4.46–4.73 (m, 2H), *δ* 4.97–5.14 (m, 2H), *δ* 5.66–5.68 (d, 1H, *J* = 6.77 Hz), *δ* 5.79–5.81 (d, 1H, *J* = 8.96 Hz), *δ* 6.56–6.70 (m, 1H), *δ* 6.83 (m, 1H), *δ* 7.12–7.19 (m,2H), *δ* 7.33–7.63 (m, 3H), *δ* 7.70–7.75 (m, 1H). LCMS-ESI (method E) (*m*/*z*): C_52_H_63_N_7_O_13_ (944.11) [M^+^] 944.4; retention time 7.41 min.

#### 6-((2-(2,6-Dioxopiperidin-3-yl)-1-oxoisoindolin-4-yl)amino)-*N*-(3-methoxy-4-(4-(((*S*)-7-methoxy-5-oxo-2,3,5,11*a*-tetrahydro-1*H*-benzo[*e*]pyrrolo[1,2-*a*][1,4]diazepin-8-yl)oxy)butanamido)phenyl)hexanamide (15c, **JP-179-P12**)

14c (0.116 g, 0.117 mmol, 1 eq.) was dissolved in 5 mL DCM, added with palladium tetrakis[triphenylphosphine] (0.0068 g, 0.006 mmol, 0.05 eq.), triphenylphosphine (0.0077 g, 0.029 mmol, 0.25 eq.), and pyrrolidine (0.01 mL, 0.140 mmol, 1.2 eq.). The mixture was stirred at room temperature for 2 h. The mixture was then evaporated and dried in a rotary evaporator, which was purified using flash column chromatography (0–20% MeOH in EA). *R*_f_ = 0.34 (TLC: EA/MeOH = 8/2). 0.096 g product was retrieved (yield > 99.9%). ^1^H NMR (400 MHz, DMSO-d_6_): *δ* 1.36–1.49 (m, 2H), *δ* 1.59–1.68 (m, 4H), *δ* 1.80–1.95 (m, 2H), *δ* 1.95–2.10 (m, 5H), *δ* 2.15–2.38 (m, 5H), *δ* 2.51–2.61 (m, 2H), *δ* 3.06–3.16 (m, 2H), *δ* 3.40–3.49 (m, 2H), *δ* 3.55–3.61 (m, 1H), *δ* 3.67 (s, 3H), *δ* 3.76 (s, 3H), *δ* 3.91–4.06 (m, 2H), *δ* 4.22–4.52 (m, 2H), *δ* 4.64–4.81 (dd, 1H, *J* = 10.27,4.73 Hz), *δ* 5.67 (s, 1H), *δ* 6.12 (s, 1H), *δ* 6.39 (s, 1H), *δ* 6.68–6.78 (m, 1H), *δ* 6.86–6.92 (m, 1H), *δ* 7.06–7.08 (d, 1H, *J* = 7.48 Hz), *δ* 7.24–7.27 (m, 2H), *δ* 7.46 (s, 1H), *δ* 7.57 (s, 1H), *δ* 7.73–7.75 (d, 1H, *J* = 8.35 Hz), *δ* 9.07 (s, 1H), *δ* 9.93 (s, 1H). ^13^C NMR (100 MHz, DMSO-d_6_): *δ* 22.88, 25.25, 25.49, 26.89, 28.85, 29.54, 30.72, 30.85, 31.67, 32.74, 36.89, 43.12, 45.96, 51.78, 53.59, 54.21, 54.69, 55.92, 56.29, 56.39, 58.59, 58.86, 95.26, 103.16, 110.22, 110.93, 113.03, 115.57, 122.81, 123.31, 127.24, 129.47, 136.88, 139.82, 141.17, 144.23, 152.11, 165.29, 167.38, 171.01, 171.56, 172.42, 173.00. LCMS-ESI (method E) (*m*/*z*): C_43_H_49_N_7_O_9_ (807.37) [M − H^+^] 806.3; racemic structure peaks of the final product observed at 6.27 and 6.35 min; purity 90.63%. LCMS-ESI (method E) (*m*/*z*): peak at 2.80 min, purity 96.2%. HRMS-ESI (*m*/*z*): C_43_H_49_N_7_O_9_ (807.37) [M + H^+^] calculated 808.36645; found 808.3655; error −1.20 ppm.

#### Allyl (3-fluoro-4-nitrophenyl)carbamate (17a, **JP-193-09**)

3-Fluoro-4 nitroaniline (16a, 0.100 g, 0.641 mmol, 1 eq.) was added into 2 mL THF, then added with K_2_CO_3_ (0.106 g, 0.769 mmol, 1.2 eq.). Allyl chloroformate (0.08 mL, 0.705 mmol, 1.1 eq.) was then added dropwise, and the mixture was stirred overnight. The reaction was quenched with 10 mL DCM and washed with 10 mL CuSO_4_, 3 × 10 mL sat. Na_2_CO_3_, and dried over Na_2_SO_4_. The organic layer was evaporated in a rotary evaporator and then purified in flash chromatography (0–10% EA in hexane). *R*_f_ = 0.58 (TLC: EA/hexane = 2/8). The product was finally obtained at 0.112 g (yield: 72.7%). ^1^H NMR (400 MHz, CDCl_3_): *δ* 4.70–4.71 (d, 2H, *J* = 5.84 Hz), *δ* 5.30–5.32 (d, 1H, *J* = 10.40 Hz), *δ* 5.36–5.41 (dd, 1H, *J* = 1.26, 17.19 Hz), *δ* 5.91–6.01 (m, 1H), *δ* 7.06 (s, 1H), *δ* 7.11–7.14 (d, 1H, *J* = 9.09 Hz), *δ* 7.61–7.64 (d, 1H, *J* = 13.16 Hz), *δ* 8.05–8.09 (t, 1H, *J* = 8.66 Hz). ^13^C NMR (100 MHz, CDCl_3_): *δ* 66.77, 106.86–107.12, 113.06–113.09, 119.28, 127.46–127.48, 131.52, 144.73–144.84, 152.23, 155.62, 158.24. LCMS-ESI (method E) (*m*/*z*): C_10_H_9_FN_2_O_4_ 240.19 [M − H^+^] 239.0; retention time 7.66 min.

#### Allyl (4-amino-3-fluorophenyl)carbamate (18a, **JP-193-10**)

17a (0.104 g, 0.431 mmol, 1 eq.) was dissolved in 2 mL EtOH/H_2_O solvent mixture (ratio: 3/1), added with iron powder (0.144 g, 2.586 mmol, 6 eq.) and NH_4_Cl (0.207 g, 3.879 mmol, 9 eq.). The suspension was stirred at 80 °C for 2 h. Once the reaction was confirmed finished *via* TLC, the reaction was quenched by cooling the reaction to r.t., and the suspension was filtered *via* Celite. The filtered organic layer was evaporated in a rotary evaporator and redissolved in EA, washed with water, brine, and dried over MgSO_4_. The solvent was then filtered and evaporated in a rotary evaporator, fully dried to obtain 0.096 g product and it was directly used without further purification. ^1^H NMR (400 MHz, DMSO-d_6_): *δ* 4.56–4.69 (dt, 2H, *J* = 5.40, 1.38 Hz), *δ* 4.80 (s, 2H), *δ* 5.20–5.23 (dd, 1H, *J* = 10.46, 1.26 Hz), *δ* 5.31–5.36 (dd, 1H, *J* = 18.12, 1.40 Hz), *δ* 5.93–5.99 (m, 1H), *δ* 6.65–6.70 (m, 1H), *δ* 6.89–6.91 (d, 1H, *J* = 8.05 Hz), *δ* 7.16–7.19 (d, 1H, *J* = 12.87 Hz), *δ* 9.41 (s, 1H). ^13^C NMR (100 MHz, DMSO-d_6_): *δ* 64.93, 115.52, 116.65–116.71, 117.88, 128.94, 133.91, 149.38, 151.72, 153.75. LCMS-ESI (method E) (*m*/*z*): C_10_H_11_FN_2_O_2_ (211.21) [M + H^+^] 211.1; retention time 5.10 min (method E).

#### Allyl 8-(4-((4-(((allyloxy)carbonyl)amino)-2-fluorophenyl)amino)-4-oxobutoxy)-7-methoxy-5-oxo-11-((tetrahydro-2*H*-pyran-2-yl)oxy)-2,3,11,11*a*-tetrahydro-1*H*-benzo[e]pyrrolo[1,2-*a*][1,4]diazepine-10(5*H*)-carboxylate (19a, **JP-193-11**)

8 (0.090 g, 0.174 mmol, 1 eq.) was dissolved in 3 mL DMF, added with DMAP (0.085 g, 0.696 mmol, 4 eq.) and EDC (0.166 g, 0.870 mmol, 5 eq.). The mixture was stirred for 30 min, then added 18a and left to stir at r.t. overnight. The reaction was quenched with 10× volume H_2_O, and it was extracted with 3 × 30 mL EA, then the organic layer was combined with and washed with 100 mL 1 M citric acid, 100 mL water, 100 mL brine, and dried over MgSO_4_. The organic layer was evaporated in a rotary evaporator and purified using flash column chromatography (40–80% EA in hexane). *R*_f_ = 0.18 (TLC: Hex/EA = 4/6). The product was collected and evaporated in a rotary evaporator, followed by drying under high vac which obtained 0.122 g (yield: 98.4%). ^1^H NMR (400 MHz, MeOD-d_4_): *δ* 1.46–1.65 (m, 4H), *δ* 1.68–1.85 (m, 2H), *δ* 2.03–2.10 (m, 2H), *δ* 2.11–2.28 (m, 4H), *δ* 2.51–2.75 (m, 2H), *δ* 3.46–3.55 (m, 2H), *δ* 3.56–3.70 (m, 2H), *δ* 3.90 (s, 3H), *δ* 3.93–4.01 (m, 1H), *δ* 4.09–4.18 (m, 3H), *δ* 4.42–4.75 (m, 4H), *δ* 5.03–5.15 (m, 2H), *δ* 5.24–5.27 (d, 1H, *J* = 10.50 Hz), *δ* 5.36–5,41 (dd, 1H, *J* = 1.10, 17.22 Hz), *δ* 5.79–5.81 (m, 1H), *δ* 5.89–5.92 (d, 1H, *J* = 9.39 Hz), *δ* 5.96–6.06 (m, 1H), *δ* 6.89 (s, 1H), *δ* 6.98–7.03 (m, 1H), *δ* 7.10–7.12 (d, 1H, *J* = 8.73 Hz), *δ* 7.20–7.24 (m, 1H), *δ* 7.46–7.49 (d, 1H, *J* = 12.80 Hz), *δ* 7.68–7.72 (t, 1H, *J* = 7.83 Hz). LCMS-ESI (method E) (*m*/*z*): C_36_H_43_FN_4_O_10_ 710.76; retention time 7.78 min.

#### (*S*)-*N*-(4-Amino-2-fluorophenyl)-4-((7-methoxy-5-oxo-2,3,5,11*a*-tetrahydro-1*H*-benzo[*e*]pyrrolo[1,2-*a*][1,4]diazepin-8-yl)oxy)butanamide (20a, **JP-193-12**)

19a (0.106 g, 0.149 mmol, 1 eq.) was dissolved 5 mL DCM, added with PPh_3_ (0.0197 g, 0.075 mmol, 0.5 eq.), palladium tetrakis[triphenylphosphine] (0.017 g, 0.015 mmol, 0.1 eq.), and pyrrolidine (0.019 mL, 0.224 mmol, 1.5 eq.). The solvent was stirred for 2 h. The reaction was quenched *via* evaporation in a rotary evaporator and dried under high vac. The crude was further purified using flash column chromatography (0–3% MeOH in EA). *R*_f_ = 0.38 (TLC: EA/MeOH = 9/1) The product was collected, evaporated in a rotary evaporator, and dried under high vac. The compound was further purified *via* preparative column chromatography (method D). 0.020 g product was obtained (yield: 29.9%). ^1^H NMR (400 MHz, DMSO-d_6_): *δ* 1.94–2.05 (m, 4H), *δ* 2.45–2.47 (m, 2H), *δ* 2.10–2.34 (m, 2H), *δ* 2.46 (m, 2H) *δ* 3.17 (s, 3H), *δ* 3.64–3.67 (m, 2H), *δ* 3.74–3.82 (m, 1H), *δ* 3.92 (s, 2H), *δ* 5.25 (s, 2H), *δ* 6.33–6.37 (dd, 1H, *J* = 15.85, 13.23 Hz), *δ* 6.83 (s, 1H), *δ* 7.06–7.12 (m, 2H), *δ* 7.34 (s, 1H), *δ* 7.77–7.79 (d, 1H, *J* = 4.19 Hz), *δ* 9.25 (s, 1H). ^13^C NMR (100 MHz, DMSO-d_6_): *δ* 24.13, 25.12, 29.29, 32.25, 49.07, 53.89, 56.09, 56.30, 56.40, 100.65, 100.88, 109.70, 110.60, 111.73, 114.11, 120.25, 127.66, 141.06, 147.40, 150.67, 163.81, 164.73, 171.12. LCMS-ESI (method E) (*m*/*z*): C_23_H_26_FN_4_O_4_ (440.19) [M + H^+^] 441.1; retention time 4.49 min; purity: 85.4%. LCMS-ESI (method F) (*m*/*z*): retention time at 3.05 min, purity 92.5%. HRMS-ESI: C_23_H_25_FN_4_O_4_ (440.19) [M + H^+^] calculated 441.19326; found 441.1930; error −0.68 ppm.

#### Allyl (3-methyl-4-nitrophenyl)carbamate (17b, **JP-193-13**)

3-Methyl-4-nitroaniline (16b, 0.100 g, 0.657 mmol, 1 eq.) was dissolved in 2 mL DMF, which was added with K_2_CO_3_ (0.109 g, 0.788 mmol, 1.2 eq.) and 97% allyl chloroformate (0.11 mL, 0.723 mmol, 1.1 eq.). The mixture was allowed to stir at r.t. overnight. After the reaction was confirmed finished *via* TLC, it was quenched with 10 mL DCM, and it was washed with 10 sat. CuSO_4_, 3 × 10 mL water, 3 × 10 mL Na_2_CO_3_. The organic layer was then dried over sodium sulphate, filtered, and then evaporated *via* a rotary evaporator. The crude was purified using flash column chromatography (0–10% EA in hexane). *R*_f_ = 0.60 (TLC: hexane/EA = 8/2). The product was collected and evaporated in a rotary evaporator, gaining 0.130 g product after fully dried under high vac (yield: 83.7%). ^1^H NMR (400 MHz, CDCl_3_): *δ* 2.56 (s, 3H), *δ* 4.62–4.63 (d, 2H, *J* = 5.77 Hz), *δ* 5.22–5.24 (d, 1H, *J* = 10.41 Hz), *δ* 5.29–5.34 (dd, 1H, *J* = 1.31, 17.19 Hz), *δ* 5.86–5.93 (m, 1H), *δ* 6.85 (s, 1H), *δ* 7.28–7.33 (m, 2H), *δ* 7.97–8.00 (d, 1H, *J* = 8.89 Hz). ^13^C NMR (100 MHz, CDCl_3_): *δ* 21.43, 66.43, 115.78, 118.91, 121.07, 126.79, 131.84, 136.26, 142.29, 143.88, 152.57. LCMS-ESI (method E) (*m*/*z*): C_11_H_12_N_2_O_4_ (236.22) [M − H^+^] found 235.0; retention time 7.75 min.

#### Allyl (4-amino-3-methylphenyl)carbamate (18b, **JP-193-14**)

17b (0.080 g, 0.339 mmol, 1 eq.) was dissolved in 2 mL EtOH/H_2_O solvent mixture (ratio: 3/1), added with iron powder (0.114 g, 2.034 mmol, 6 eq.) and NH_4_Cl (0.163 g, 3.051 mmol, 9 eq.). The suspension was stirred at 80 °C for 2 h. Once the reaction was confirmed finished *via* TLC, the reaction was quenched by cooling the reaction to r.t., and the suspension was filtered *via* Celite. The filtered organic layer was evaporated in a rotary evaporator and redissolved in EA, washed with water, brine, and dried over MgSO_4_. The solvent was then filtered and evaporated in a rotary evaporator, fully dried to obtain 0.096 g product and it was directly used without further purification. ^1^H NMR (400 MHz, DMSO-d_4_): *δ* 2.01 (s, 3H), *δ* 4.53–4.55 (m, 4H), *δ* 5.19–5.22 (d, 1H, *J* = 10.40 Hz), *δ* 5.30–5,35 (d, 1H, *J* = 17.19 Hz), *δ* 5.91–6.00 (m, 1H), *δ* 6.50–6.52 (d, 1H, *J* = 8.38 Hz), *δ* 6.94–6.99 (m, 2H), *δ* 9.12 (s, 1H). LCMS-ESI (*m*/*z*): [M + H]^+^ calcd for C_11_H_14_N_2_O_2_ 207.25; found 207.1; retention time 4.03 min (method E).

#### Allyl 8-(4-((4-(((allyloxy)carbonyl)amino)-2-methylphenyl) amino)-4-oxobutoxy)-7-methoxy-5-oxo-11-((tetrahydro-2H-pyran-2-yl)oxy)-2,3,11,11*a*-tetrahydro-1*H*-benzo[*e*]pyrrolo[1,2-*a*][1,4]diazepine-10(5*H*)-carboxylate (19b, **JP-193-15**)

8 (0.090 g, 0.174 mmol, 1 eq.) was dissolved in 3 mL DMF, added with DMAP (0.083 g, 0.348 mmol, 2 eq.) and EDC (0.083 g, 0.248 mmol, 2.5 eq.). The mixture was stirred for 30 min, then added 18b and left to stir at r.t. overnight. The reaction was quenched with 10× volume H_2_O, and it was extracted with 3 × 30 mL EA, the organic layer was combined with and washed with 100 mL 1 M citric acid, 100 mL water, 100 mL brine, and dried over MgSO_4_. The organic layer was evaporated in a rotary evaporator and purified using flash column chromatography (0–70% EA in hexane). *R*_f_ = 0.26 (TLC: Hex/EA = 3/7). The product was collected and evaporated in a rotary evaporator, followed by high vac drying, yielding 0.119 g (yield: 96.0%). ^1^H NMR (400 MHz, MeOD-d_4_): *δ* 1.41–1.63 (m, 4H), *δ* 1.63–1.89 (m, 2H), *δ* 2.0–2.10 (m, 3H), *δ* 2.10–2.89 (m, 6H), *δ* 2.56–2.71 (m, 2H), *δ* 3.43–3.54 (m, 2H), *δ* 3.54–3.69 (m, 2H), *δ* 3.84–4.01 (m, 4H), *δ* 4.09–4.18 (m, 3H), *δ* 4.30–4.74 (m, 4H), *δ* 5.02–5.14 (m, 2H), *δ* 5.21–5.24 (d, 1H, *J* = 10.47 Hz), *δ* 5.33–5.38 (dd, 1H, *J* = 17.23, 1.36 Hz), *δ* 5.77–5.80 (m, 1H), *δ* 5.88–5.90 (d, 1H, *J* = 9.40 Hz), *δ* 5.94–6.04 (m, 1H), *δ* 6.96–6.98 (d, 1H, *J* = 7.96 Hz), *δ* 7.19–7.21 (m, 2H), *δ* 7.25–7.27 (d, 1H, *J* = 8.69 Hz), *δ* 7.31 (s, 1H). LCMS-ESI (method E) (*m*/*z*): C_37_H_46_N_4_O_10_ (706.79) [M + Na^+^] 729.3; retention time 7.68 min.

#### (*S*)-*N*-(4-Amino-2-methylphenyl)-4-((7-methoxy-5-oxo-2,3,5,11*a*-tetrahydro-1*H*-benzo[*e*]pyrrolo[1,2-*a*][1,4]diazepin-8-yl)oxy)butanamide (20b, **JP-193-16**)

19b (0.111 g, 0.157 mmol, 1 eq.) was dissolved 5 mL DCM, added with PPh_3_ (0.021 g, 0.079 mmol, 0.5 eq.), palladium tetrakis[triphenylphosphine] (0.019 g, 0.016 mmol, 0.1 eq), and pyrrolidine (0.020 mL, 0.236 mmol, 1.5 eq.). The solvent was stirred for 2 h. The reaction was quenched *via* evaporation in a rotary evaporator and dried under high vac. The crude was further purified using column chromatography (0–10% MeOH in EA). *R*_f_ = 0.36 (TLC: EA/MeOH = 8/2) The product was collected, evaporated in a rotary evaporator, and dried under high vac. 0.047 g of product was obtained (yield: 68.3%). ^1^H NMR (400 MHz, DMSO-d_6_): *δ* 1.88–1.95 (m, 2H), *δ* 2.01–2.05 (m, 5H), *δ* 2.18–2.27 (m, 2H), *δ* 2.41–2.45 (t, 2H, *J* = 7.05 Hz), *δ* 3.39–3.51 (m,2H), *δ* 3.57–3.62 (m, 1H), *δ* 3.67 (s, 3H), *δ* 3.93–3.98 (m, 2H), *δ* 4.88 (s, 2H), *δ* 6.08–6.17 (m, 1H), *δ* 6.37–6.39 (m, 1H), *δ* 6.84–6.87 (m, 1H), *δ* 7.06–7.10 (m, 1H), *δ* 7.26–7.35 (m, 1H), *δ* 7.78–7.79 (d, 1H, *J* = 4.42 Hz), *δ* 9.02 (m, 1H). ^13^C NMR (100 MHz, DMSO-d_6_): *δ* 18.46, 22.88–23.05, 24.14, 25.34–25.47, 32.40, 53.88–54.21, 56.09–56.34, 58.58–58.86, 67.85–68.35, 101.98, 110.05–110.63, 111.73–111.86, 115.39–115.67, 125.57, 127.26–127.46, 133.93, 138.77–139.74, 141.06–141.16, 146.92–147.42, 150.70, 152.05, 163.83, 164.72–165.28, 170.91. LCMS-ESI (method E) (*m*/*z*): C_24_H_29_N_4_O_4_ (436.51) [M + H^+^] 437.2; product peak retention time at 4.13 min, purity: 98.5%. LCMS-ESI (method F) (*m*/*z*): product peak retention time at 1.94 min, purity >98.5%. HRMS-ESI: C_24_H_28_N_4_O_4_ (436.51) [M + H^+^] calculated 437.21833; found 437.2179; error −1.03 ppm.

#### Allyl (3-methoxy-4-nitrophenyl)carbamate (17c, **JP-193-18**)

3-Methoxy-4-nitroaniline (16c, 0.100 g, 0.595 mmol, 1 eq.) was dissolved in 2 mL DMF, which was added with K_2_CO_3_ (0.099 g, 0.714 mmol, 1.2 eq.) and 97% allyl chloroformate (0.07 mL, 0.655 mmol, 1.1 eq.). The mixture was allowed to stir at r.t. overnight. After the reaction was finished (confirmed by TLC), it was quenched with 10 mL DCM, and it was washed with 10 sat. CuSO_4_, 3 × 10 mL water, 3 × 10 mL Na_2_CO_3_. The organic layer was then dried over sodium sulphate, filtered, and then evaporated *via* a rotary evaporator. The crude was purified using flash column chromatography (0–20% EA in hexane). *R*_f_ = 0.60 (TLC: hexane/EA = 6/4). The product was collected and evaporated in a rotary evaporator, gaining 0.129 g product after fully dried under high vac (yield: 85.6%). ^1^H NMR (400 MHz, CDCl_3_): *δ* 2.97 (s, 3H), *δ* 4.68–4.70 (d, 2H, *J* = 5.76 Hz), *δ* 5.28–5.40 (m, 2H), *δ* 5.91–6.00 (m, 1H), *δ* 6.73–6.75 (dd, 1H, *J* = 2.08, 8.92 Hz), *δ* 6.95 (s, 1H), *δ* 7.58 (d, 1H, *J* = 1.21 Hz), *δ* 7.92–7.95 (d, 1H, *J* = 8.91 Hz), ^13^C NMR (100 MHz, CDCl_3_): *δ* 56.56, 66.46, 102.42, 109.12, 118.97, 127.62, 131.74, 134.14, 143.97, 152.56, 155.17. LCMS-ESI (method E) (*m*/*z*): C_11_H_12_N_2_O_5_ (252.23) [M − H^+^] found 251.1; retention time 7.37 min.

#### Allyl (4-amino-3-methoxyphenyl)carbamate (18c, **JP-163-19**)

17c (0.090 g, 0.358 mmol, 1 eq.) was dissolved in 2 mL EtOH/H_2_O solvent mixture (ratio: 3/1), added with iron powder (0.120 g, 2.148 mmol, 6 eq.) and NH_4_Cl (0.172 g, 3.222 mmol, 9 eq.). The suspension was stirred at 80 °C for 2 h. Once the reaction was confirmed finished using TLC, the reaction was quenched by cooling to r.t., and the suspension was filtered *via* Celite. The filtered organic layer was evaporated in a rotary evaporator and redissolved in EA, washed with water, brine, and dried over MgSO_4_. The solvent was then filtered and evaporated in a rotary evaporator, fully dried to obtain 0.066 g product, which was directly used without further purification. ^1^H NMR (400 MHz, DMSO-d_6_): *δ* 3.71 (s, 3H), *δ* 4.42 (s, 2H), *δ* 4.55–4.56 (d, 2H, *J* = 5.39 Hz), *δ* 5.20–5.23 (dd, 1H, *J* = 10.46,1.32 Hz), *δ* 5.31–5.36 (dd, 1H, *J* = 17.22, 1.42 Hz), *δ* 5.91–6.01 (m, 1H), *δ* 6.52–6,54 (d, 1H, *J* = 8.32 Hz), *δ* 6.71–6.73 (d, 1H, *J* = 7.85 Hz), *δ* 7.01 (s, 1H), *δ* 9.23 (s, 1H). ^13^C NMR (100 MHz, DMSO-d_6_): *δ* 55.63, 64.74, 103.59, 112.01, 114.08, 117.70, 129.21, 133.53, 134.08, 146.66, 153.85, 212.05. LCMS-ESI (method E) (*m*/*z*): C_11_H_14_N_2_O_3_ (222.24) [M + H^+^] 223.1; retention time 4.01 min.

#### Allyl 8-(4-((4-(((allyloxy)carbonyl)amino)-2-methoxyphenyl) amino)-4-oxobutoxy)-7-methoxy-5-oxo-11-((tetrahydro-2*H*-pyran-2-yl)oxy)-2,3,11,11*a*-tetrahydro-1*H*-benzo[*e*]pyrrolo[1,2-*a*][1,4]diazepine-10(5*H*)-carboxylate (19c, **JP-193-20**)

8 (0.090 g, 0.174 mmol, 1 eq.) was dissolved in 3 mL DMF, added with DMAP (0.043 g, 0.348 mmol, 2 eq.) and EDC (0.083 g, 0.435 mmol, 2.5 eq.). The mixture was stirred for 30 min, then added 18c and left to stir at r.t. overnight. The reaction was quenched with 10× volume H_2_O, and it was extracted with 3 × 30 mL EA, the organic layer was combined with and washed with 100 mL 1 M citric acid, 100 mL water, 100 mL brine, and dried over MgSO_4_. The organic layer was evaporated in a rotary evaporator and purified using flash column chromatography (0–100% EA in hexane). *R*_f_ = 0.22 (TLC: Hex/EA = 4/6). The product was collected and evaporated in a rotary evaporator, followed by drying under high vac, which obtained 0.135 g (yield > 99.9%). ^1^H NMR (400 MHz, MeOD-d_4_): *δ* 1.45–1.60 (m, 4H), *δ* 1.70–1.79 (m, 2H), *δ* 2.01–2.08 (m, 2H), *δ* 2.09–2.23 (m, 4H), *δ* 2.54–2.69 (m, 2H), *δ* 3.42–3.53 (m, 2H), *δ* 3.53–3.69 (m, 2H), *δ* 3.79–3.80 (d, 3H, *J* = 3.42 Hz), *δ* 3.87 (s, 3H), *δ* 3.89–3.98 (m, 1H), *δ* 4.07–4.13 (q, 3H, *J* = 7.11 Hz), *δ* 4.39–4.64 (m, 4H), *δ* 5.05–5.08 (d, 2H, *J* = 14.47 Hz), *δ* 5.21–5.24 (d, 1H, *J* = 10.52 Hz), *δ* 5.34–5.38 (d, 1H, *J* = 17.26 Hz), *δ* 5.76–5.79 (d, 1H, *J* = 9.64 Hz), *δ* 5.87–5.89 (d, 1H, *J* = 9.32 Hz), *δ* 5.95–6.04 (m, 1H), *δ* 6.86–6.89 (m, 2H), *δ* 6.96 (s, 1H), *δ* 7.18–7.20 (d, 1H, *J* = 7.76 Hz), *δ* 7.29 (s, 1H), *δ* 7.78–7.80 (d, 1H, *J* = 8.68 Hz). LCMS-ESI (method E) (*m*/*z*): C_37_H_46_N_4_O_11_ (722.8) [M + Na^+^] 745.3; retention time 7.80 min.

#### (*S*)-*N*-(4-Amino-2-methoxyphenyl)-4-((7-methoxy-5-oxo-2,3,5,11*a*-tetrahydro-1*H*-benzo[*e*]pyrrolo[1,2-*a*][1,4]diazepin-8-yl)oxy)butanamide (20c, **JP-193-21**)

19c (0.124 g, 0.172 mmol, 1 eq.) was dissolved 5 mL DCM, added with PPh_3_ (0.023 g, 0.086 mmol, 0.5 eq.), palladium tetrakis[triphenylphosphine] (0.020 g, 0.017 mmol, 0.1 eq.), and pyrrolidine (0.022 mL, 0.258 mmol, 1.5 eq.). The solvent was stirred for 2 h. The reaction was quenched *via* evaporation in a rotary evaporator and dried under high vac. The crude was further purified using flash column chromatography (0–10% MeOH in EA). *R*_f_ = 0.42 (TLC: EA/MeOH = 8/2) The product was collected, evaporated in a rotary evaporator, and dried under high vac. 0.050 g product was obtained (yield: 64.8%). ^1^H NMR (400 MHz, DMSO-d_6_): *δ* 1.76–1.90 (m, 2H), *δ* 1.98–2.03 (m, 2H), *δ* 2.18–2.31 (m, 2H), *δ* 2.43–2.46 (t, 2H, *J* = 7.36 Hz), *δ* 3.39–3.42 (m, 2H), *δ* 3.57–3.63 (m, 1H) *δ* 3.68 (s, 3H), *δ* 3.83 (s, 3H), *δ* 3.91 (s 2H), *δ* 4.10–4.13 (m, 2H), *δ* 6.08–6.11 (dd, 1H, J = 8.27, 2.18 Hz), *δ* 6.23–6.26 (m, 1H), *δ* 6.84 (s, 1H), *δ* 7.24–7.26 (d, 1H, *J* = 8.31 Hz), *δ* 7.34 (s, 1H), *δ* 7.78–7.79 (d, 1H, *J* = 4.40 Hz), *δ* 8.88 (m, 1H). ^13^C NMR (100 MHz, DMSO-d_6_): *δ* 22.56, 24.14, 25.27, 29.16–29.46, 31.75, 32.57, 49.07, 55.55–55.73, 56.09–56.30, 68.29, 98.19, 105.64, 110.61, 111.74, 116.35, 120.25, 125.74, 141.07, 147.41, 150.70, 163.83, 164.72, 170.63. LCMS-ESI (method E) (*m*/*z*): C_24_H_29_N_4_O_5_ (452.21) [M + H^+^] 453.2; retention time 4.21 min; purity: 92.5%. LCMS-ESI (method F) (*m*/*z*): retention time at 1.98 min, purity >98.5%. HRMS-ESI: C_24_H_29_N_4_O_5_ (452.21) [M + H^+^] calculated 453.21325; found 453.2129; error −0.69 ppm.

### Cell culture

CLL cells, the CLL cell line, MEC-1, the breast cancer cell line, MDA-MB-231 and the lenalidomide resistant myeloma cell line, RPMI-8226 were selected for testing the cytotoxicity of the PROTAC molecules. Primary CLL cells were collected from patients and normal B- and T-lymphocytes were obtained from healthy volunteers. All experiments were performed in accordance with the U.K. Human Tissue Authority guidelines, and experiments were approved by the local research ethics committee (17/SW/0263). Informed consent was obtained from all human participants in this study. Cell lines were acquired from DSMZ (MEC-1 and RPMI-8226) or ATCC (MDA-MB-231); cells were maintained in RPMI 1640 media (primary CLL cells, MEC-1 and RPMI-8226) or DMEM media (MDA-MB-231) with the addition of 10% FBS, 1% l-glutamine, and 1% penicillin streptomycin. The cells were seeded 500 000 cells per mL at 37 °C in a humidified atmosphere containing 5% CO_2_. The cells were split every 48 hours; cell count and viability were measured each time.

### Cell counting

10 μL of cell suspension was mixed with 10 μL of trypan blue. Subsequently, 10 μL of the mixture was pipetted into a cell counting slide. The slide was inserted into a Countess 3 cell counter (ThermoFisher Scientific) to quantify the cell count and cell viability.

### Apoptotic assay for RelA/p65-targeting PROTACs

500 000 cells per well were aliquoted into 24-well plates following resuspension with 1 mL of appropriate medium. All test compounds were dissolved in DMSO as 1 mM stock solution. Subsequently, working stocks of the PROTACs and their individual constituent molecules were produced by serial dilutions in a 96-well plate: RelA/p65-targeting PROTACs (1 μM, 0.5 μM and 0.25 μM, 0.125 μM, and 0.0625 μM). 10 μL of each dilution was then transferred to the cell suspensions in the 24-well plate. The plates were then incubated for 48 hours at 37 °C with 5% CO_2_. Samples were then harvested into 1.5 mL Eppendorf tubes and centrifuged at 500 × *g* for 5 min. The supernatant was then poured off and the cell pellets resuspended in 96 μL annexin V binding buffer and 4 μL FITC annexin V (both Biolegend) was added to each tube. The tubes were then incubated in the dark for 10 min prior to the addition of 4 μL 7-AAD. Finally, the cells were analysed using a CytoFLEX LX (Beckman Coulter) flow cytometer. In all cases, 10 000 events were recorded. Apoptosis was quantified using CytExpert software, while the percentage of apoptotic cells was defined as annexin-V positive and 7-AAD positive, or annexin-V positive and 7-AAD negative. The data was further analysed using GraphPad prism to calculate the LC_50_ values for each compound using non-linear regression analysis.

### Evaluation of NF-κB subunit expression following treatment with PROTAC

Aliquots of 0.5 × 10^6^ MEC-1 cells were treated with increasing concentrations of 15d for 24 h. Cells were harvested by centrifugation, fixed using Cyto-Fast™ fix/perm buffer set (Biolegend) for 20 min at 37 °C. Cells were then washed in Cyto-Fast™ Perm Wash solution and centrifuged at 300 × *g* for 5 min before being resuspended in 100 μL Perm Wash solution followed by the addition of 5 μL APC-labelled RelA/p65 antibody (Biolegend), 5 μL PE-labelled cRel antibody (eBiosciences) and 5 μL corallite 488-labelled RelB antibody (ThermoFisher). Cells were incubated for 20 min prior to washing in cell staining buffer, centrifugation at 300 × *g* for 5 min and resuspension in 100 μL cell staining buffer prior to acquisition of the data on a CytoFLEX LX flow cytometer.

### MG-132 proteasome inhibition assay

To determine whether the toxicity of 15d was dependent on proteasome activity, MDA-MB-231 cells were treated with increasing concentration of the proteasome inhibitor, MG-132 (0.1–10 μM) for 48 h. Aliquots of cells were first assessed for their apoptotic response to MG-132 using annexin V and 7-AAD labelling (as described above). In parallel, proteasome activity was assessed using a proteasome activity assay kit (Abcam). The kit uses an AMC-tagged peptide substrate (proteasome substrate (Succ-LLVY-AMC in DMSO), which releases free, highly fluorescent AMC (Ex/Em 350/440 nm) in the presence of proteolytic activity. MEC-1 cells were very sensitive to the cytotoxic effects of MG-132. So, in this cell line, combination studies were carried out with 0.18 μM MG-132. Treatment of MEC-1 cells with 0.18 μM MG-132 caused >40% reduction in proteasome activity without significant effects on cellular viability. Subsequently, 10 μL MG-132 stock was added to the 500 000 cells per mL MEC-1 cell with increasing concentration of 15d or the PBD building block, 20d. All treated cells were cultured for 48 h at 37 °C with 5% CO_2_. The cells were then harvested by centrifugation (300 × *g* for 5 min) and then incubated with annexin V and 7-AAD, prior to analysis by flow cytometry (as described above.).

### FRET melting assay

The single-strand oligonucleotide FRET hairpin was purchased from Eurogentec Ltd, tagged with TAM at 5′ and TAMRA at 3′ terminal (sequence: 5′-FAM-TAT-AAG-ATA-TAT-ATA-TTT-TTT-TAT-ATA-TAT-CTT-ATA-TAMRA-3′). Nuclease-free water was added to prepare 20 μM ssDNA stock solution, and it was further diluted to 400 nM using 50 mM K cacodylate buffer (pH = 7.4). The prepared ssDNA sample was annealed at 85 °C for 5 min and then allowed to cool down to room temperature and then stored at −20 °C for completing the annealing process. PBD controls and PROTACs were prepared as 20 μM working solutions diluted with 50 mM K cacodylate buffer (pH = 7.4). 25 μL compound working solution was added to 25 μl DNA stock in the well of the Bio-Rad 96-well plate. DNA Engine Opticom was used for melting. The sample was initially incubated at 30 °C for 3 h and then the temperature was gradually increased to 100 °C. The fluorescence signal was detected at intervals of 0.5 °C. The mean of the melting point was analysed *via* GraphPad prism, and the melting point difference between the sample and naked ssDNA (Δ*T*_m_) was calculated for comparison.

### Data analysis

All biological data was calculated and plotted in GraphPad Prism. The standard deviations were presented as error bars in the plotted graph. The sigmoid dose–response curves were plotted using non-linear regression (4 parameters) to obtain LC_50_ values (the concentration of drug required to kill 50% of the cells in culture). As for significance testing, the mean values in the two groups were measured and compared. The data were initially subjected to normality testing using the Shapiro–Wilk and Kolmogorov–Smirnov test. If the data passed the normality test, it was then further evaluated using a paired *t*-test to assess whether there was a significance in the data before and after treatment. It was considered significantly different when the *p* value of the testing groups <0.05 with 95% confidence interval. If the data failed the normality test, they were subsequently evaluated using the Kruskal–Wallis test with Dunn's multiple comparison *post hoc* correction, if more than one set of pairs were analysed.

## Abbreviations

7-AAD: 7-Aminoactinomycin D
BAIB: Bis(acetoxy)iodobenzene
CRBN: Cereblon
DCM: Dichloromethane
dd: Double of doublets
DHP: Dihydropyran
DMAP: 4-Dimethylaminopyridine
DMF: Dimethylformamide
DMSO: Dimethyl sulfoxide
EA: Ethyl acetate
EDC: 1-Ethyl-3-(3-dimethyl aminopropyl)carbodiimide
FBS: Fetal bovine serum
FITC: Fluorescein isothiocyanate
FRET: Fluorescence resonance energy transfer
G: Guanine
HPLC: High performance liquid chromatography
HRMS: High resolution mass spectrometry
ImiDs: Immunomodulatory drugs
LC_50_: Concentration of the toxic substance lethal to half of test cells
LC-MS: Liquid chromatography-mass spectrometry
Lys: Lysine
m: multiplet
M: Molar
NF-κB: Nuclear transcription kappa B
nM: Nanomolar
NMP: *N*-Methyl-2-pyrrolidone
NMR: Nuclear magnetic resonance
PBD: Pyrrolobenzodiazepine
PBS: Phosphate-buffered saline
PROTAC: Proteolysis targeting chimera
q: Quartet
RPMI: Roswell Park Memorial Institute
s: Singlet
SAR: Structure–activity relationships
ssDNA: Single-stranded DNA
T: Thymine
t: Triplet
TAMRA: Carboxytetramethylrhodamine
TEMPO: (2,2,6,6-Tetramethylpiperidin-1-yl)oxyl
TF: Transcription factor
TFA: Trifluoroacetic acid
THF: Tetrahydrofuran
TLC: Thin-layer chromatography
TPD: Targeted protein degradation
UV: Ultraviolet
Δ*T*_m_: Variation of the melting temperature
*m*/*z*: Mass-to-charge ratio

## Data availability

The data supporting this article have been included as part of the ESI.†

## Author contributions

K. M. R. and C. P. contributed equally. P. J., M. M. H., A. G. S. P., S. M., K. M. R., and C. P. designed the experiments and analysed the data. P. J., and M. M. H. designed, synthesized, and analysed the compounds. P. J., A. G. S. P., and C. P. performed the biological experiments. P. J., M. M. H., and K. M. R. performed the docking studies. P. J., K. M. R., and C. P. wrote the manuscript, with edits from M. M. H., A. G. S. P., and S. M.

## Conflicts of interest

The authors have no relevant conflicts of interest.

## Supplementary Material

MD-OLF-D5MD00316D-s001

MD-OLF-D5MD00316D-s002

MD-OLF-D5MD00316D-s003

MD-OLF-D5MD00316D-s004

MD-OLF-D5MD00316D-s005

MD-OLF-D5MD00316D-s006

MD-OLF-D5MD00316D-s007

MD-OLF-D5MD00316D-s008

MD-OLF-D5MD00316D-s009

MD-OLF-D5MD00316D-s010

MD-OLF-D5MD00316D-s011

MD-OLF-D5MD00316D-s012

MD-OLF-D5MD00316D-s013

MD-OLF-D5MD00316D-s014

MD-OLF-D5MD00316D-s015

MD-OLF-D5MD00316D-s016

MD-OLF-D5MD00316D-s017

MD-OLF-D5MD00316D-s018

MD-OLF-D5MD00316D-s019

MD-OLF-D5MD00316D-s020

MD-OLF-D5MD00316D-s021

MD-OLF-D5MD00316D-s022

MD-OLF-D5MD00316D-s023

## References

[cit1] Liu T., Zhang L., Joo D., Sun S.-C. (2017). NF-κB Signaling in Inflammation. Signal Transduction Targeted Ther..

[cit2] Taniguchi K., Karin M. (2018). NF-KB, Inflammation, Immunity and Cancer: Coming of Age. Nat. Rev. Immunol..

[cit3] Duran C. L., Karagiannis G. S., Chen X., Sharma V. P., Entenberg D., Condeelis J. S., Oktay M. H. (2023). Cooperative NF-KB and Notch1 Signaling Promotes Macrophage-Mediated MenaINV Expression in Breast Cancer. Breast Cancer Res..

[cit4] Hewamana S., Alghazal S., Lin T. T., Clement M., Jenkins C., Guzman M. L., Jordan C. T., Neelakantan S., Crooks P. A., Burnett A. K., Pratt G., Fegan C., Rowntree C., Brennan P., Pepper C. (2008). The NF-KB Subunit Rel A Is Associated with in Vitro Survival and Clinical Disease Progression in Chronic Lymphocytic Leukemia and Represents a Promising Therapeutic Target. Blood.

[cit5] Verzella D., Pescatore A., Capece D., Vecchiotti D., Ursini M. V., Franzoso G., Alesse E., Zazzeroni F. (2020). Life, Death, and Autophagy in Cancer: NF-KB Turns up Everywhere. Cell Death Dis..

[cit6] Bhagwat A. S., Vakoc C. R. (2015). Targeting Transcription Factors in Cancer. Trends Cancer.

[cit7] Li K., Crews C. M. (2022). PROTACs: Past, Present and Future. Chem. Soc. Rev..

[cit8] Zhuang J., Liu Q., Wu D., Tie L. (2022). Current Strategies and Progress for Targeting the “Undruggable” Transcription Factors. Acta Pharmacol. Sin..

[cit9] Horie K., Ma J., Umezawa K. (2015). Inhibition of Canonical NF-KB Nuclear Localization by (−)-DHMEQ via Impairment of DNA Binding. Oncol. Res..

[cit10] Shono Y., Tuckett A. Z., Ouk S., Liou H.-C., Altan-Bonnet G., Tsai J. J., Oyler J. E., Smith O. M., West M. L., Singer N. V., Doubrovina E., Pankov D., Undhad C. V., Murphy G. F., Lezcano C., Liu C., O'Reilly R. J., van den Brink M. R. M., Zakrzewski J. L. (2014). A Small-Molecule c-Rel Inhibitor Reduces Alloactivation of T Cells without Compromising Antitumor Activity. Cancer Discovery.

[cit11] Thurston D. E. and Pysz I., Chemistry and Pharmacology of Anticancer Drugs, CRC Press, Boca Raton, 2nd edn, 2021

[cit12] Corcoran D. B., Lewis T., Nahar K. S., Jamshidi S., Fegan C., Pepper C., Thurston D. E., Rahman K. M. (2019). Effects of Systematic Shortening of Noncovalent C8 Side Chain on the Cytotoxicity and NF-KB Inhibitory Capacity of Pyrrolobenzodiazepines (PBDs). J. Med. Chem..

[cit13] Hu W.-P., Tsai F.-Y., Yu H.-S., Sung P.-J., Chang L.-S., Wang J.-J. (2008). Induction of Apoptosis by DC-81-Indole Conjugate Agent Through NF-KB and JNK/AP-1 Pathway. Chem. Res. Toxicol..

[cit14] Rahman K. M., Jackson P. J. M., James C. H., Basu B. P., Hartley J. A., de la Fuente M., Schatzlein A., Robson M., Pedley R. B., Pepper C., Fox K. R., Howard P. W., Thurston D. E. (2013). GC-Targeted C8-Linked Pyrrolobenzodiazepine–Biaryl Conjugates with Femtomolar in Vitro Cytotoxicity and
in Vivo Antitumor Activity in Mouse Models. J. Med. Chem..

[cit15] Kanzaki H., Chatterjee A., Hossein H., Zhang X., Chung S., Deng N., Ramanujan V. K., Cui X., Greene M. I., Murali R. (2021). Disabling the Nuclear Translocalization of RelA/NF-KB by a Small Molecule Inhibits Triple-Negative Breast Cancer Growth. Breast Cancer: Targets Ther..

[cit16] Zou Y., Ma D., Wang Y. (2019). The PROTAC Technology in Drug Development. Cell Biochem. Funct..

[cit17] Paiva S.-L., Crews C. M. (2019). Targeted Protein Degradation: Elements of PROTAC Design. Curr. Opin. Chem. Biol..

[cit18] Pettersson M., Crews C. M. (2019). PROteolysis TArgeting Chimeras (PROTACs) — Past, Present and Future. Drug Discovery Today: Technol..

[cit19] Bondeson D. P., Smith B. E., Burslem G. M., Buhimschi A. D., Hines J., Jaime-Figueroa S., Wang J., Hamman B. D., Ishchenko A., Crews C. M. (2018). Lessons in PROTAC Design from Selective Degradation with a Promiscuous Warhead. Cell Chem. Biol..

[cit20] Guenette R. G., Yang S. W., Min J., Pei B., Potts P. R. (2022). Target and Tissue Selectivity of PROTAC Degraders. Chem. Soc. Rev..

[cit21] Mannion J., Gifford V., Bellenie B., Fernando W., Ramos Garcia L., Wilson R., John S. W., Udainiya S., Patin E. C., Tiu C., Smith A., Goicoechea M., Craxton A., Moraes de Vasconcelos N., Guppy N., Cheung K. M. J., Cundy N. J., Pierrat O., Brennan A., Roumeliotis T. I., Benstead-Hume G., Alexander J., Muirhead G., Layzell S., Lyu W., Roulstone V., Allen M., Baldock H., Legrand A., Gabel F., Serrano-Aparicio N., Starling C., Guo H., Upton J., Gyrd-Hansen M., MacFarlane M., Seddon B., Raynaud F., Roxanis I., Harrington K., Haider S., Choudhary J. S., Hoelder S., Tenev T., Meier P. (2024). A RIPK1-Specific PROTAC Degrader Achieves Potent Antitumor Activity by Enhancing Immunogenic Cell Death. Immunity.

[cit22] Liu J., Chen H., Kaniskan H. Ü., Xie L., Chen X., Jin J., Wei W. (2021). TF-PROTACs Enable Targeted Degradation of Transcription Factors. J. Am. Chem. Soc..

[cit23] Surani Y. , Wand M., Picconi P., Di Palma M., Zenezini Chiozzi R., Hasan M., Maynard-Smith M., Steiner R., Rahman K., Hind C. and Sutton M., Convergent evolution of antibiotic resistance mechanisms between synthetic pyrrolobenzodiazepines (PBDs) and the naturally occurring albicidin in multidrug resistant *Klebsiella pneumoniae*, Research Square, 2024, preprint, 10.21203/rs.3.rs-4901630/v1 PMC12144233 40481275

[cit24] Zhou H., Bai L., Xu R., McEachern D., Chinnaswamy K., Li R., Wen B., Wang M., Yang C.-Y., Meagher J. L., Sun D., Stuckey J. A., Wang S. (2021). SD-91 as A Potent and Selective STAT3 Degrader Capable of Achieving Complete and Long-Lasting Tumor Regression. ACS Med. Chem. Lett..

[cit25] Patel U., Smalley J. P., Hodgkinson J. T. (2023). PROTAC Chemcal Probes for Histone Deacetylase Enzymes. RSC Chem. Biol..

[cit26] Qiu X., Sun N., Kong Y., Li Y., Yang X., Jiang B. (2019). Chemoselective Synthesis of Lenalidomide-Based PROTAC Library Using Alkylation Reaction. Org. Lett..

[cit27] Pettersson M., Crews C. M. (2019). PROteolysis TArgeting Chimeras (PROTACs) — Past, Present and Future. Drug Discovery Today:Technol..

[cit28] Riching K. M., Caine E. A., Urh M., Daniels D. L. (2022). The Importance of Cellular Degradation Kinetics for Understanding Mechanisms in Targeted Protein Degradation. Chem. Soc. Rev..

[cit29] Heider M., Eichner R., Stroh J., Morath V., Kuisl A., Zecha J., Lawatscheck J., Baek K., Garz A. K., Rudelius M., Deuschle F. C., Keller U., Lemeer S., Verbeek M., Götze K. S., Skerra A., Weber W. A., Buchner J., Schulman B. A., Kuster B., Fernández-Sáiz V., Bassermann F. (2021). The IMiD target CRBN determines HSP90 activity toward transmembrane proteins essential in multiple myeloma. Mol. Cell.

[cit30] Kassam F., Enright K., Dent R., Dranitsaris G., Myers J., Flynn C., Fralick M., Kumar R., Clemons M. (2009). Survival Outcomes for Patients with Metastatic Triple-Negative Breast Cancer: Implications for Clinical Practice and Trial Design. Clin. Breast Cancer.

[cit31] Espinoza-Sánchez N. A. , Enciso J., Pelayo R. and Fuentes-Pananá E. M., An NFKB-Dependent Mechanism of Tumor Cell Plasticity and Lateral Transmission of Aggressive Features, 2018, vol. 9 10.18632/oncotarget.25465 PMC6003573 29928478

[cit32] Winzker M., Friese A., Koch U., Janning P., Ziegler S., Waldmann H. (2020). Development of a PDE*δ*-Targeting PROTACs That Impair Lipid Metabolism. Angew. Chem..

[cit33] Zhang Y., Yang B., Zhao J., Li X., Zhang L., Zhai Z. (2018). Proteasome Inhibitor Carbobenzoxy-L-Leucyl-L-Leucyl-L-Leucinal (MG132) Enhances Therapeutic Effect of Paclitaxel on Breast Cancer by Inhibiting Nuclear Factor (NF)-KB Signaling. Med. Sci. Monit..

[cit34] Ferguson L., Bhakta S., Fox K. R., Wells G., Brucoli F. (2020). Synthesis and Biological Evaluation of a Novel C8-Pyrrolobenzodiazepine (PBD) Adenosine Conjugate. A Study on the Role of the PBD Ring in the Biological Activity of PBD-Conjugates. Molecules.

[cit35] Brosseau C., Colston K., Dalgleish A. G., Galustian C. (2012). The Immunomodulatory Drug Lenalidomide Restores a Vitamin D Sensitive Phenotype to the Vitamin D Resistant Breast Cancer Cell Line MDA-MB-231 through Inhibition of BCL-2: Potential for Breast Cancer Therapeutics. Apoptosis.

[cit36] Yin L., Wen X., Lai Q., Li J., Wang X. (2018). Lenalidomide Improvement of Cisplatin Antitumor Efficacy on Triple-Negative Breast Cancer Cells Inï¿½vitro. Oncol. Lett..

[cit37] Gribben J. G., Fowler N., Morschhauser F. (2015). Mechanisms of Action of Lenalidomide in B-Cell Non-Hodgkin Lymphoma. J. Clin. Oncol..

[cit38] Thomas B. ab I., Lewis H. L., Jones D. H., Ward S. E. (2023). Central Nervous System Targeted Protein Degraders. Biomolecules.

