# Supplementary material for: Design, synthesis and evaluation of pyrrolobenzodiazepine (PBD)-based PROTAC conjugates for the selective degradation of the NF-κB RelA/p65 subunit
Source: RSC Med Chem. 2025 May 8;16(9):4068–90. doi: 10.1039/d5md00316d (PMC12117510; doi:10.1039/d5md00316d)
Supplement: MD-016-D5MD00316D-s015 [file MD-016-D5MD00316D-s015.pdf]

Supplementary Information (SI) for RSC Medicinal Chemistry.
This journal is © The Royal Society of Chemistry 2025

Supporting Information

Design synthesis and evaluation of Pyrrolobenzodiazepine (PBD)-based PROTAC conjugates for the selective degradation of the NF-κB RelA/p65 subunit

Peiqin Jin[a], Md. Mahbub Hassan[a], Andrea G.S. Pepper[b], Simon Mitchell[b], Khondaker Miraz Rahman[a*], and Chris Pepper[b*].

[a]Institute of Pharmaceutical Science, School of Cancer and Pharmaceutical Sciences, King's College London, London SE1 9NH, U.K
[b]Brighton and Sussex Medical School, University of Sussex, Brighton BN1 9PX, U.K
*joint senior authors

Characterisation of synthesized final products

High-performance liquid chromatography-tandem mass spectrometry (LCMS) was applied to characterise products. The product analysis was carried out using an Agilent 1260 separating system using H_2O (solvent A) and acetonitrile (solvent B) as the mobile phase, while Monolithic C_{18} 50 × 4.6 mm LC column (Phenomenex) worked as stationary phase. The products were dissolved in a mixture of DMSO/Acetonitrile (1/4). Method E (10 min) and Method F (5 min) were used for analysis (Flow rate: 0.5 mL/min; inject volume: 10 µL), while samples were split and passed through an Agilent 6120 quadrupole mass spectrometer. Formic acid was added (0.1%) in both solvent A and B to maintain an acidic mobile phase condition.

LCMS Methods:

Method E:

Solvent A (95%) with Solvent B (5%) was maintained for 2 mins and then ramped up to 50% Solvent B in 3 mins. The gradient was retained for 1 min and then Solvent B was increased to 95% in 1.5 min. Solvent B was finally returned to 5% in 1.5 min and maintained for 1 min.

Time (min)	% Solvent A	% Solvent B
0	95	5
2	95	5
5	50	50
6	50	50
7.5	5	95
9	95	5
10	95	5

Method F:

Solvent A (95%) with solvent B (5%) was ramped up to 90% in 3 min, while solvent B was then ramped up to 95% within 0.5 min. The solvent gradient was kept for 1 min, and then solvent B was reduced to 5% within 0.5 min.

Time (min)	% Solvent A	% Solvent B
0	95	5
3	95	5
3.5	5	95
4.5	5	95
5	95	5

NMR and MS result of products:

tert-butyl 6-((2-(2,6-dioxopiperidin-3-yl)-1-oxoisoindolin-4-yl) amino) hexanoate (**1, JP-163-03**)

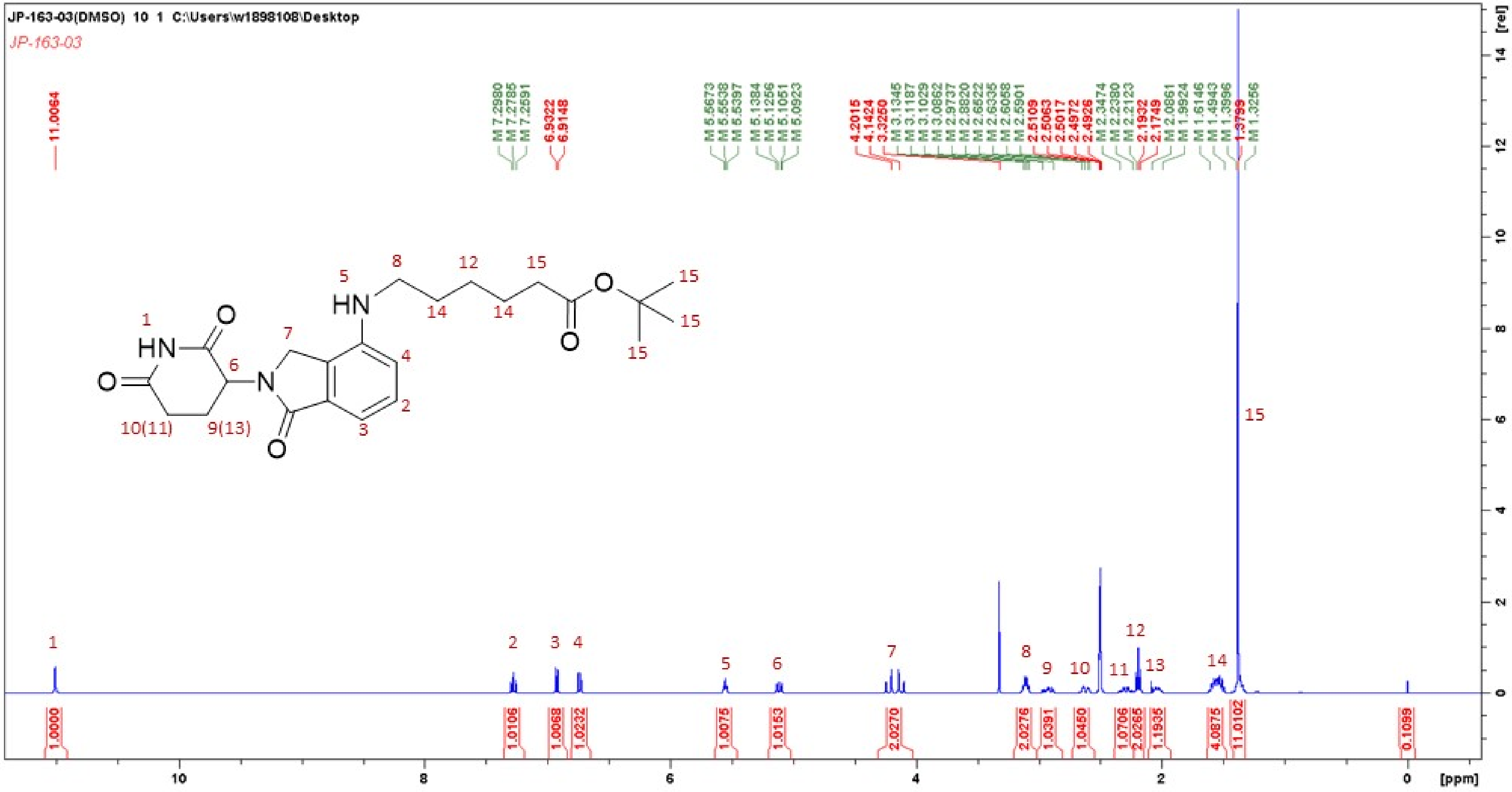

Figure S1-1. Proton ^{1}H NMR of **1** (**JP-163-03**)

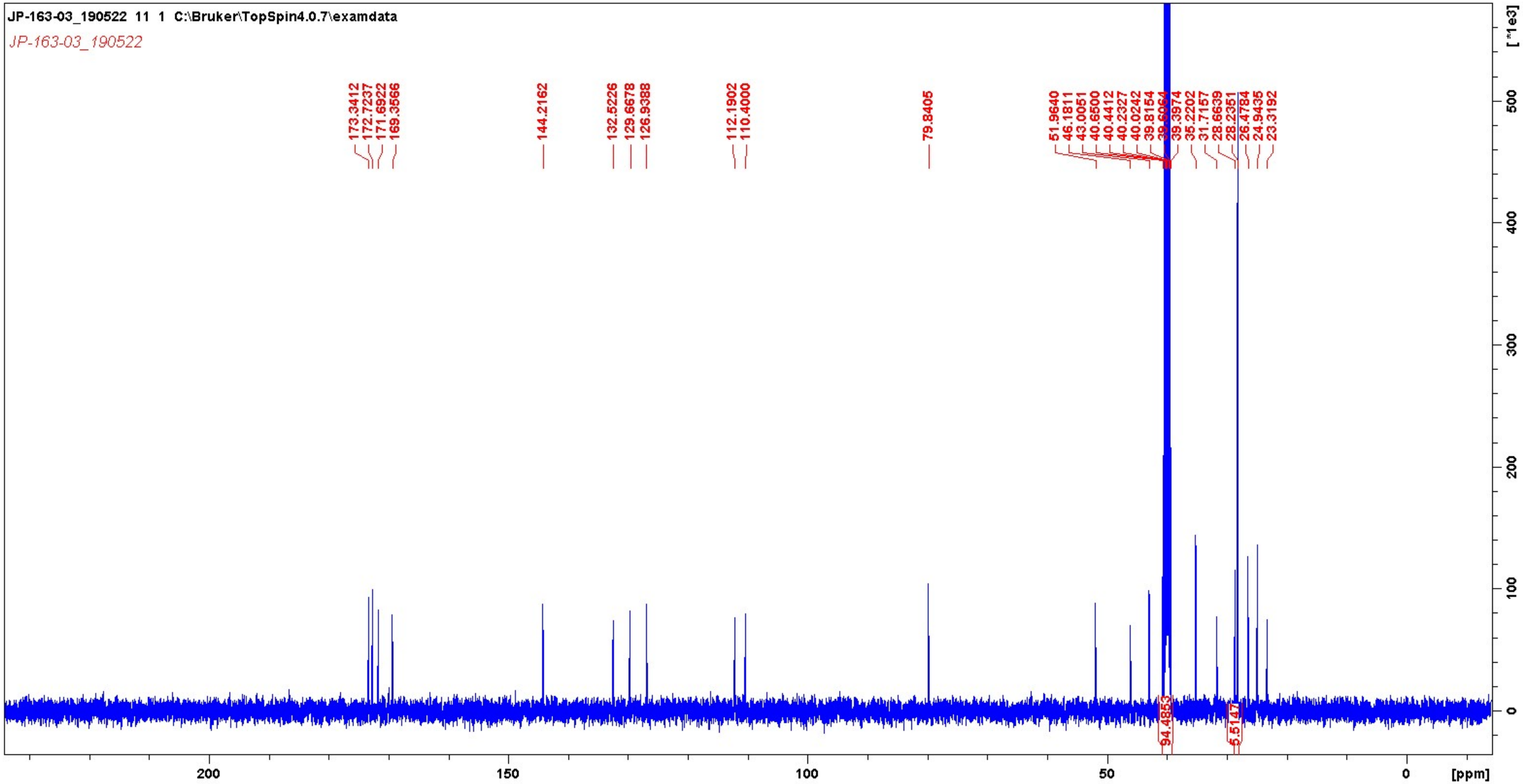

Figure S1-2. Carbon ^{13}C NMR of **1** (**JP-163-03**)

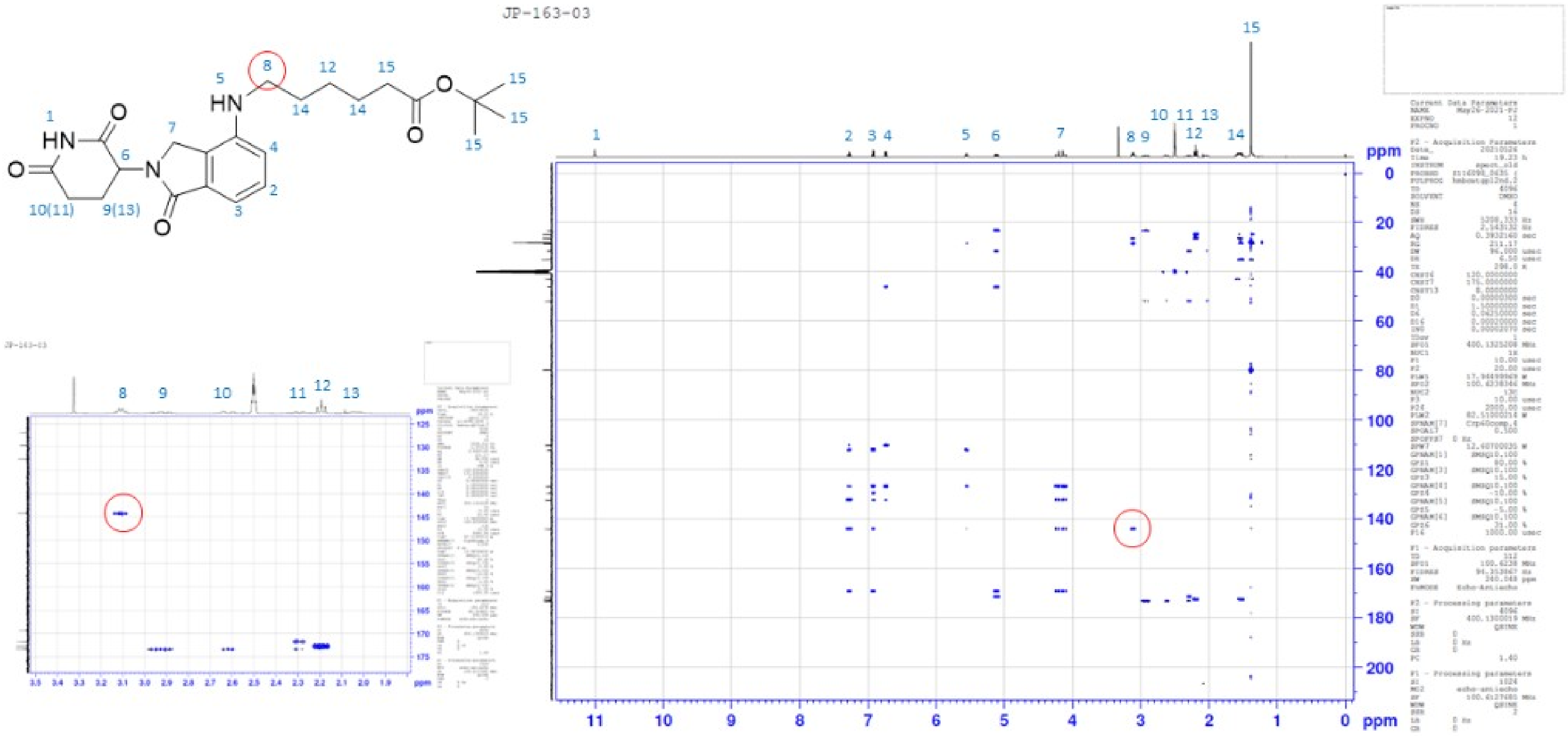

Figure S1-3. HMBC spectrum of **1** (**JP-163-03**)

6-((2-(2,6-dioxopiperidin-3-yl)-1-oxoisoindolin-4-yl) amino) hexanoic acid (**2**, **JP-164-05**)

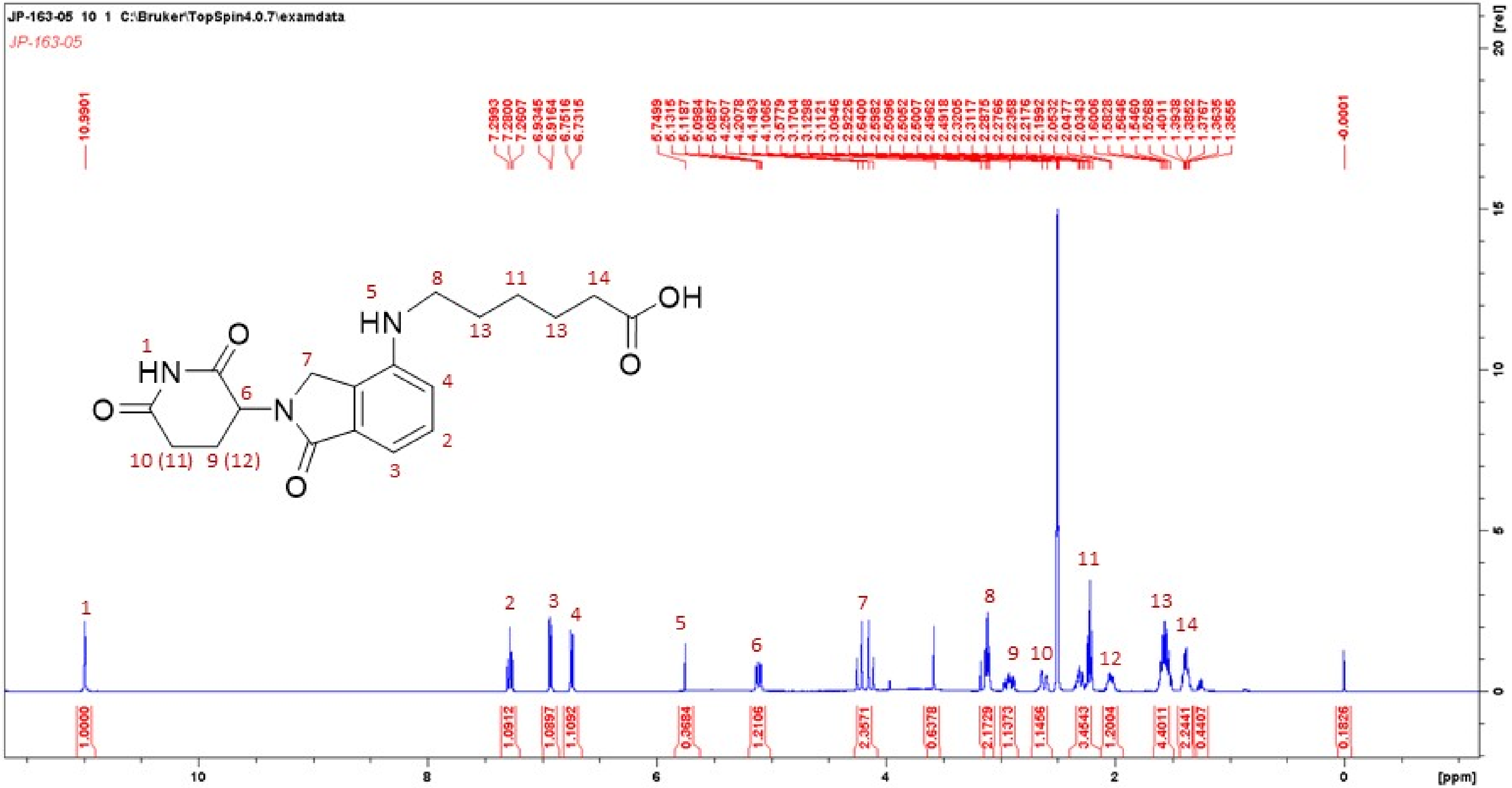

Figure S2-1. Proton ^{1}H NMR of **2** (**JP-163-05**)

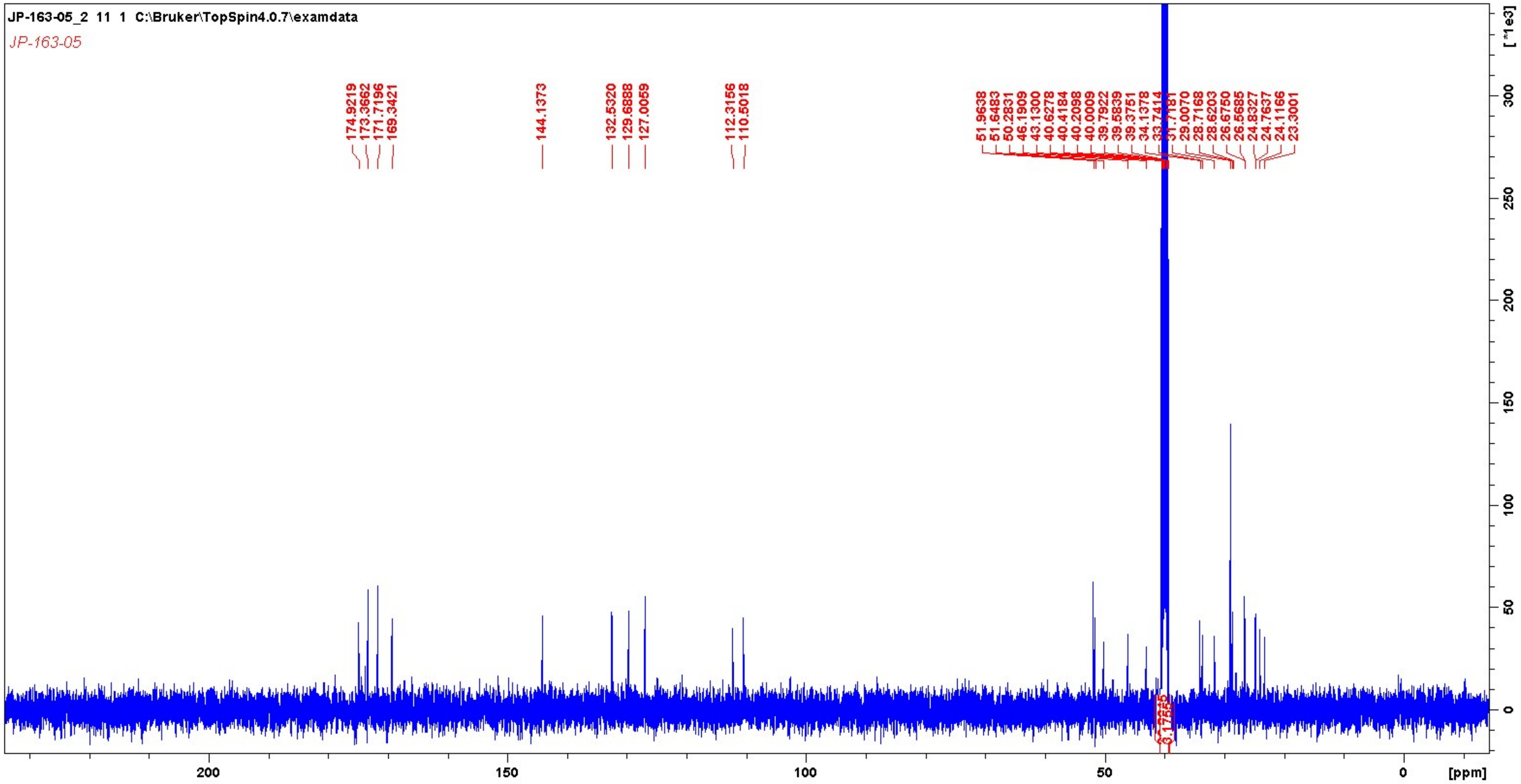

Figure S2-2. Carbon ^{13}C NMR of **2** (**JP-163-05**)

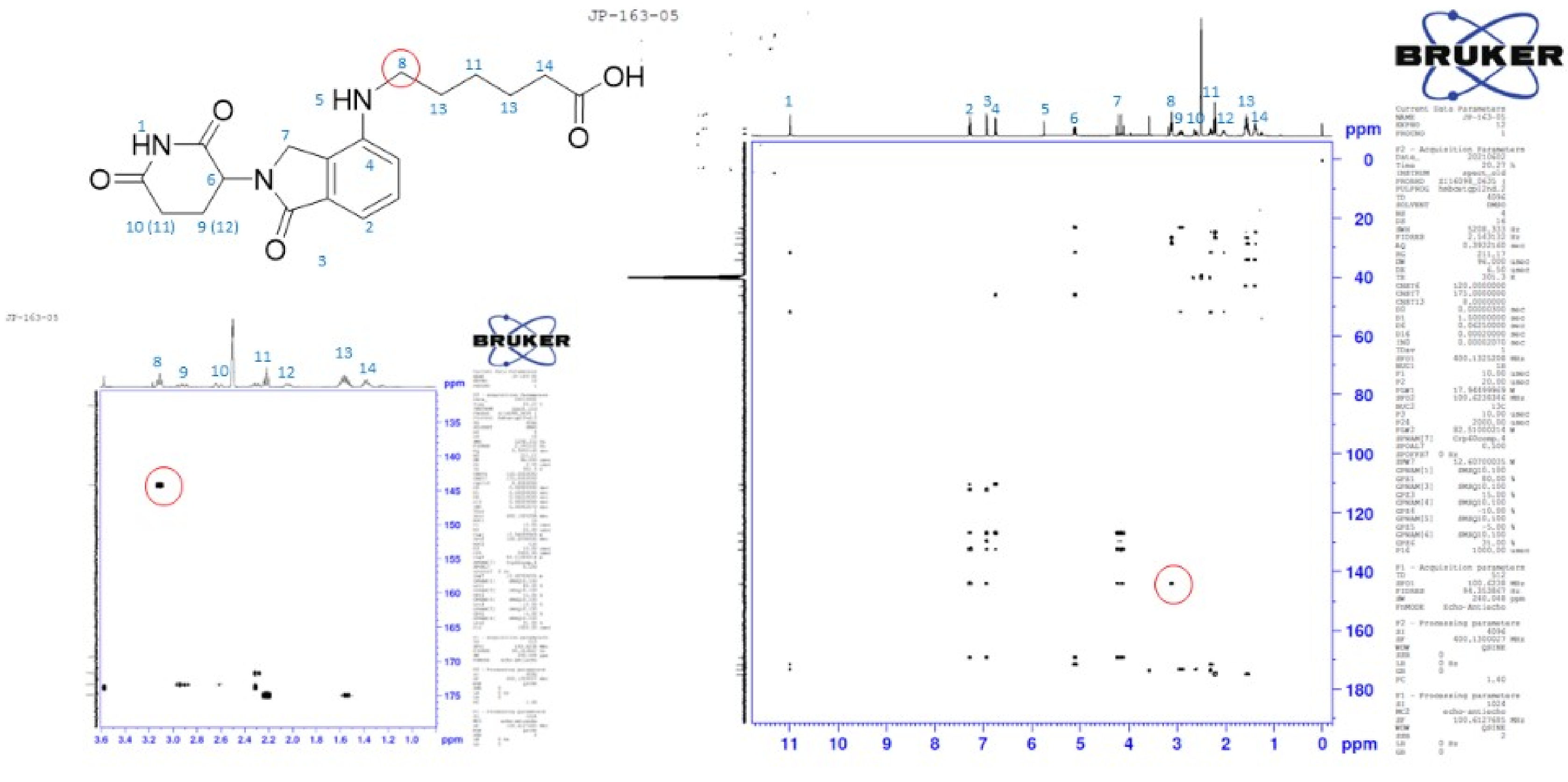

Figure S2-3. HMBC spectrum of **2** (**JP-163-05**)

6-((2-(2,6-dioxopiperidin-3-yl)-1-oxoisoindolin-4-yl) amino)-N-(3-fluoro-4-(4-(((S)-7-methoxy-5-oxo-2,3,5,11a-tetrahydro-1H-benzo[e]pyrrolo[1,2-a][1,4]diazepin-8-yl)oxy)butanamido)phenyl)hexanamide (**15a, JP-175-P6**)

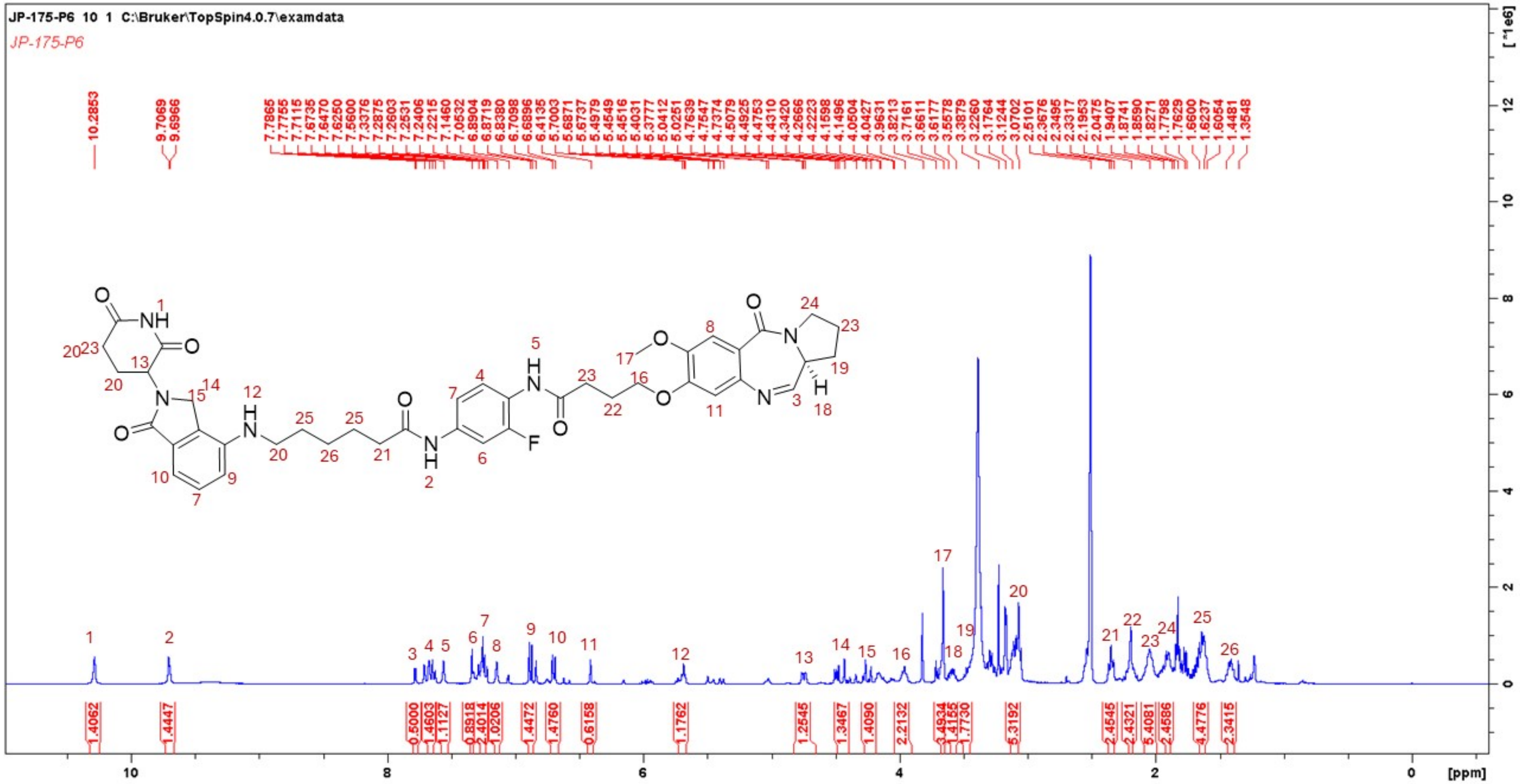

Figure S3-1. Proton ^{1}H NMR of **15a** (**JP-175-P6**)

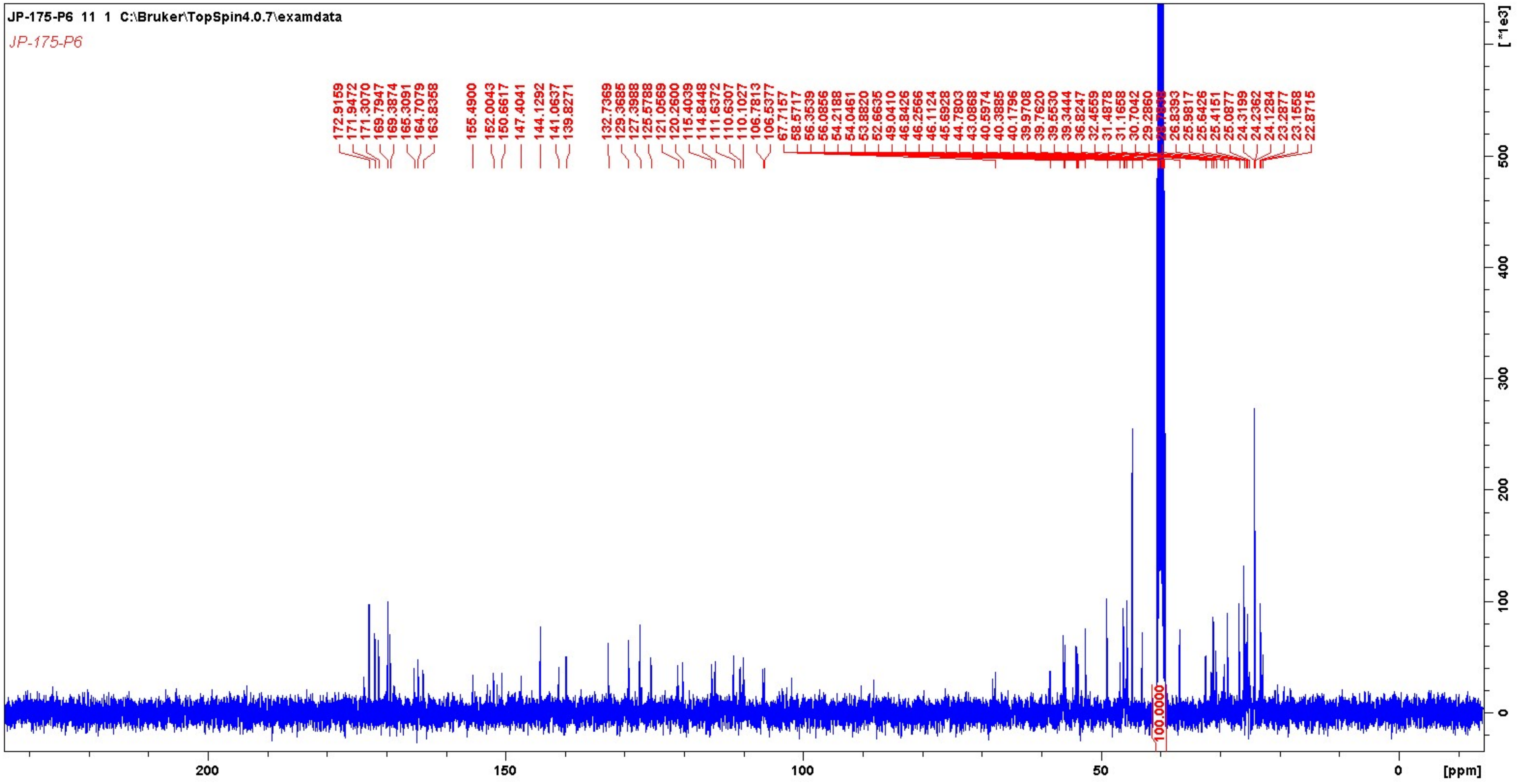

Figure S3-2. Carbon ^{13}C NMR of **15a** (**JP-175-P6**)

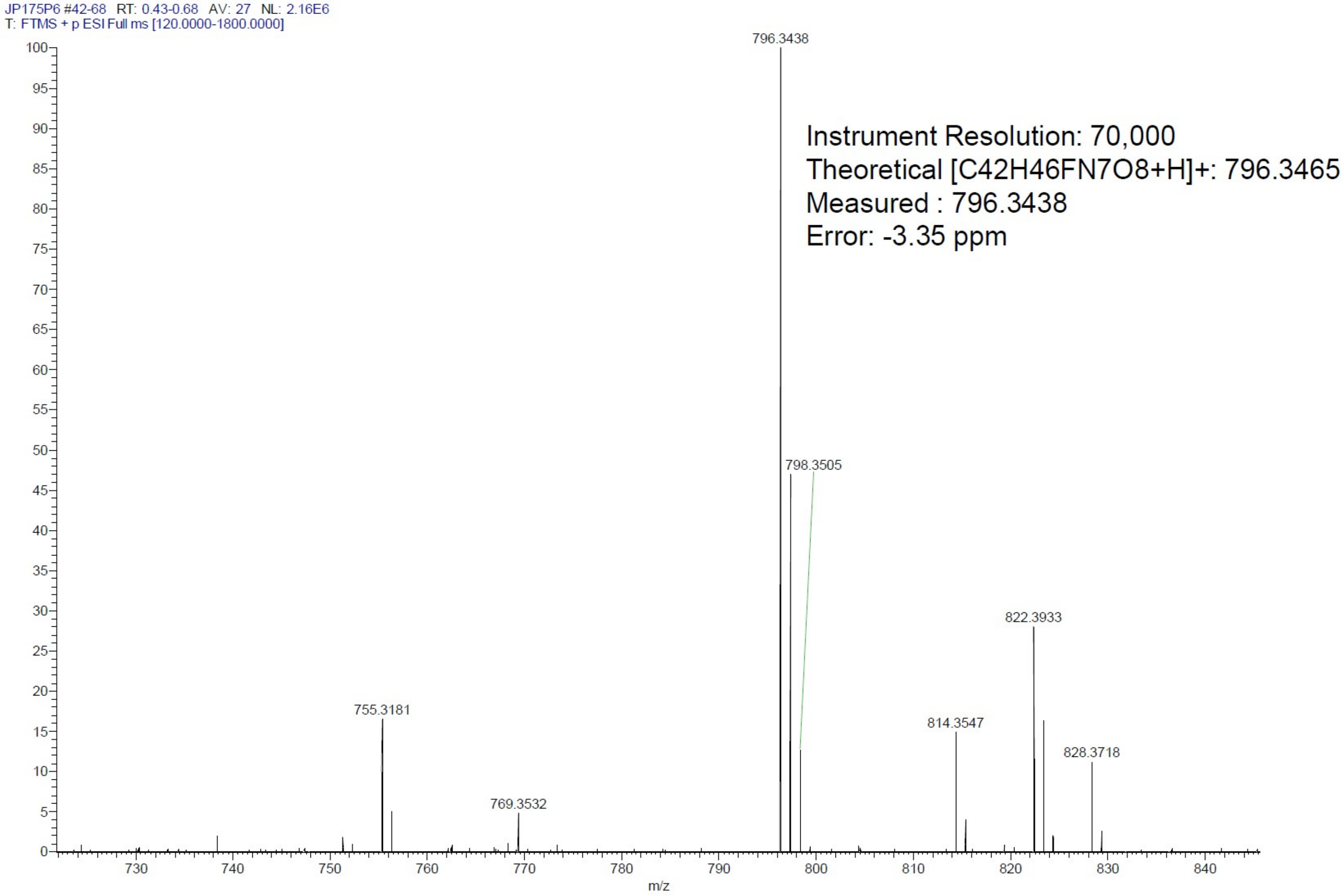

Figure S3-3. HRMS result of **15a** (**JP-175-P6**)

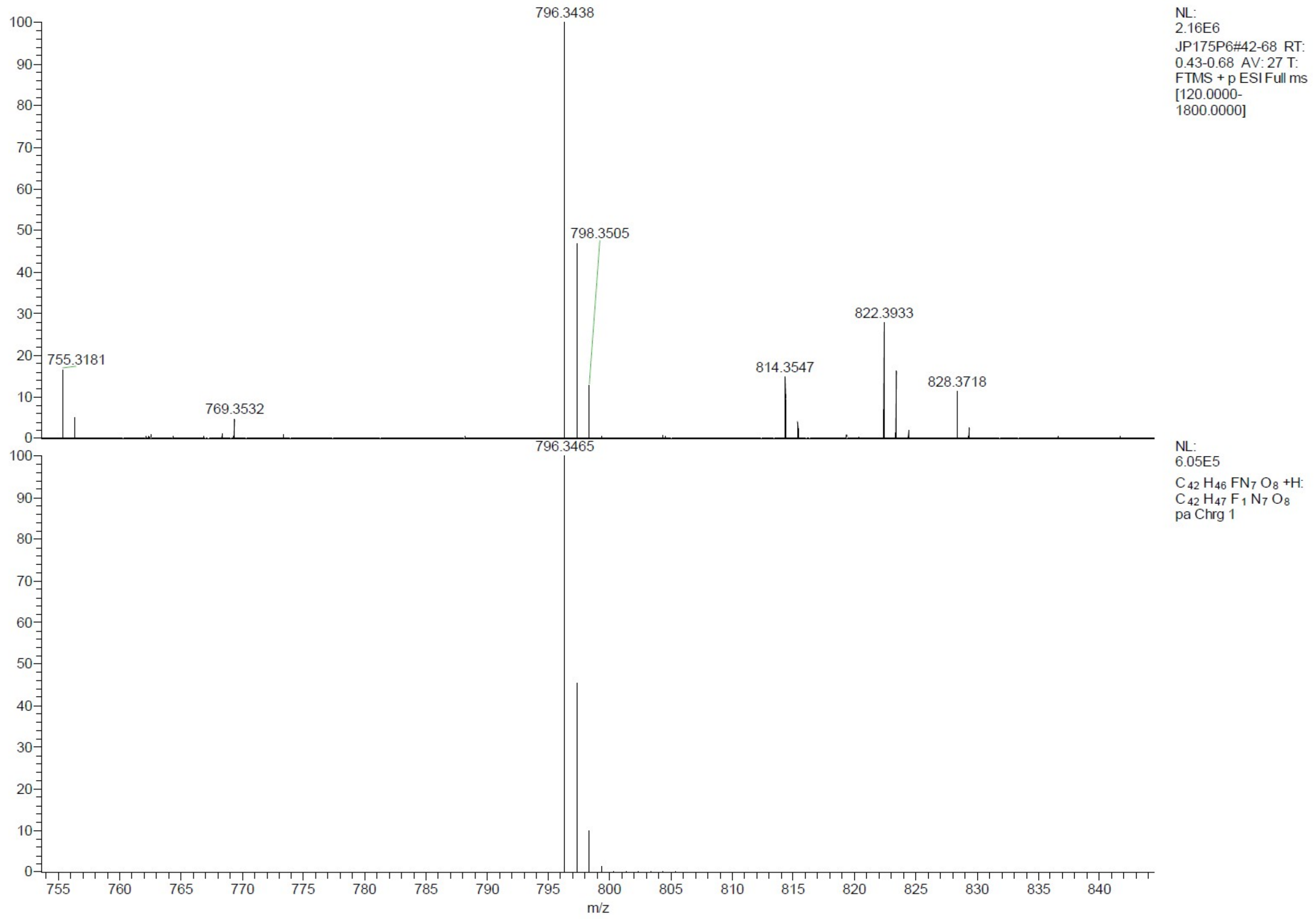

Figure S3-4. HRMS result of **15a** (**JP-175-P6**)

6-((2-(2,6-dioxopiperidin-3-yl)-1-oxoisoindolin-4-yl)amino)-N-(4-(4-(((S)-7-methoxy-5-oxo-2,3,5,11a-tetrahydro-1H-benzo[e]pyrrolo[1,2-a][1,4]diazepin-8-yl)oxy)butanamido)phenyl)hexanamide (**15d, JP-163-16**)

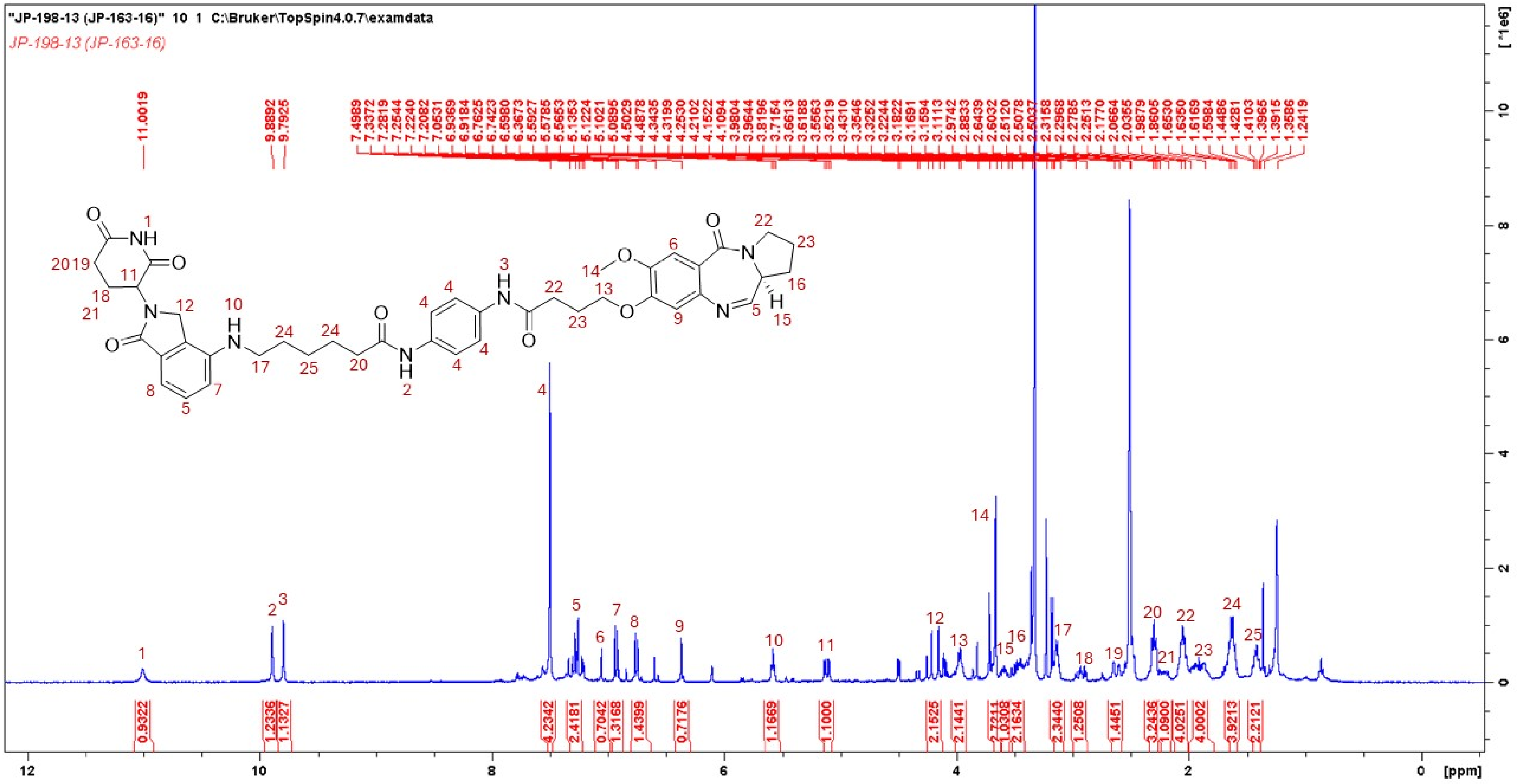

Figure S4-1. Proton NMR of **15d** (**JP-163-16/JP-198-13**)

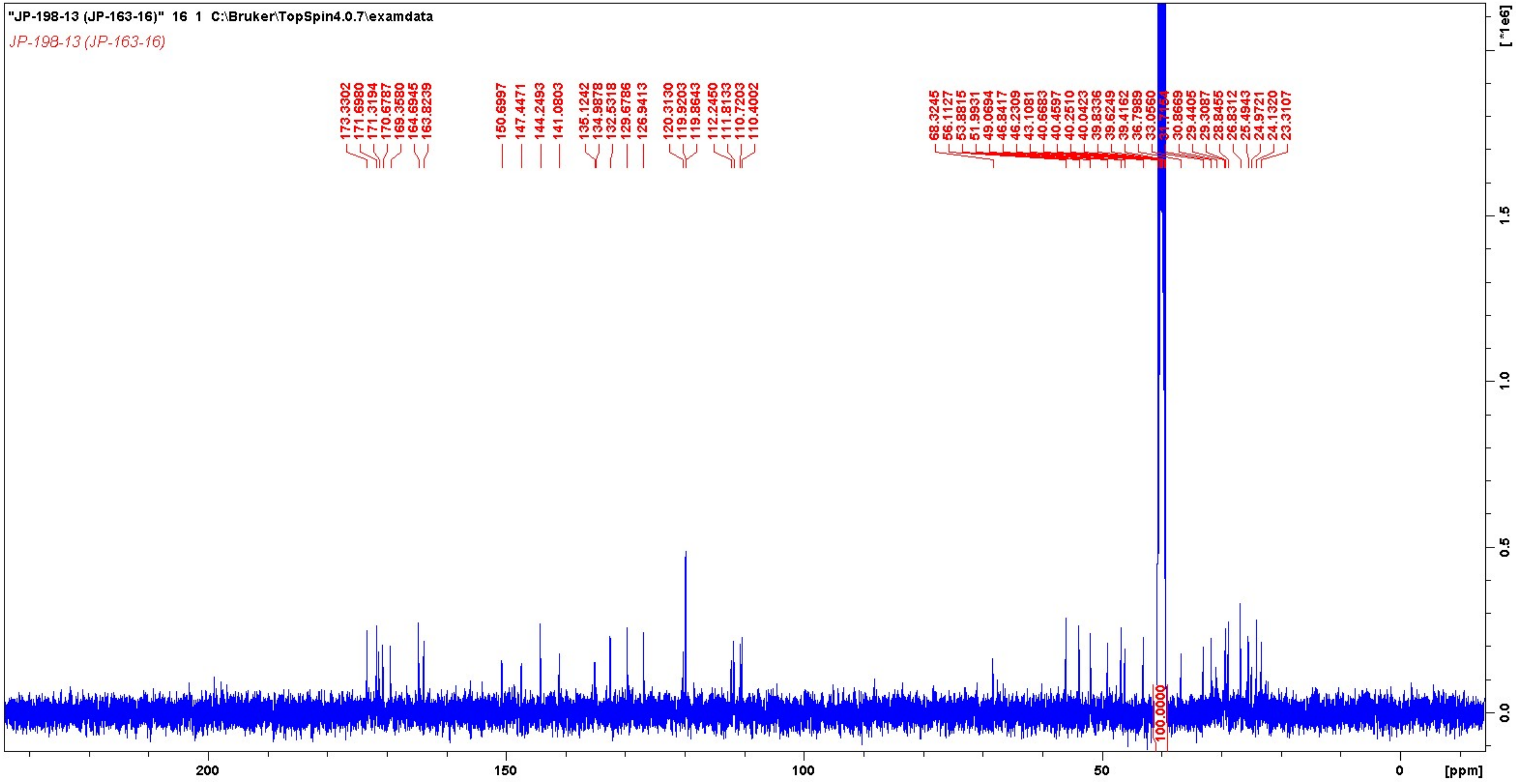

Figure S4-2. Carbon NMR of **15d** (**JP-163-16/JP-198-13**)

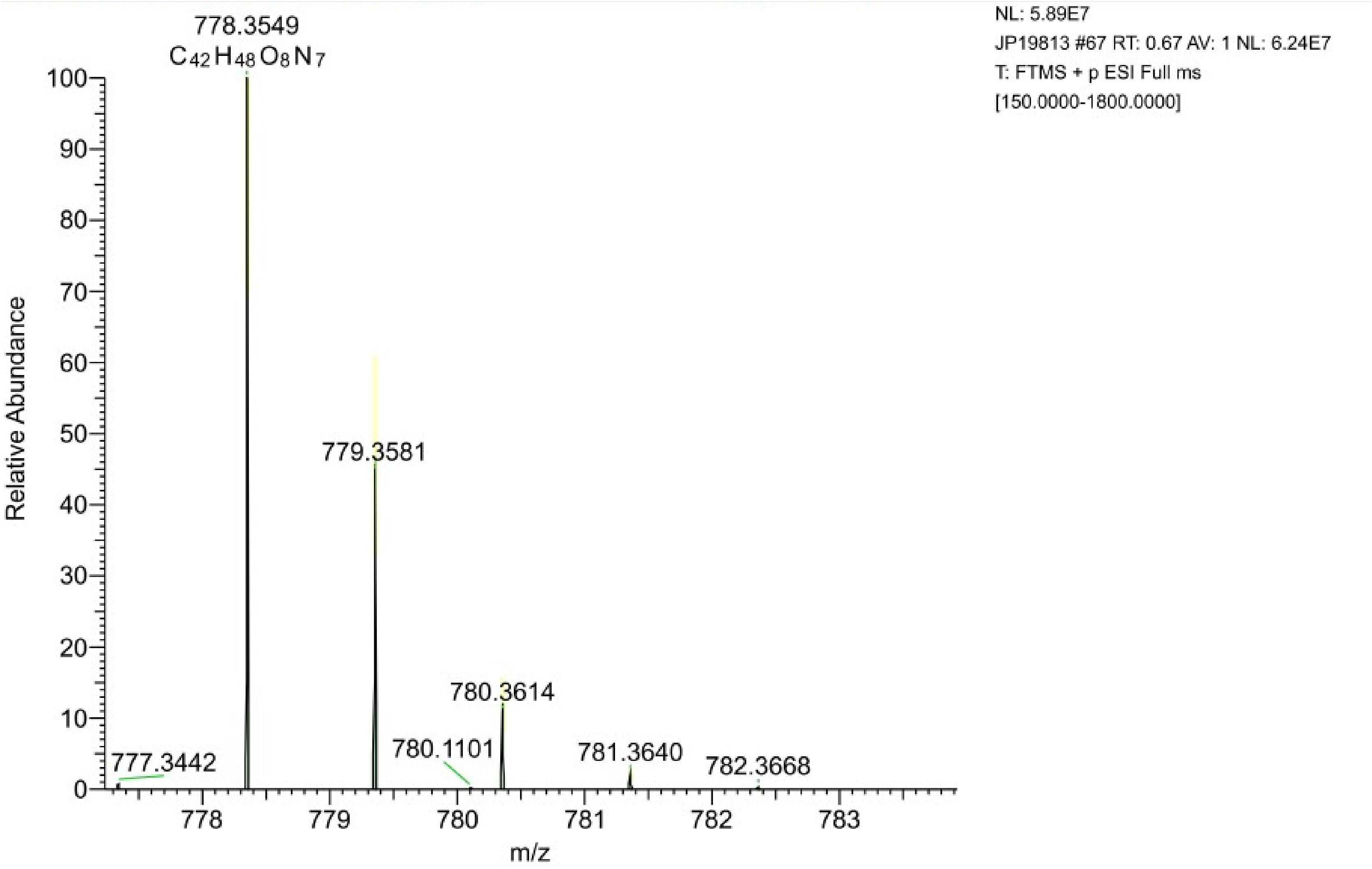

Figure S4-3. HRMS result of **15d** (**JP-163-16/JP-198-13**)

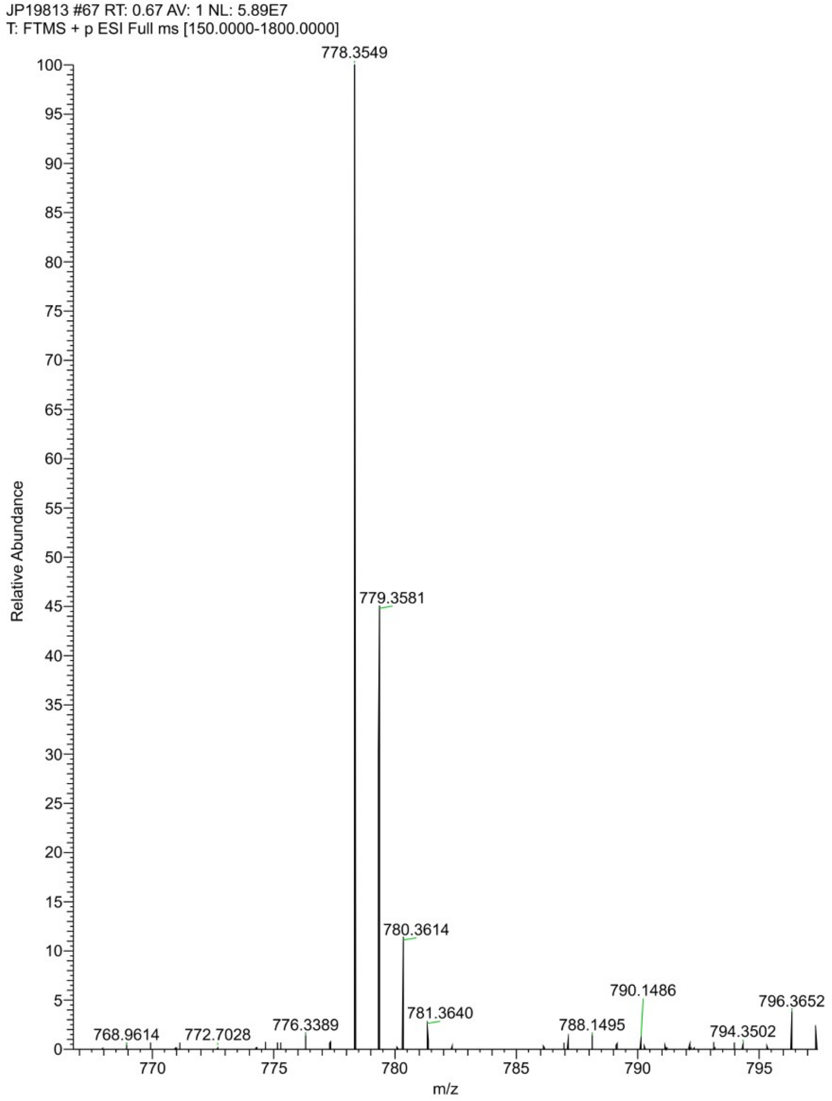

Figure S4-4. HRMS result of **15d** (**JP-163-16/JP-198-13**)

6-((2-(2,6-dioxopiperidin-3-yl)-1-oxoisoindolin-4-yl)amino)-N-(4-(4-(((S)-7-methoxy-5-oxo-2,3,5,11a-tetrahydro-1H-benzo[e]pyrrolo[1,2-a][1,4]diazepin-8-yl)oxy)butanamido)-3-methylphenyl)hexanamide (**15b, JP-179-P6**)

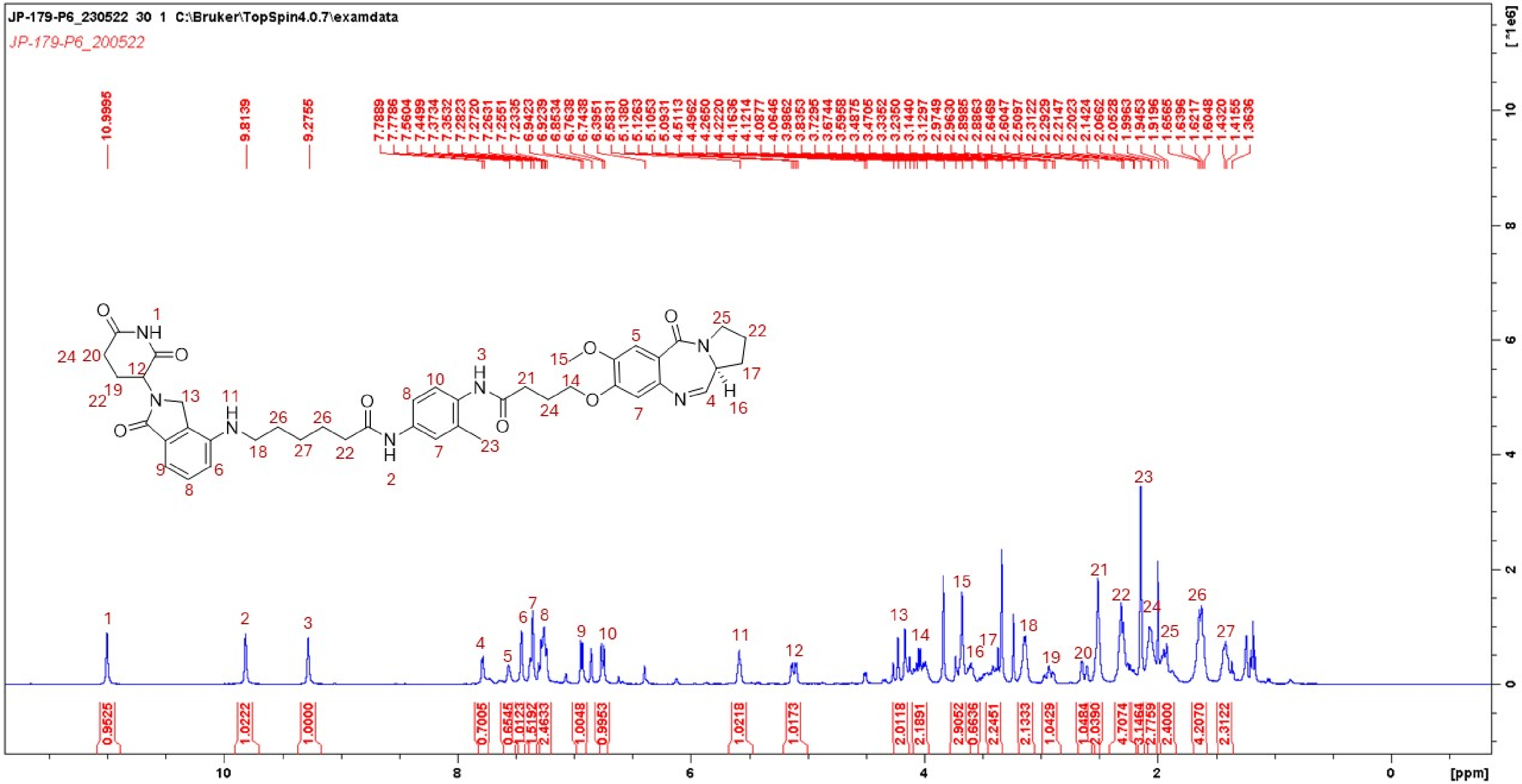

Figure S5-1. Proton NMR of **15b** (**JP-179-P6**)

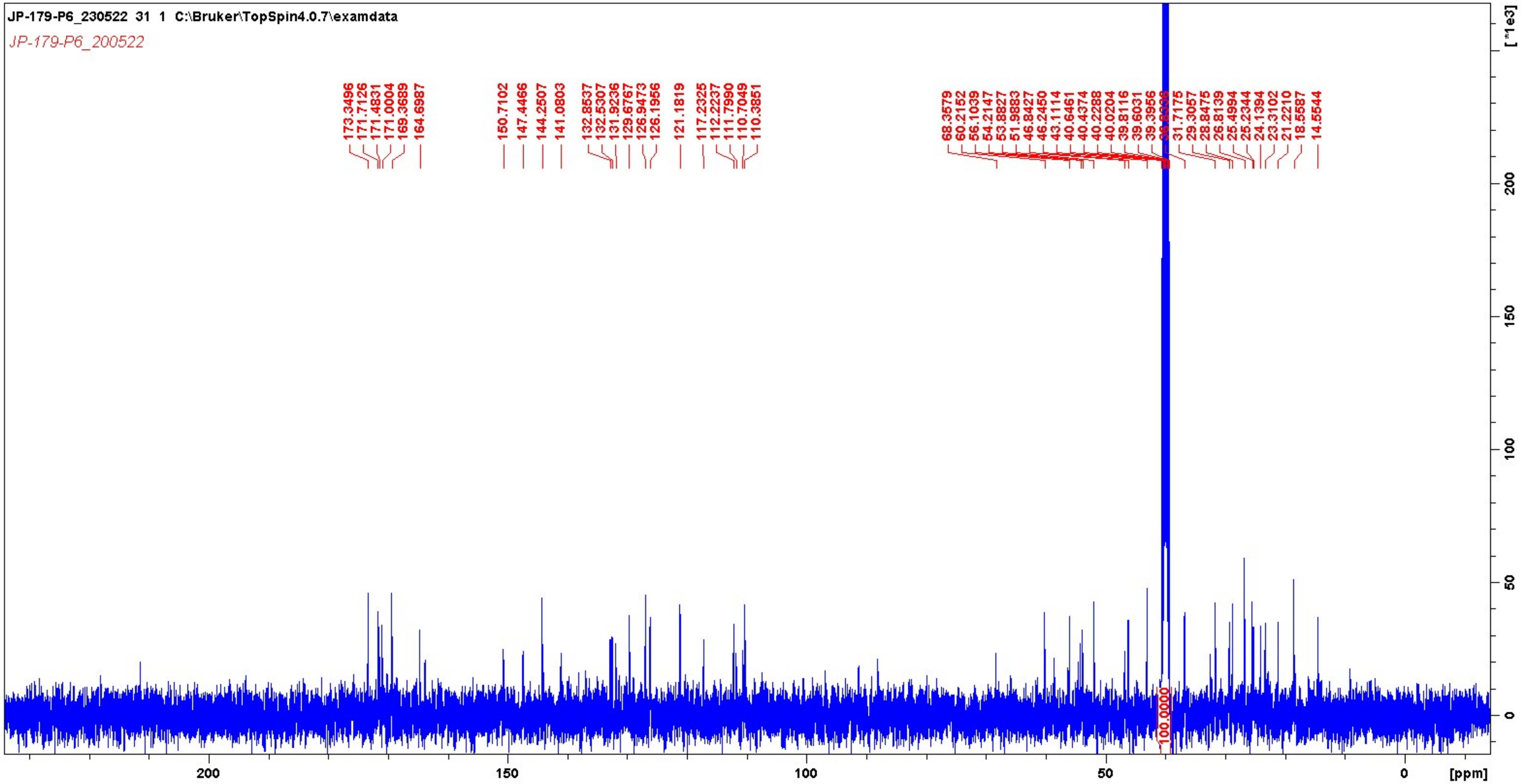

Figure S5-2. Carbon NMR of **15b** (**JP-179-P6**)

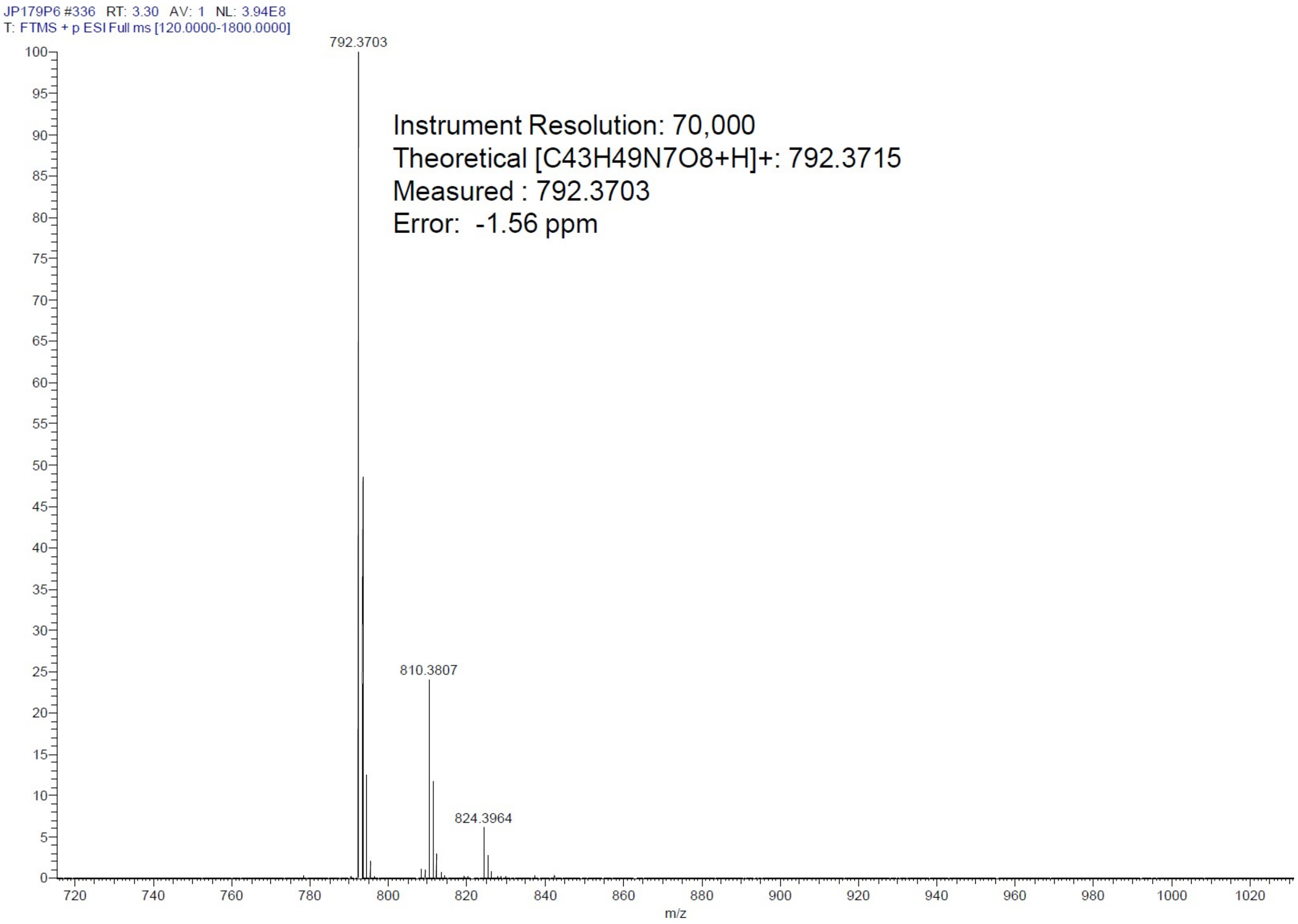

Figure S5-3. HRMS result of **15b** (**JP-179-P6**)

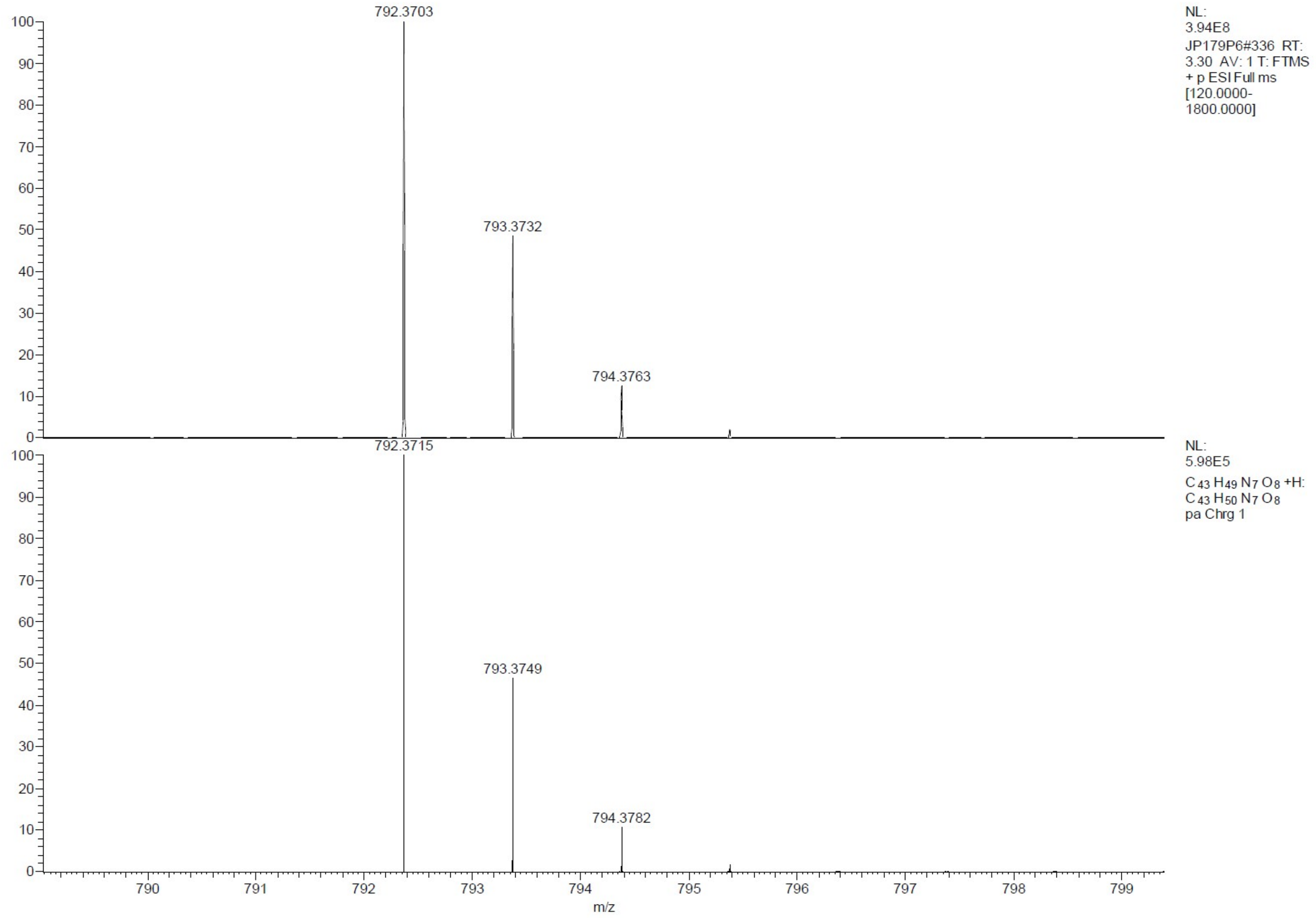

Figure S5-4. HRMS result of **15b** (**JP-179-P6**)

6-((2-(2,6-dioxopiperidin-3-yl)-1-oxoisoindolin-4-yl)amino)-N-(3-methoxy-4-(4-(((S)-7-methoxy-5-oxo-2,3,5,11a-tetrahydro-1H-benzo[e]pyrrolo[1,2-a][1,4]diazepin-8-yl)oxy)butanamido)phenyl)hexanamide **(15c, JP-179-P12)**

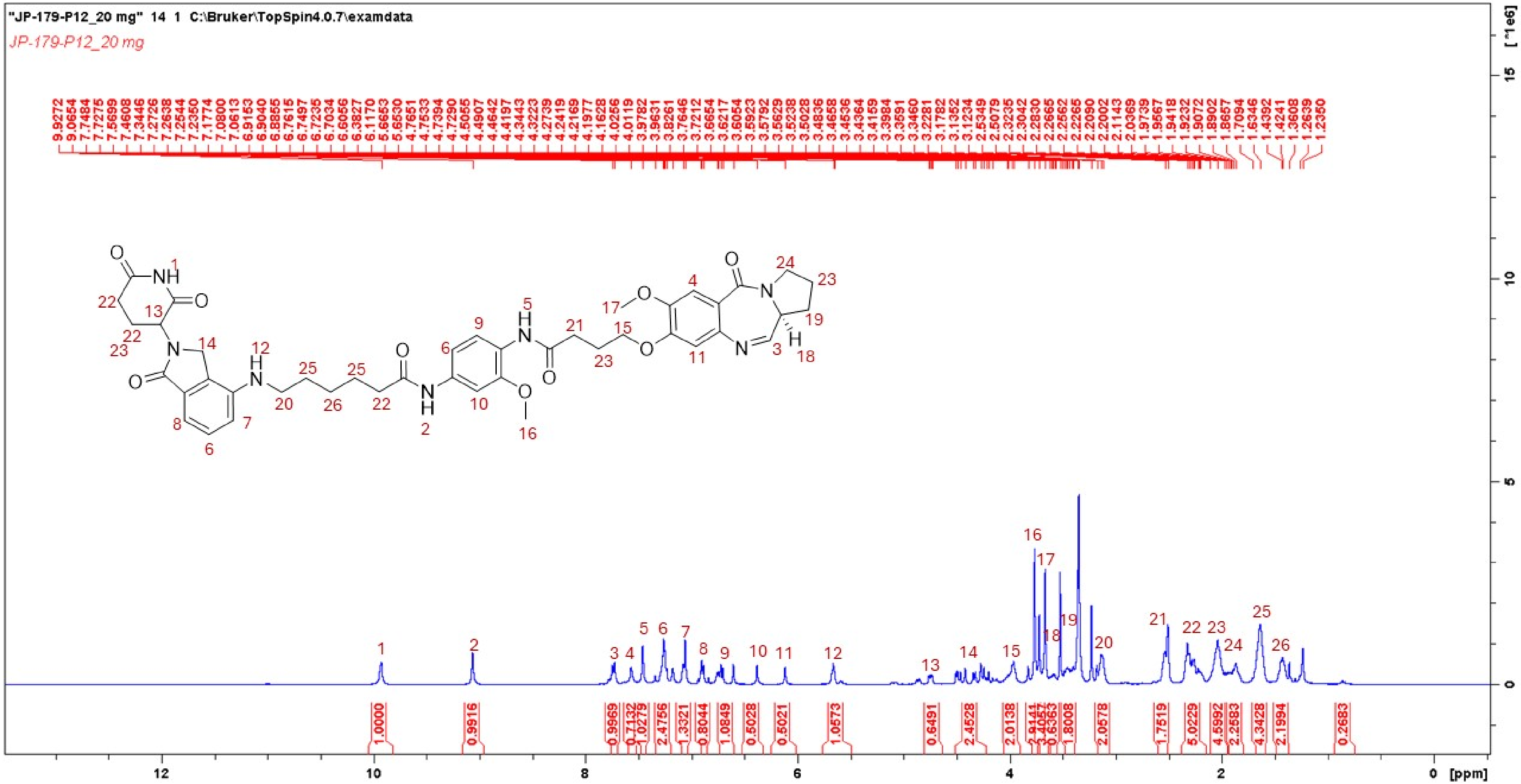

Figure S6-1. Proton NMR of **15c** (**JP-179-P12**)

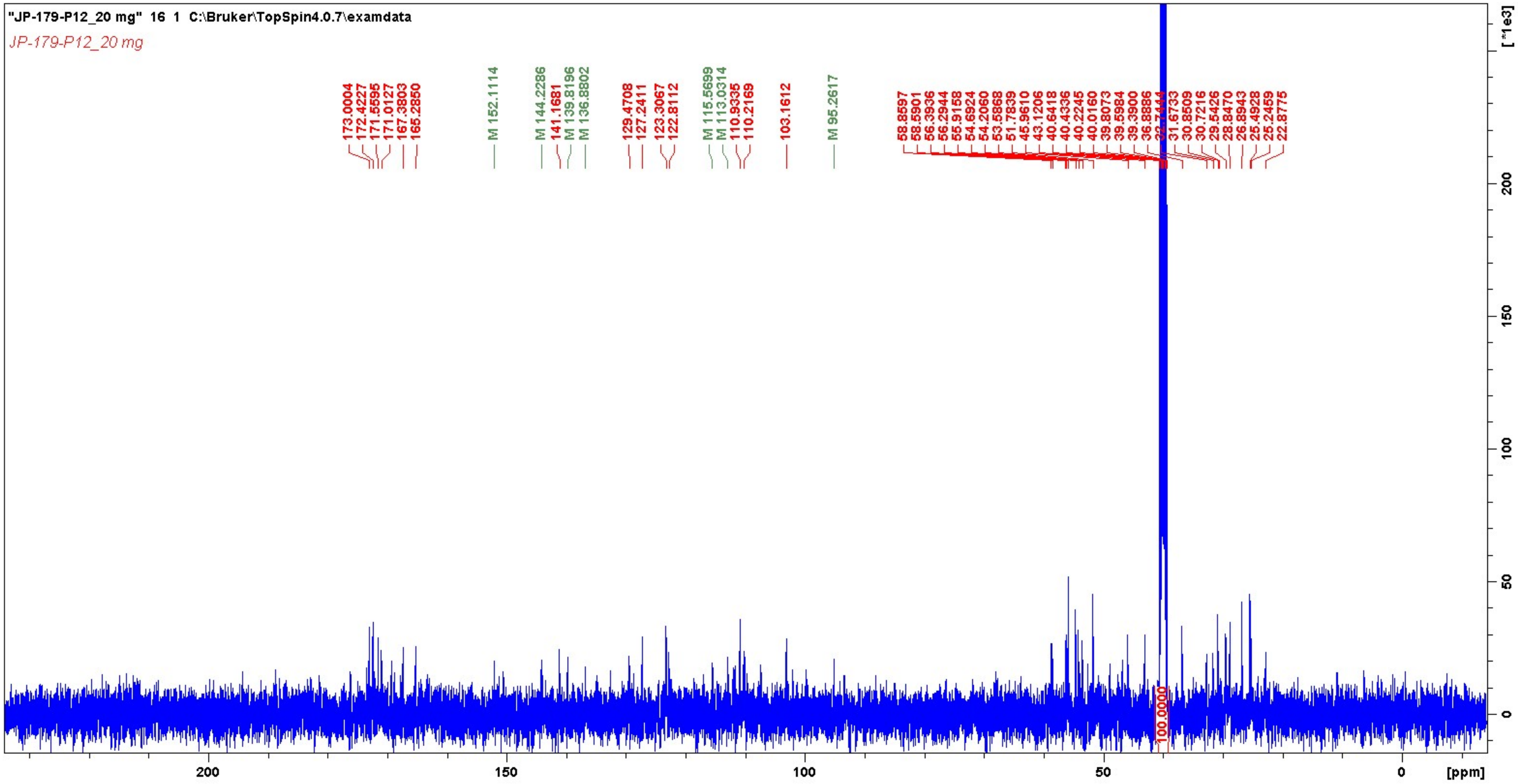

Figure S6-2. Carbon NMR of **15c** (**JP-179-P12**)

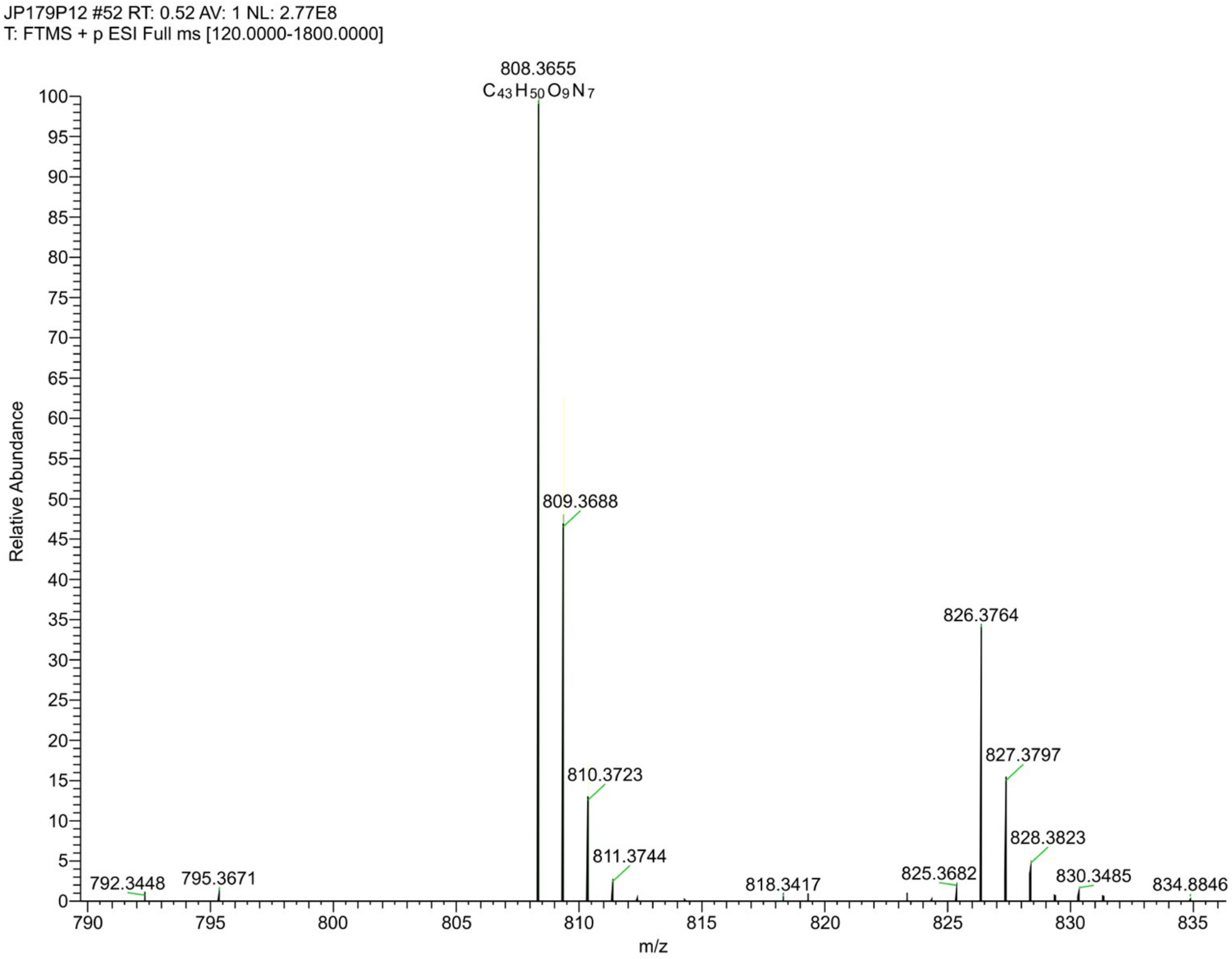

Figure S6-3. HRMS result of **15c** (**JP-179-P12**)

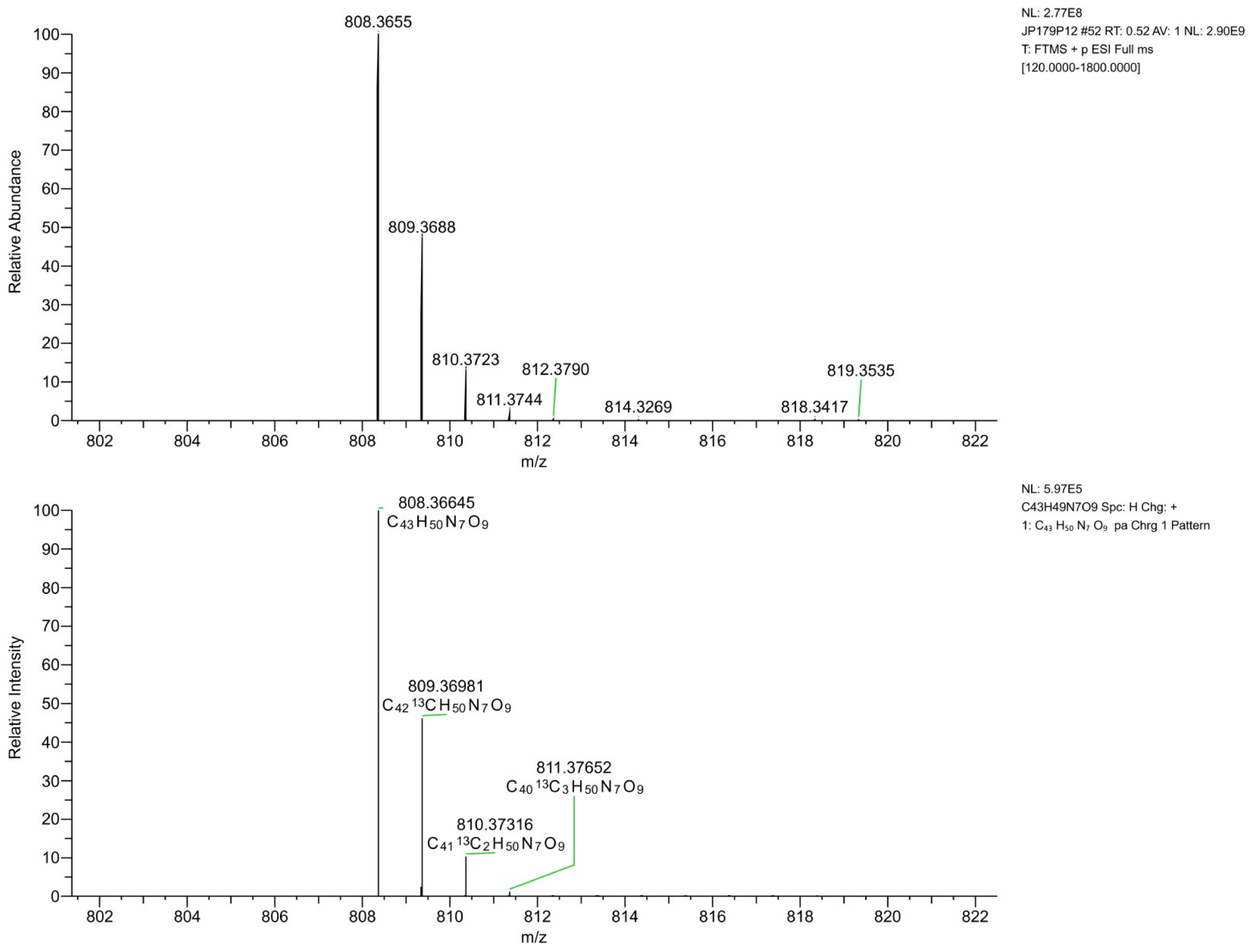

Figure S6-4. HRMS result of **15c** (**JP-179-P12**)

(S)-N-(4-amino-2-fluorophenyl)-4-((7-methoxy-5-oxo-2,3,5,11a-tetrahydro-1H-benzo[e]pyrrolo[1,2-a][1,4]diazepin-8-yl)oxy)butanamide (**20a, JP-193-12**)

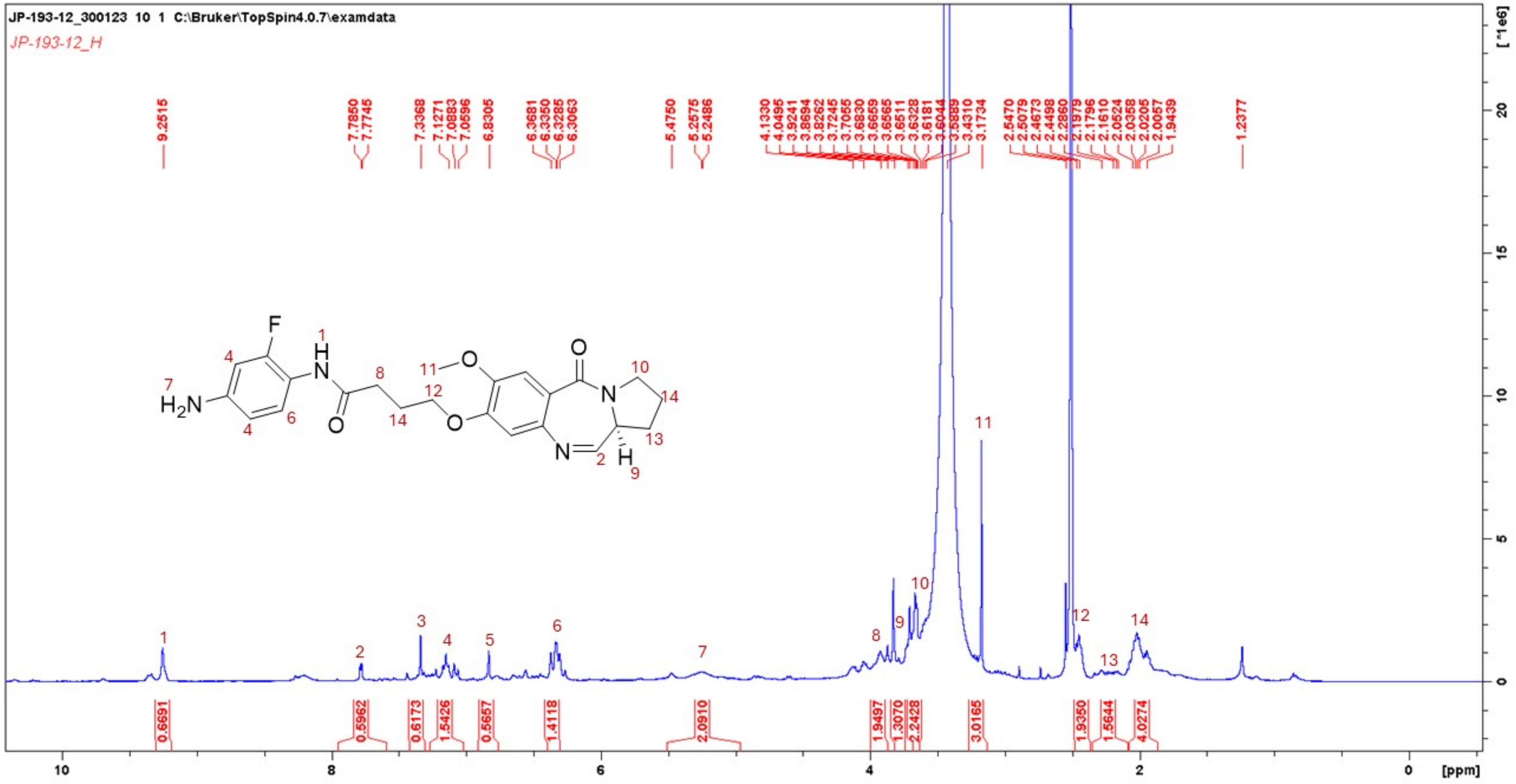

Figure S7-1. Proton NMR of **20a** (**JP-193-12**)

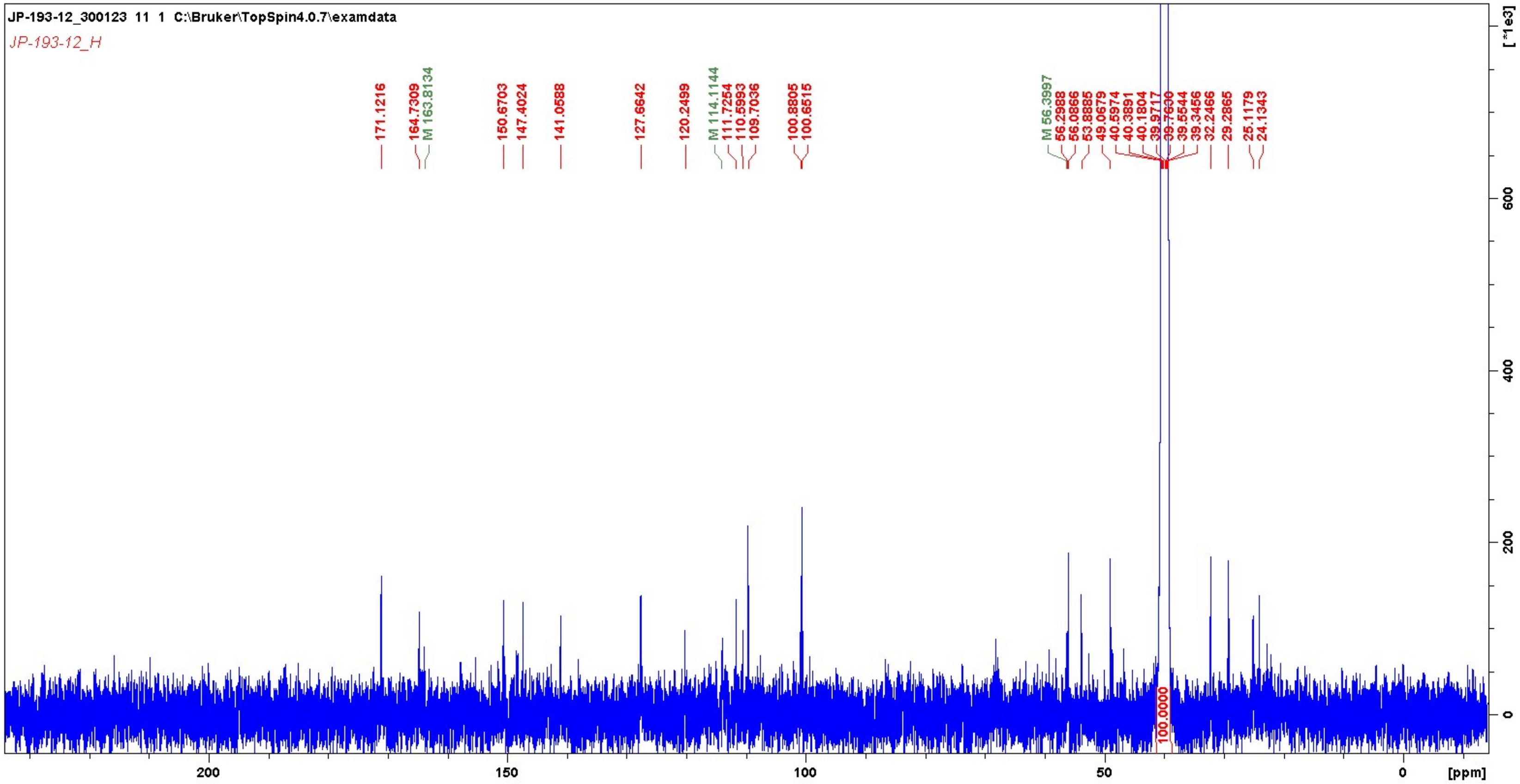

Figure S7-2. Carbon NMR of **20a** (**JP-193-12**)

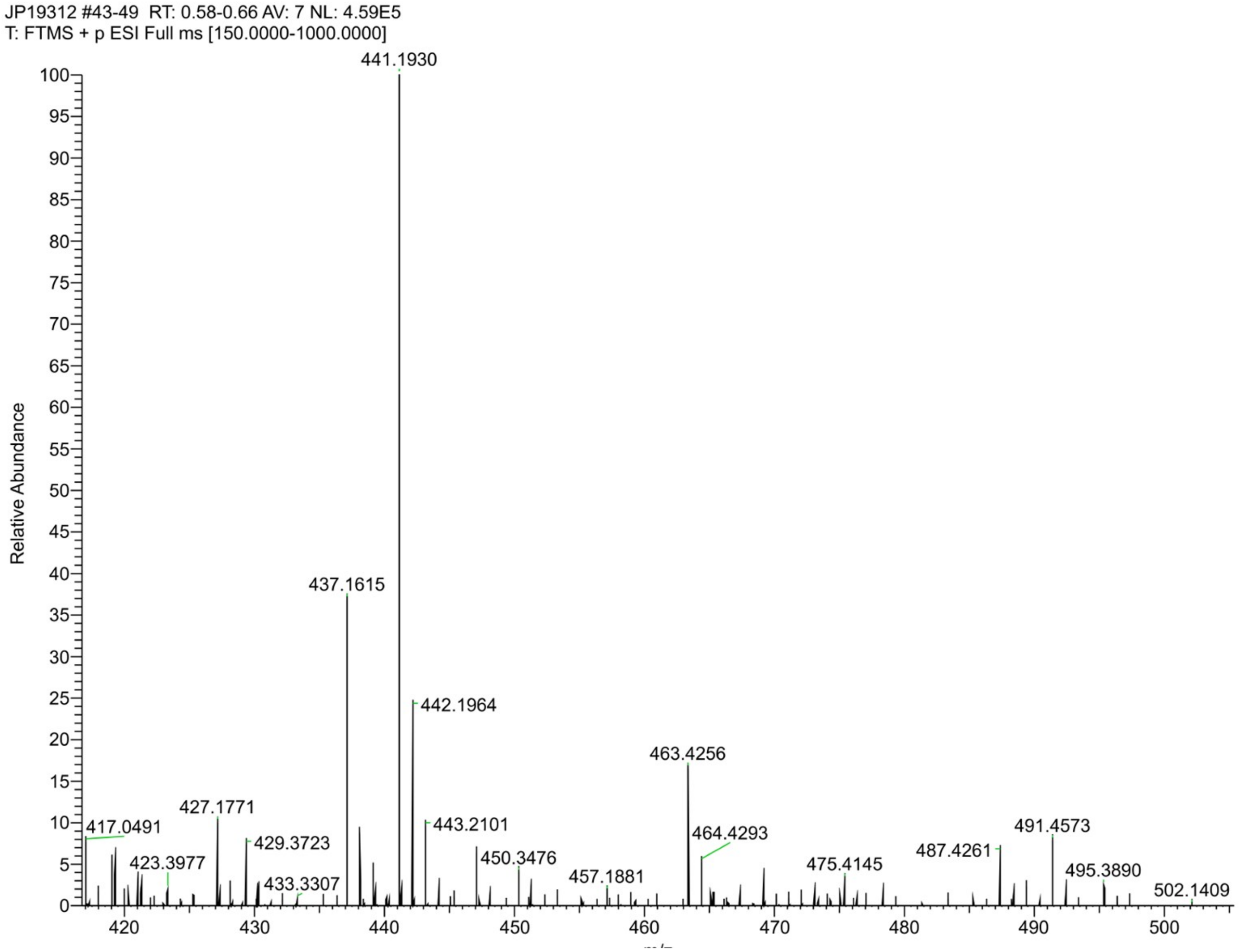

Figure S7-3. HRMS result of **20a** (**JP-193-12**)

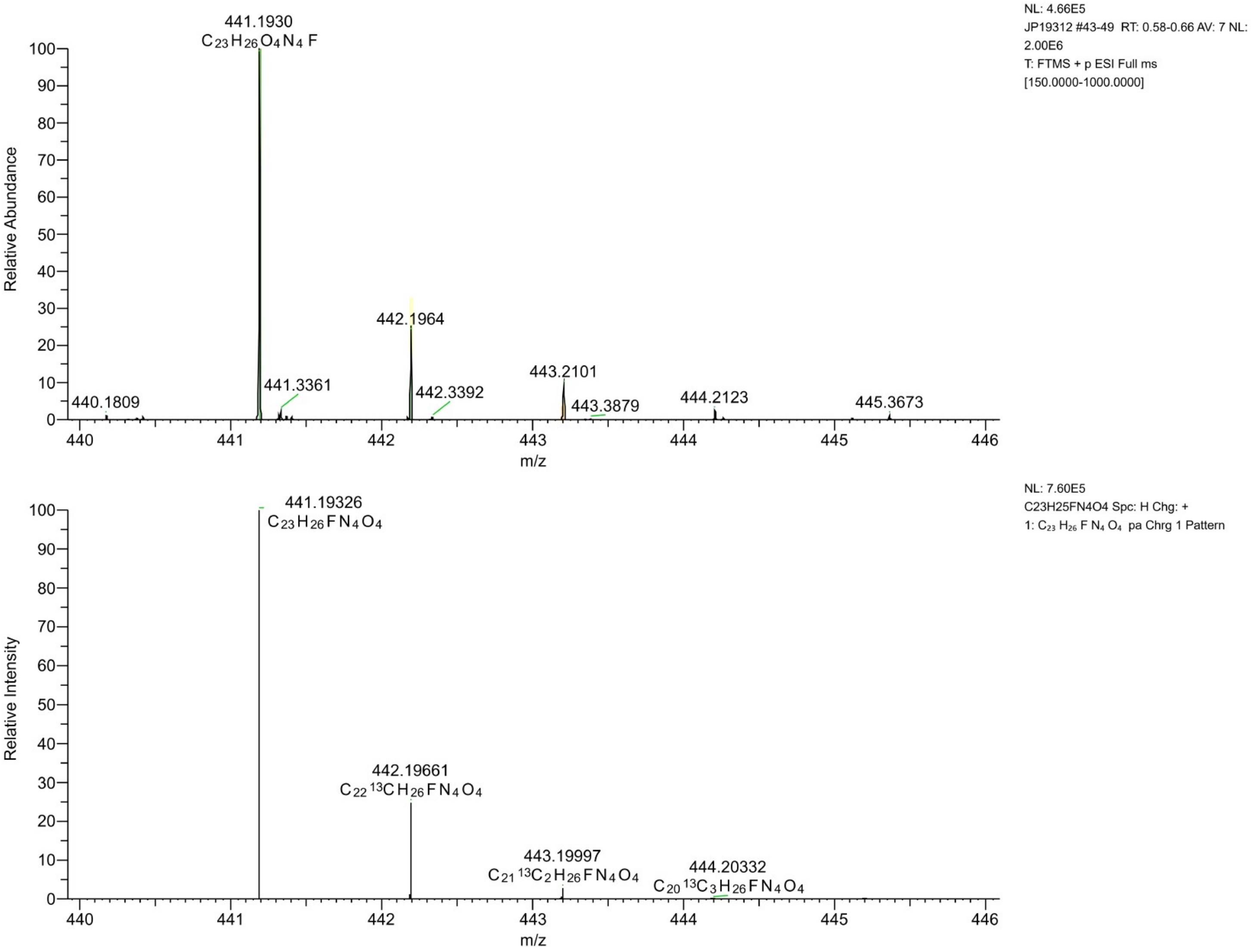

Figure S7-4. HRMS result of **20a** (**JP-193-12**)

(S)-N-(4-amino-2-methylphenyl)-4-((7-methoxy-5-oxo-2,3,5,11a-tetrahydro-1H-benzo[e]pyrrolo[1,2-a][1,4]diazepin-8-yl)oxy)butanamide (**20b, JP-193-16**)

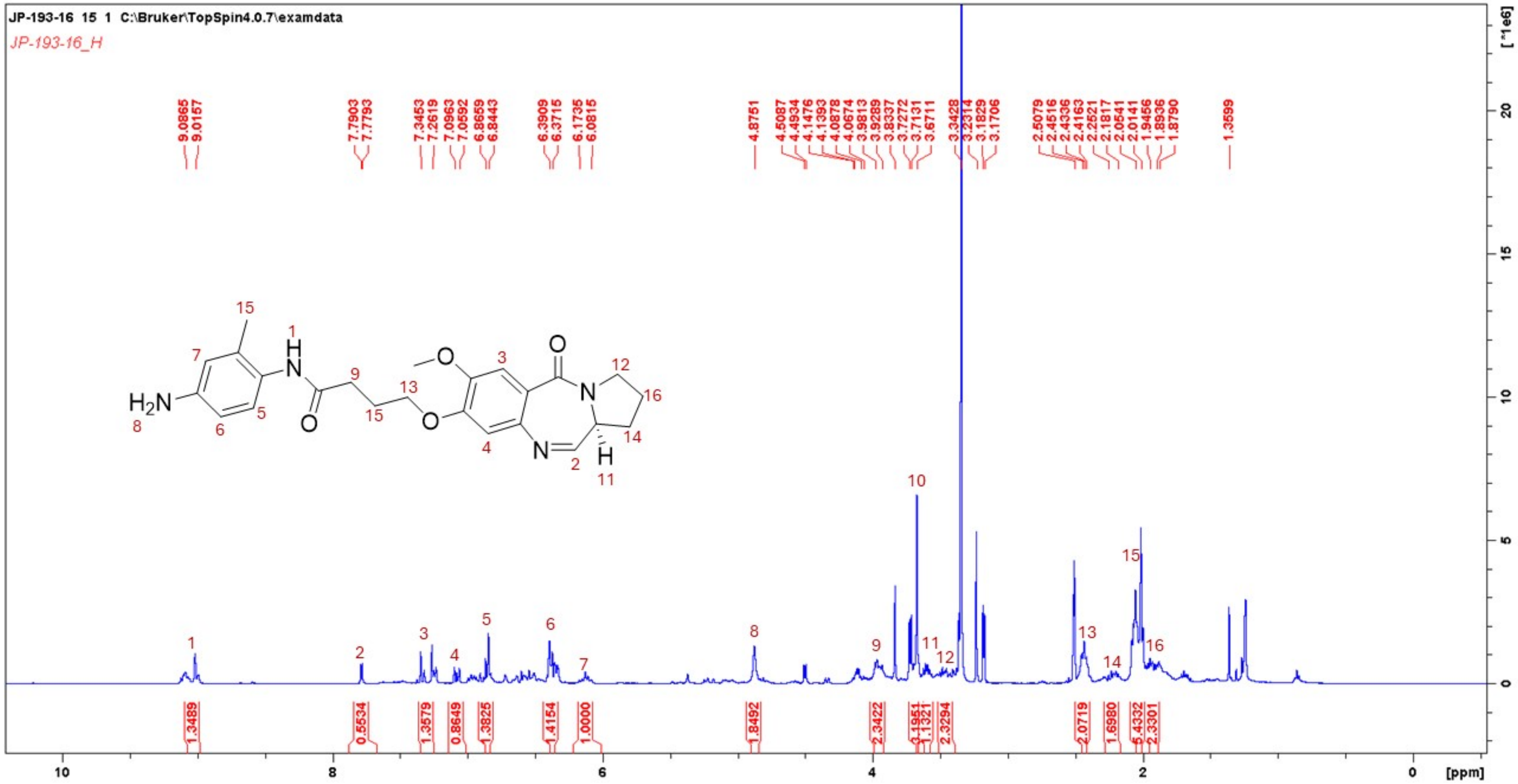

Figure S8-1. Proton NMR of **20b** (**JP-193-16**)

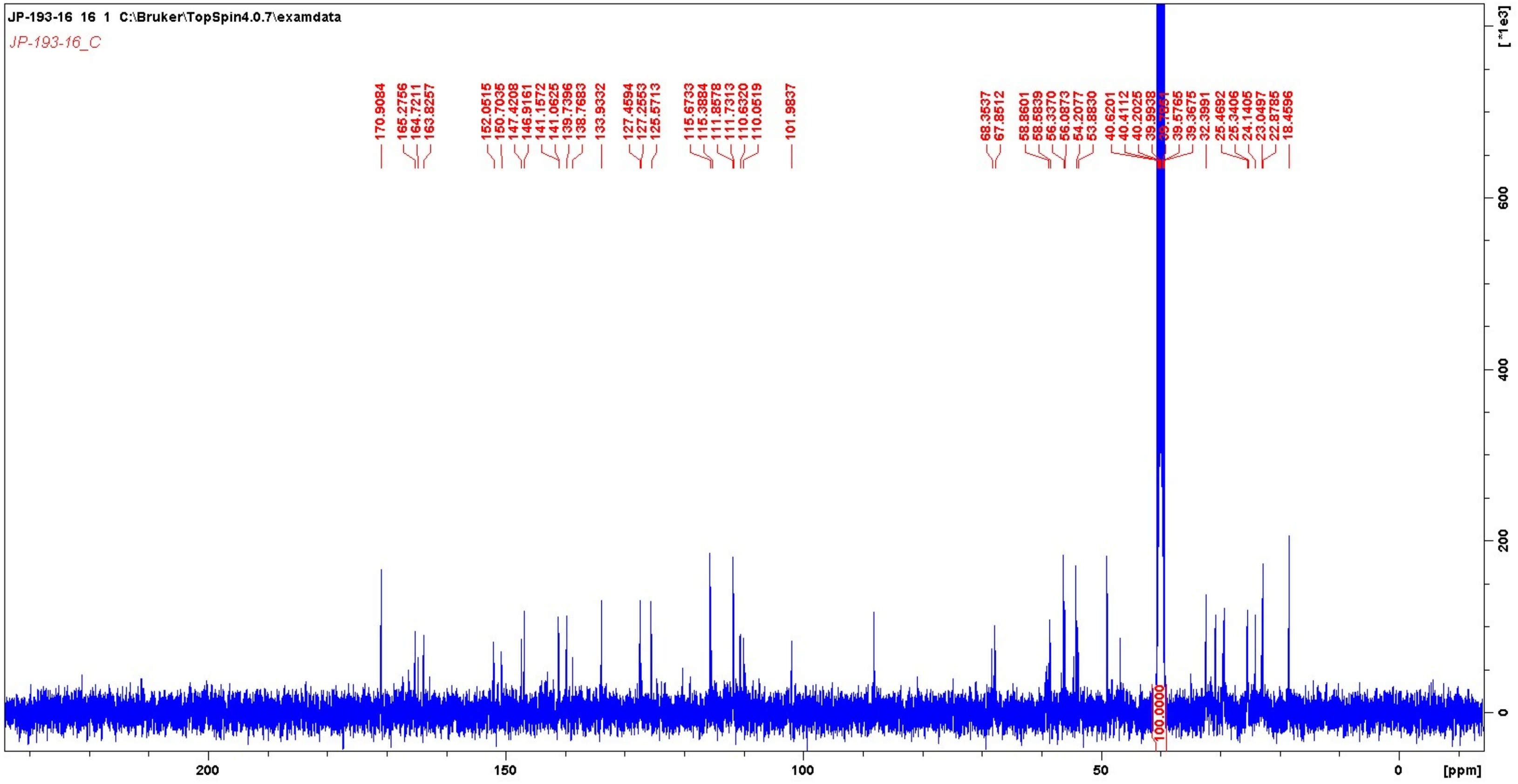

Figure S8-2. Carbon NMR of **20b** (**JP-193-16**)

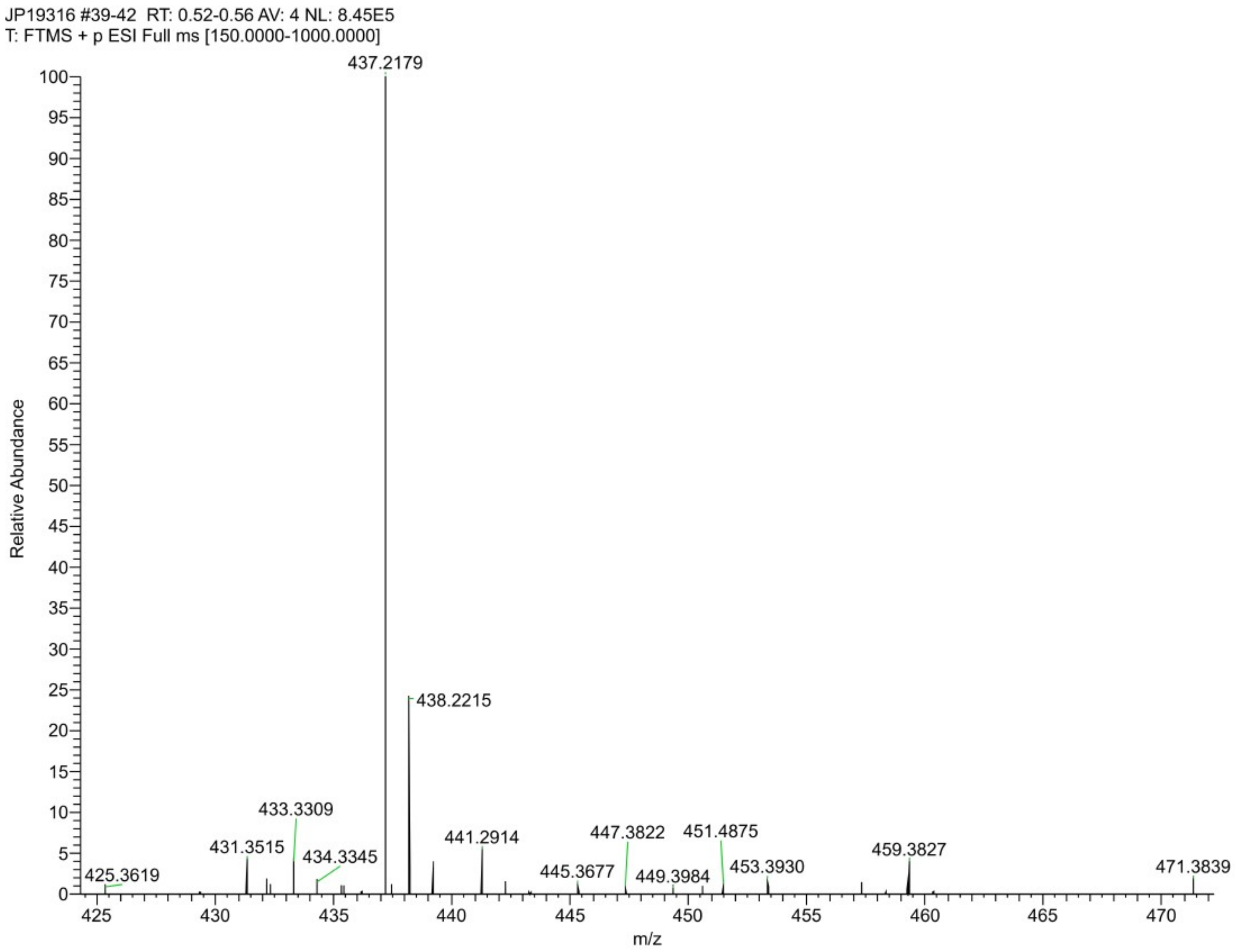

Figure S8-3. HRMS result of **20b** (**JP-193-16**)

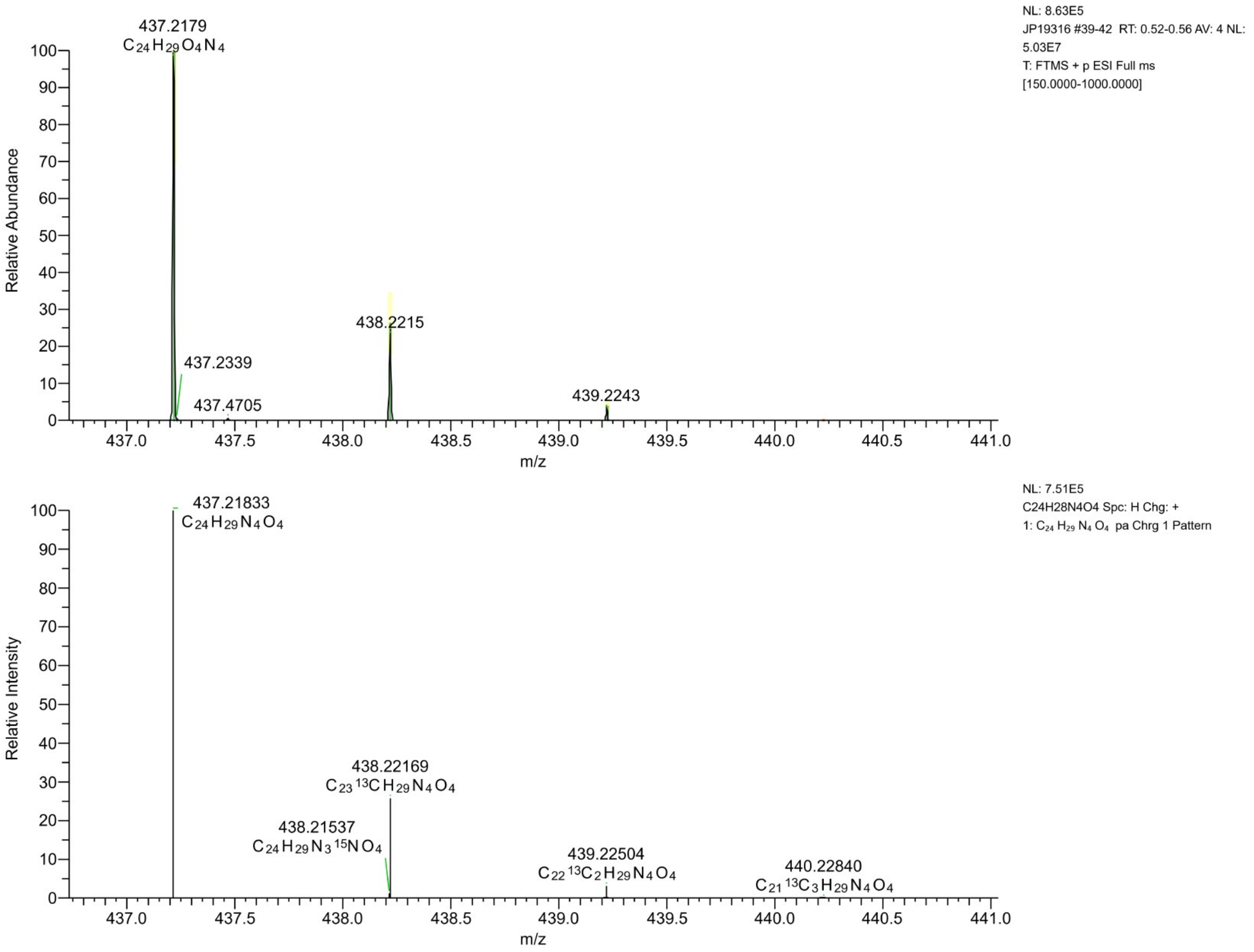

Figure S8-4. HRMS result of **20b** (**JP-193-16**)

(S)-N-(4-amino-2-methoxyphenyl)-4-((7-methoxy-5-oxo-2,3,5,11a-tetrahydro-1H-benzo[e]pyrrolo[1,2-a][1,4]diazepin-8-yl)oxy)butanamide (**20c, JP-193-21**)

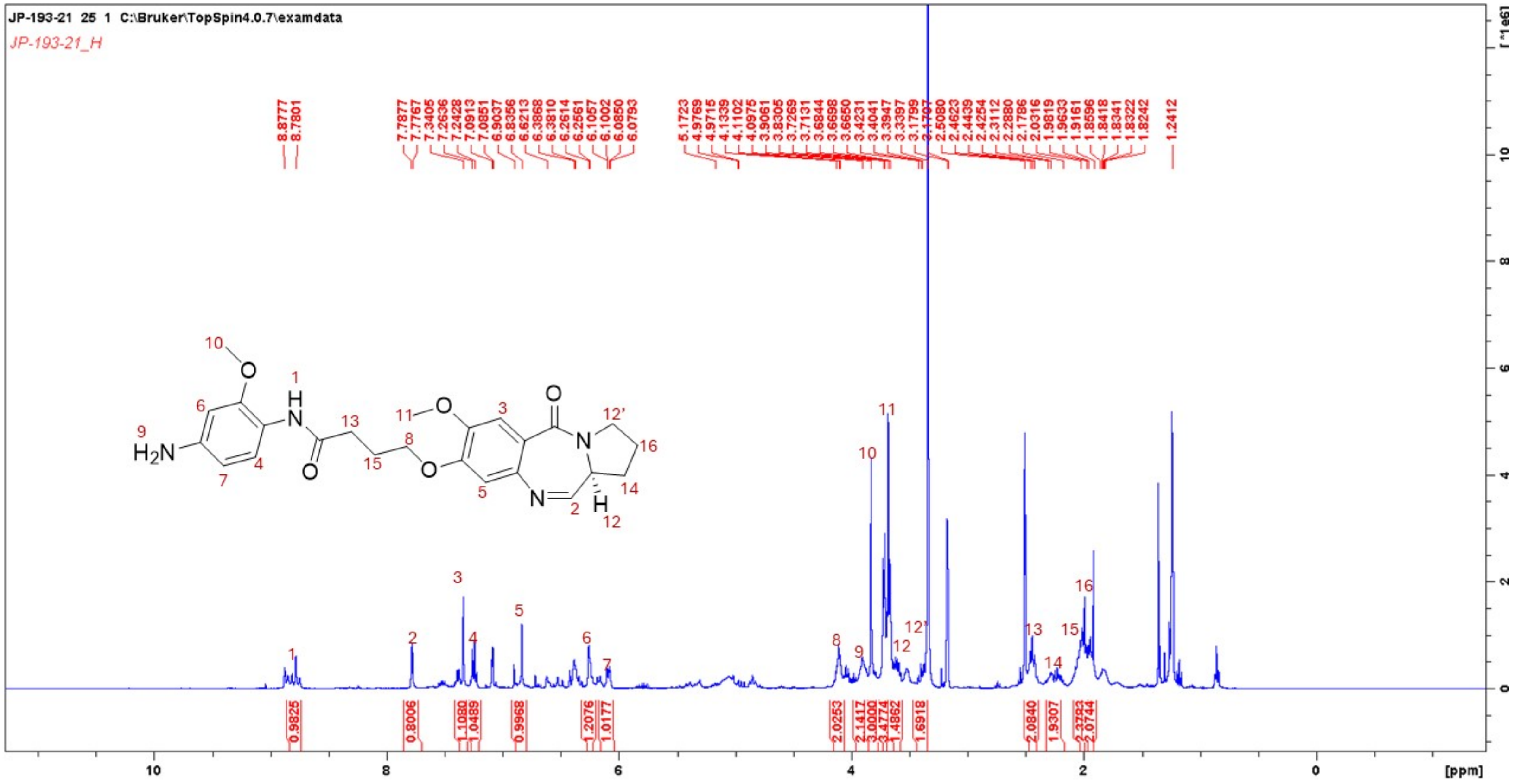

Figure S9-1. Proton NMR of **20c** (**JP-193-21**)

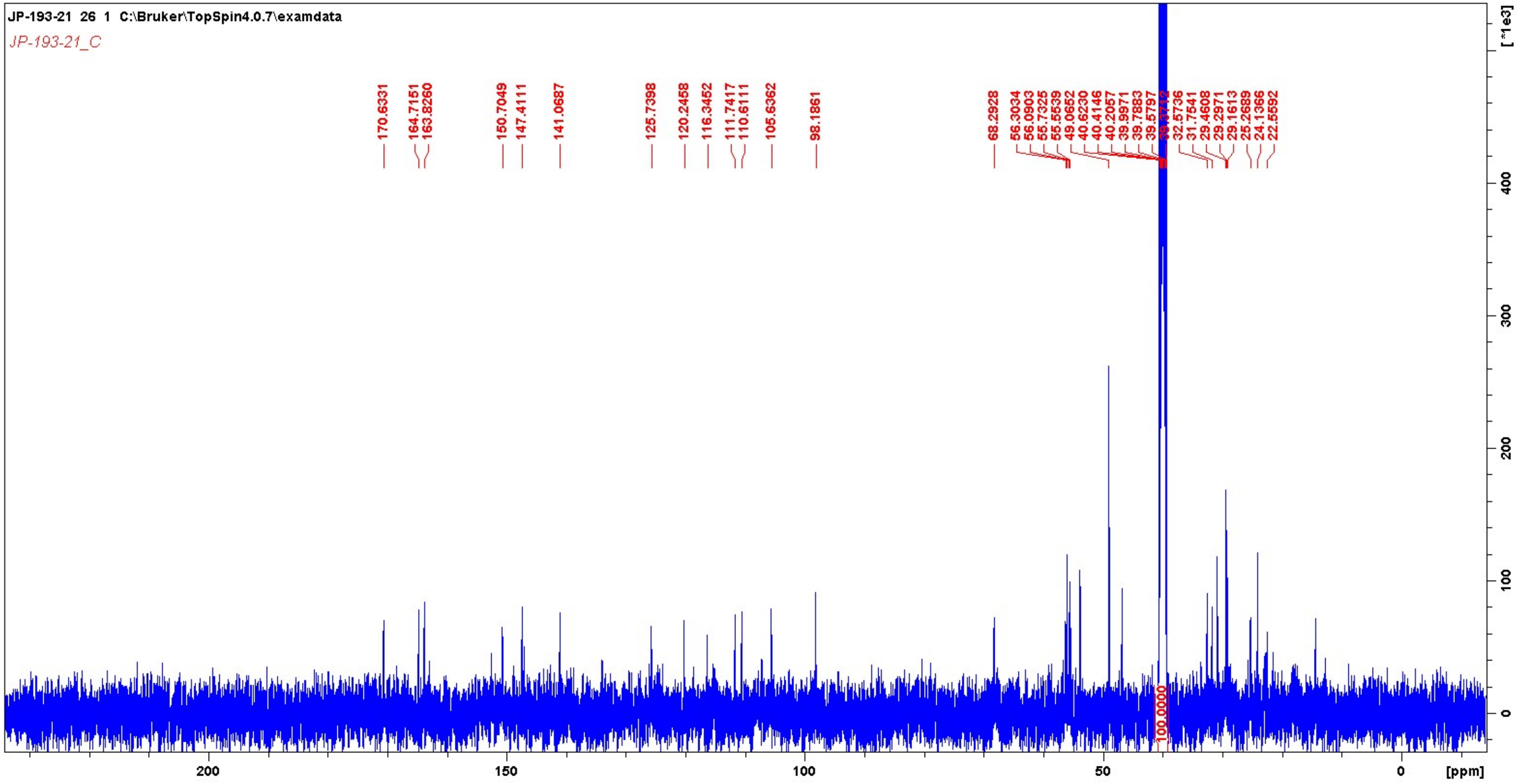

Figure S9-2. Carbon NMR of **20c** (**JP-193-21**)

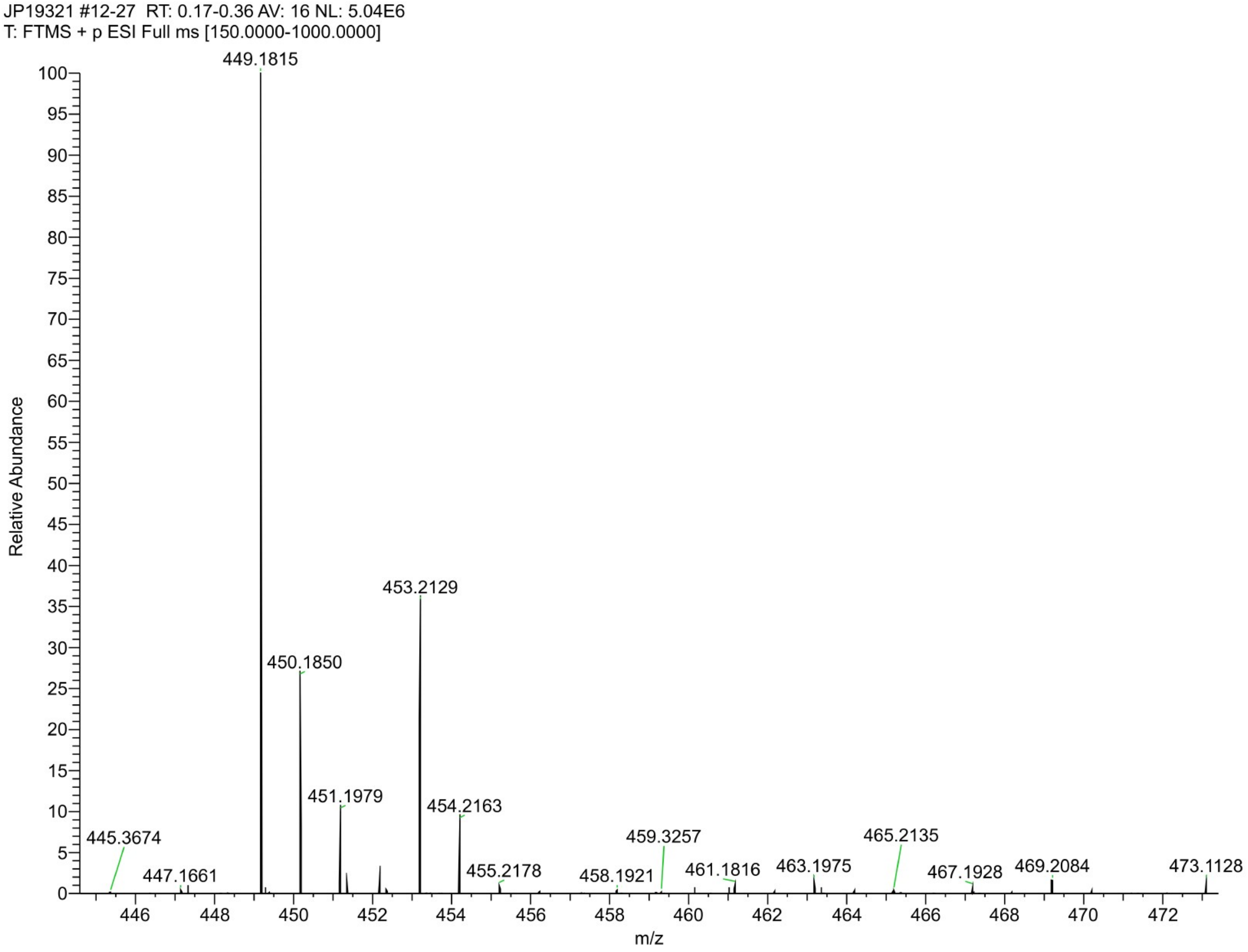

Figure S9-3. HRMS result of **20c** (**JP-193-21**)

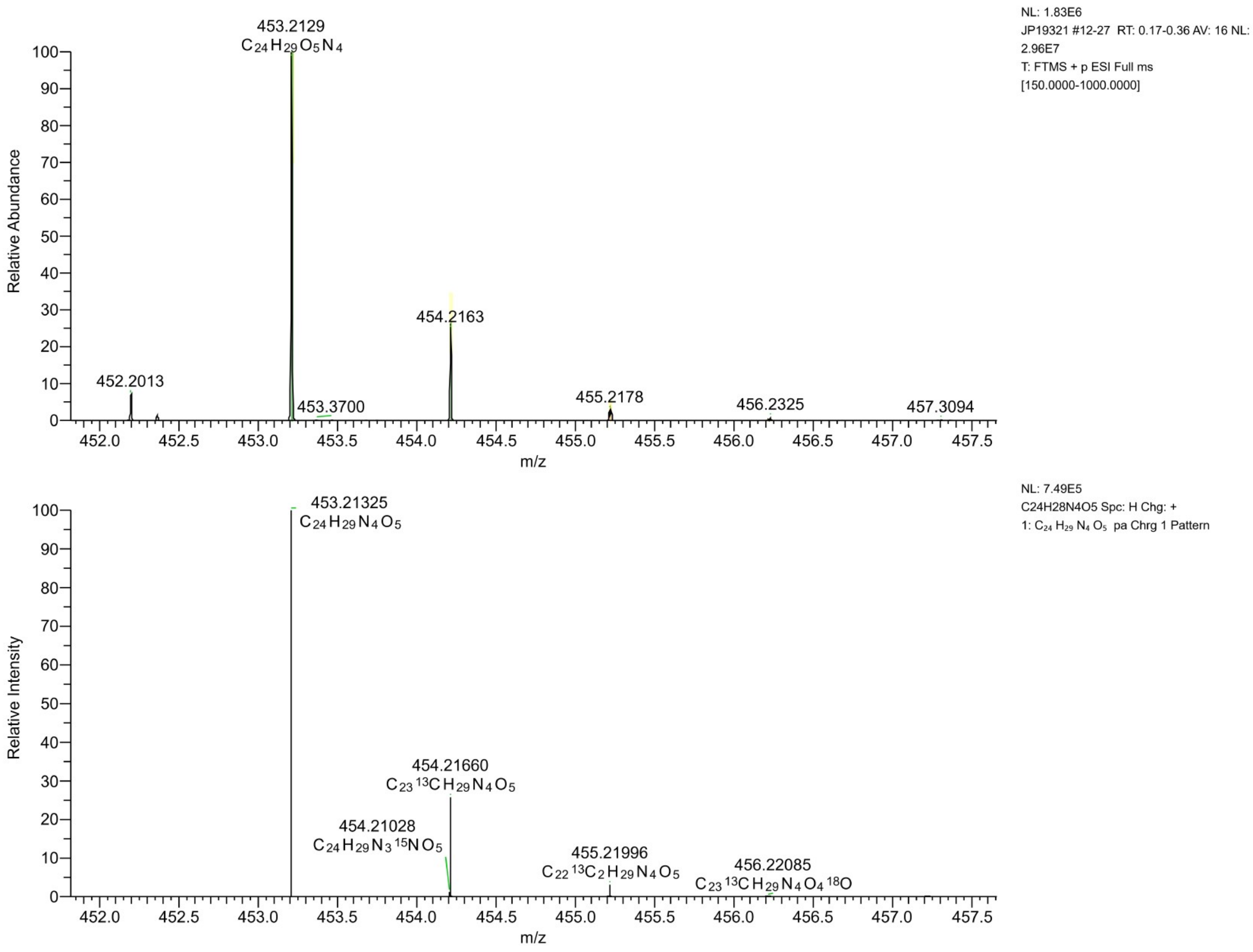

Figure S9-4. HRMS result of **20c** (**JP-193-21**)

Representative Example of HPLC Profile of Some Compounds

Note: the first peak is the solvent front.

Compound **15d** (**JP-163-16/JP-198-13**)

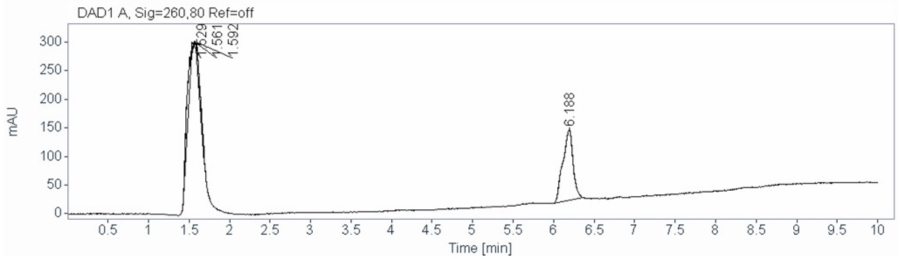

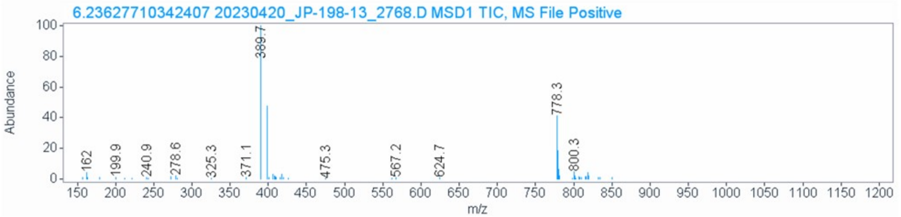

Compound **15a** (**JP-175-P6**)

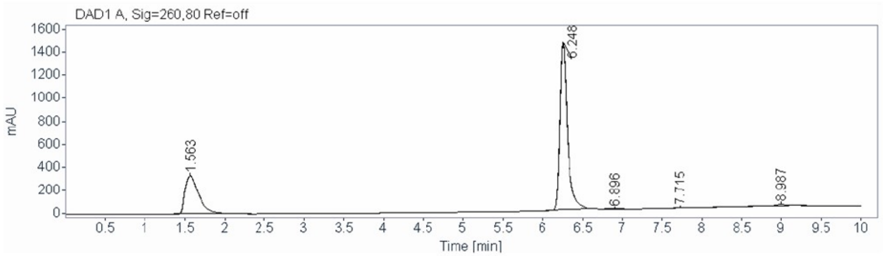

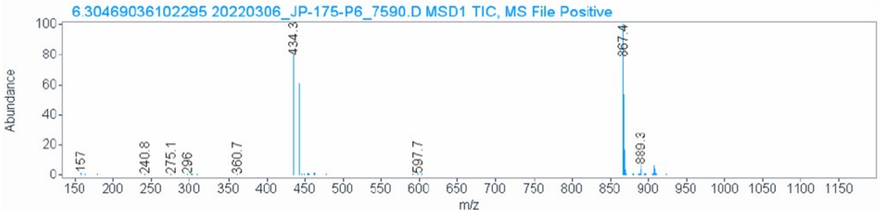

Compound **15b** (**JP-179-P6**)

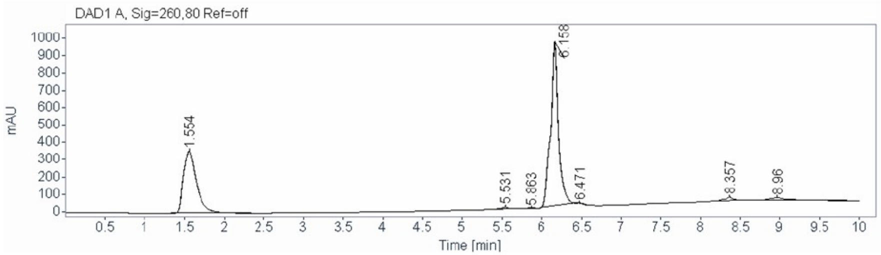

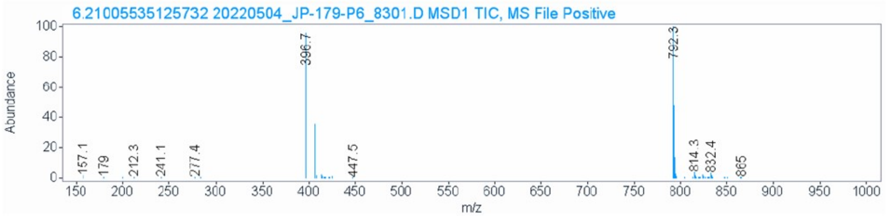

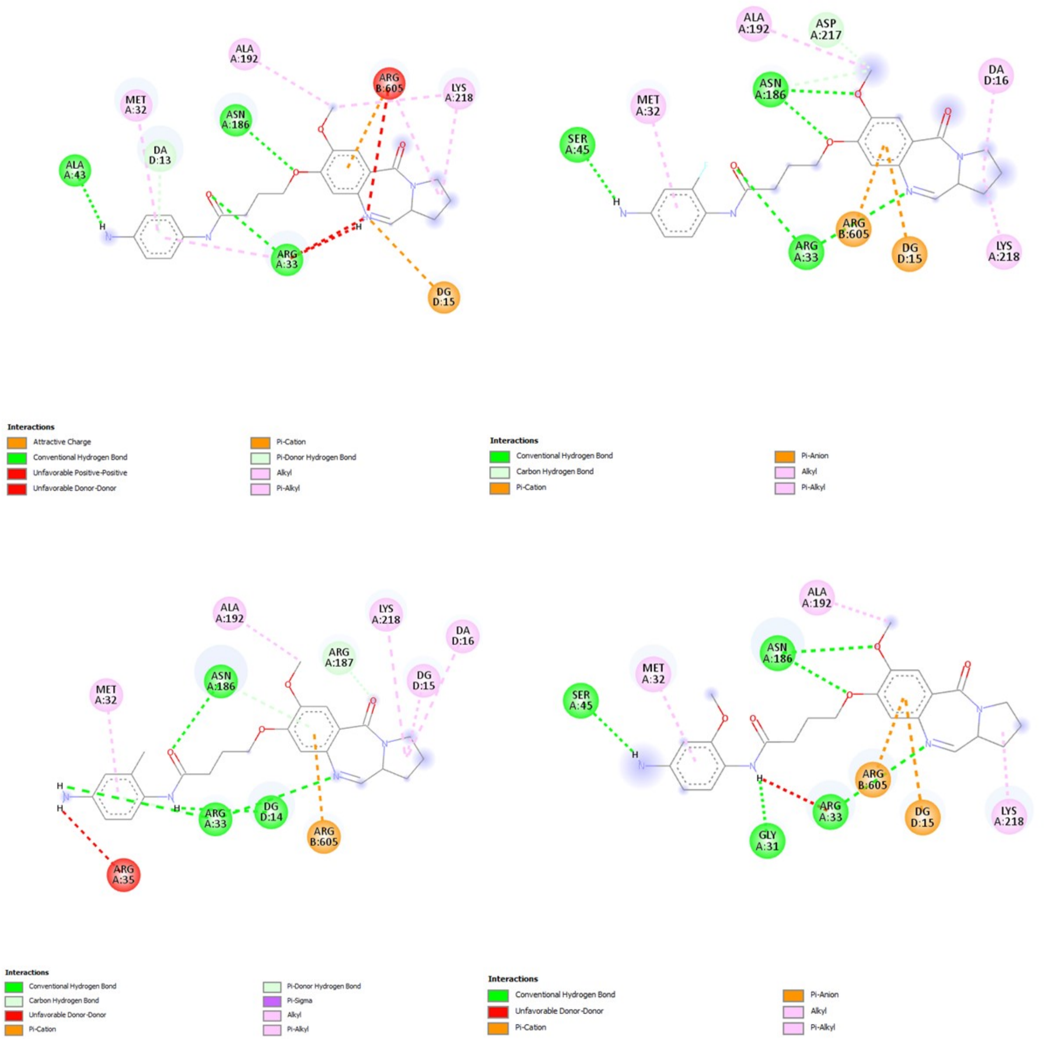

Figure S10. Modelling result of the PBD controls on p65 (PDB: 1VKX). Energy for binding (MMH-165-26: -9.4 Kcal/mol; JP-193-12: -8.4 Kcal/mol; JP-193-16: -9.0 Kcal/mol; JP-193-21: -8.7 Kcal/mol)

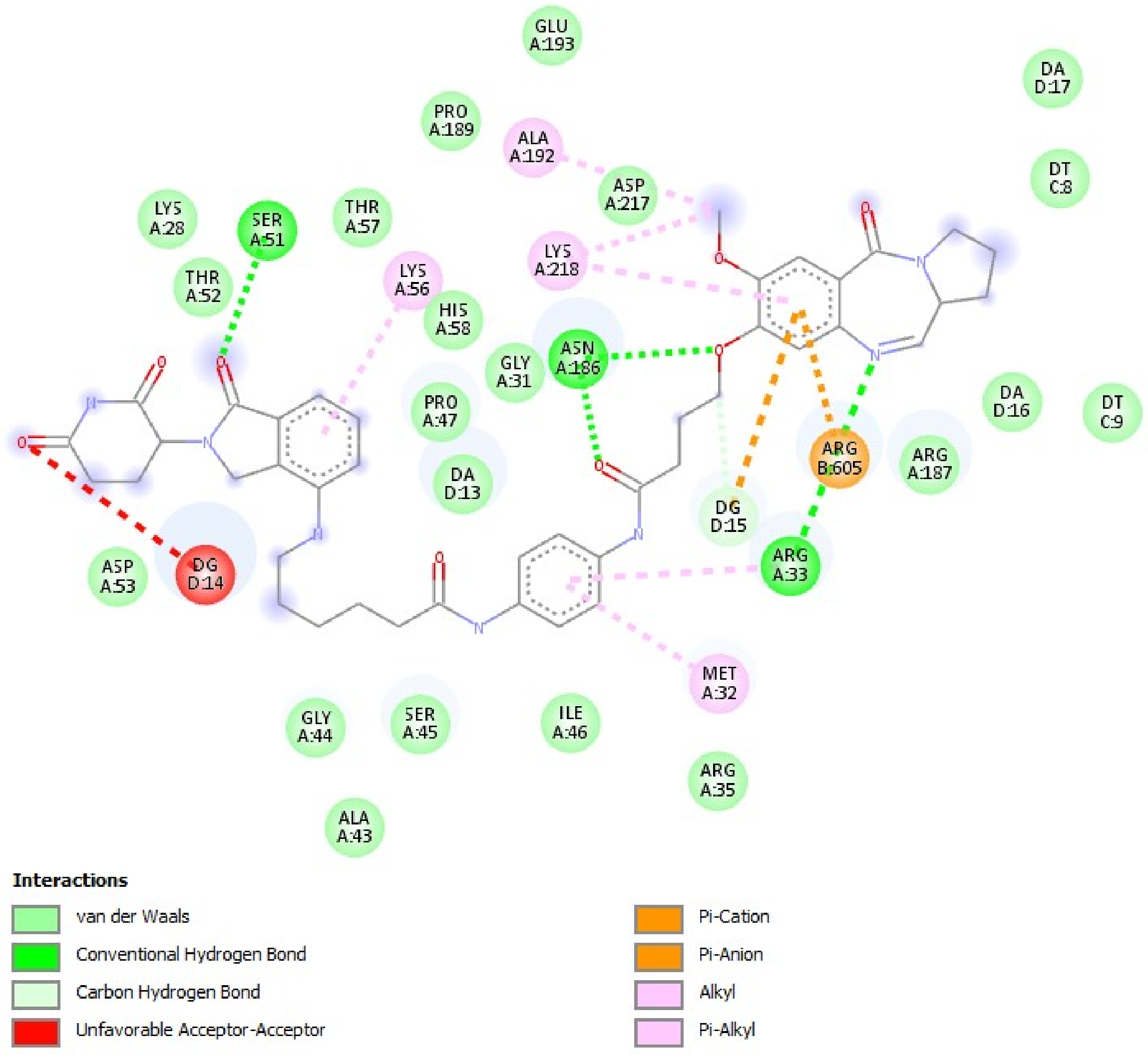

Figure S11. Modelling result of JP-163-16 in 1VKX (2D ligand-protein correlation).

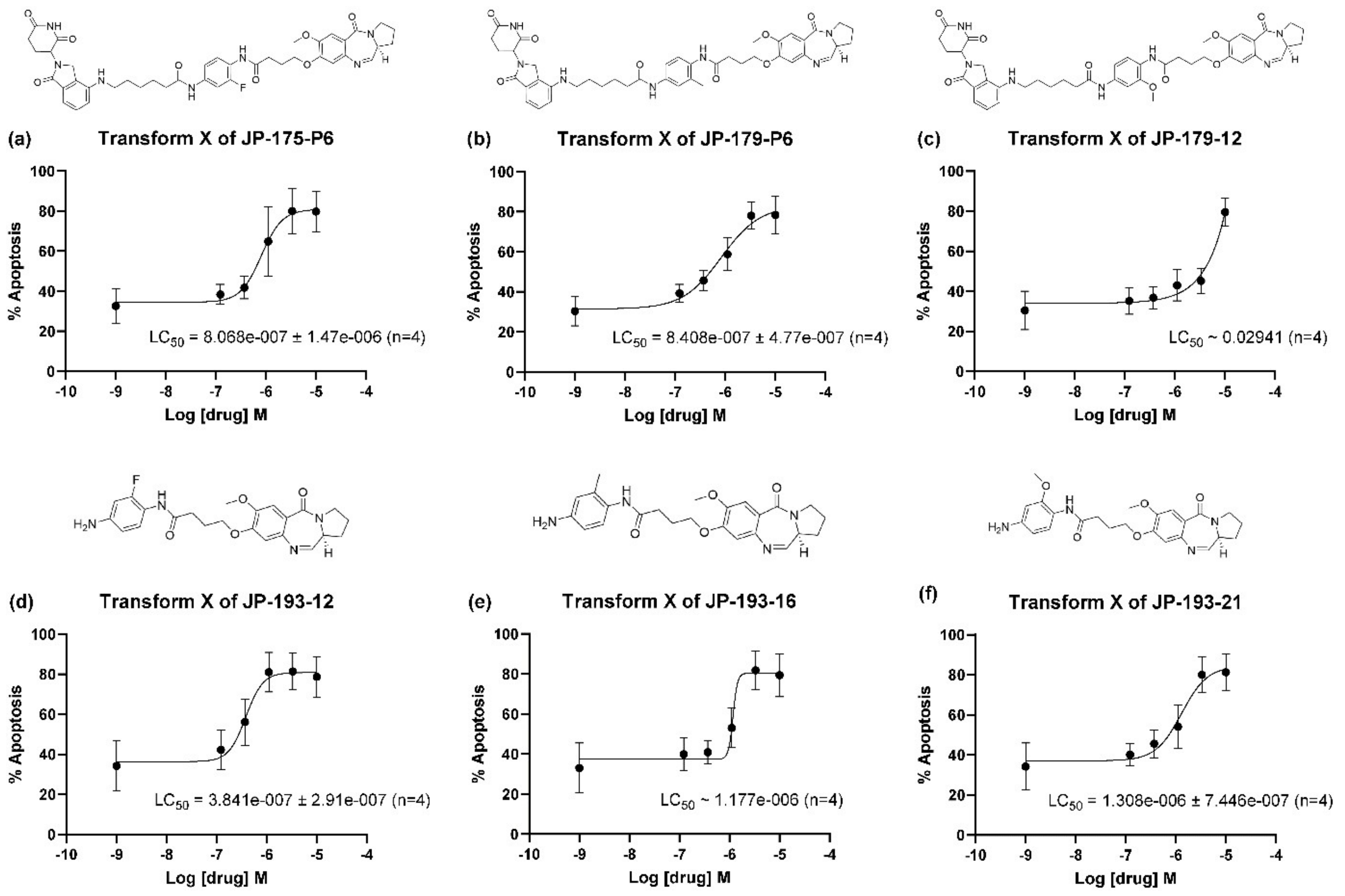

Figure S12. Dose-response curves for a series of PBD-PROTACs and their associated PBD constituent molecules in MEC-1 cells. Dose-response curves were generated using annexin V/7-AAD data following 48h of exposure to each compound. The LD50 values were interpolated from each individual dose-response curve using GraphPad Prism 10. All experiments were performed in 4 times.

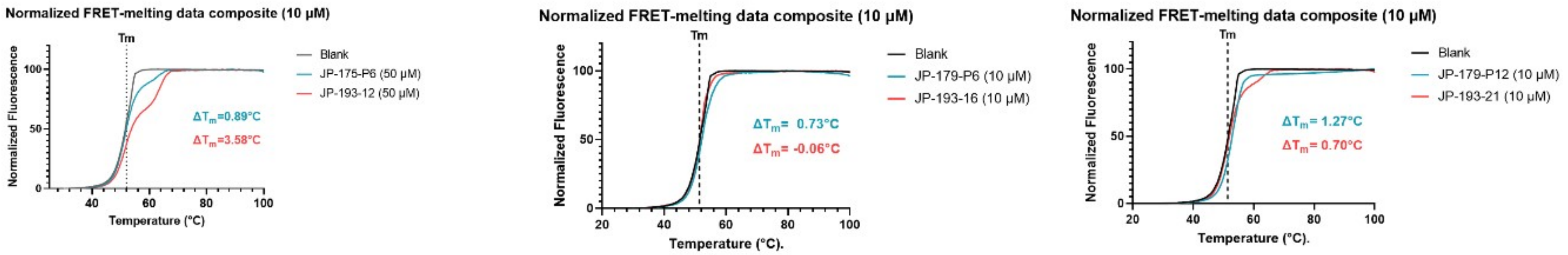

Figure S13. FRET melting assay results reveal that the PROTAC analogues exhibited various levels of DNA-binding capabilities. Each compound was mixed with an AT-rich DNA sequence at 10μM. JP-175-P6, JP-179-P6, and JP-179-P12 all increase the melting temperature.
